# Dual spatially resolved transcriptomics for human host–pathogen colocalization studies in FFPE tissue sections

**DOI:** 10.1186/s13059-023-03080-y

**Published:** 2023-10-19

**Authors:** Hailey Sounart, Enikő Lázár, Yuvarani Masarapu, Jian Wu, Tibor Várkonyi, Tibor Glasz, András Kiss, Erik Borgström, Andrew Hill, Sefanit Rezene, Soham Gupta, Aleksandra Jurek, Anezka Niesnerová, Henrik Druid, Olaf Bergmann, Stefania Giacomello

**Affiliations:** 1 Department of Gene Technology, KTH Royal Institute of Technology, SciLifeLab, Stockholm, Sweden; 2 Department of Cell and Molecular Biology, Karolinska Institutet, Stockholm, Sweden; 3 2nd Department of Pathology, Semmelweis University, Budapest, Hungary; 4 10X Genomics, Pleasanton, CA USA; 5 Department of Laboratory Medicine, Karolinska Institutet, Stockholm, Sweden; 6 Department of Oncology-Pathology, Karolinska Institutet, 17177 Stockholm, Sweden; 7 Center for Regenerative Therapies Dresden (CRTD), TU Dresden, Dresden, Germany; 8 Universitätsmedizin Göttingen, Institute of Pharmacology and Toxicology, Göttingen, Germany

**Keywords:** Spatial transcriptomics, Host–pathogen interactions, Colocalization analysis, Formalin-fixed paraffin-embedded (FFPE) tissues

## Abstract

Technologies to study localized host–pathogen interactions are urgently needed. Here, we present a spatial transcriptomics approach to simultaneously capture host and pathogen transcriptome-wide spatial gene expression information from human formalin-fixed paraffin-embedded (FFPE) tissue sections at a near single-cell resolution. We demonstrate this methodology in lung samples from COVID-19 patients and validate our spatial detection of SARS-CoV-2 against RNAScope and *in situ* sequencing. Host–pathogen colocalization analysis identified putative modulators of SARS-CoV-2 infection in human lung cells. Our approach provides new insights into host response to pathogen infection through the simultaneous, unbiased detection of two transcriptomes in FFPE samples.

**\mentary Information:**

The online version contains supplementary material available at 10.1186/s13059-023-03080-y.

## Background

Much is still unknown about how hosts react to pathogens and how pathogen infection underlies various biological processes and disease states. Although single-cell transcriptomics methods have improved the elucidation of cell type-specific effects caused by pathogens and how these relate to disease outcomes [1, 2], such approaches remove pathogens and host cells from their natural environment, limiting the study of complex spatial dynamics of infections. Gaining insights into the localized host response to pathogen infection requires technologies that allow for the co-capture of host and pathogen spatial transcriptome information. Moreover, there is a need to develop technologies compatible with formalin-fixed paraffin-embedded (FFPE) tissue blocks, since working with potentially infectious human material under standard laboratory conditions requires the neutralization of the pathogens present. The ability to work with FFPE blocks also opens up the possibility of accessing the plethora of archived samples deposited in biobanks.

There are currently several FFPE-compatible spatially-resolved transcriptomics methods available [3–27], however, there are limitations to their use in carrying out host–pathogen colocalization analysis. Several methods with high spatial resolution, such as RNAScope [3], FFPE-smFISH [4], clampFISH 2.0 [5], MERFISH/MERSCOPE [Vizgen] [6, 7], in situ sequencing (ISS) [CARTANA, 10X Genomics] [8–11], Improved in situ sequencing (IISS) [12], Xenium In Situ Technology [10X Genomics] [13, 14], FISSEQ [ReadCoor, 10X Genomics] [15, 16], MOSAICA [17], CosMx [NanoString] [18], and spatially-resolved FFPE microRNA capture [19], are limited by providing only a partial view of the full transcriptome as they employ sets of gene-specific probes that range from around 10 to 6000 genes. Alternatively, some methods that can capture a larger scope of the transcriptomic landscape, such as LCM-seq [20], GeoMx [NanoString] [21, 22], Pick-Seq [23], and RNA-seq of FFPE PuTi-spots [24], are hindered by lower tissue area throughput due to laborious selection of tissue regions of interest [18, 20–24]. Oligo-d(T) based methods, such as DBiT-seq [25, 26] and spatially-resolved FFPE mRNA capture with Visium [27], are widely applicable for eukaryotes, however, these methods are unable to capture the non-polyadenylated transcripts present in many prokaryotic and viral pathogens. In addition, irreversible modifications of the 3’ polyA tail induced by formalin fixation can impact the performance of oligodT-based methods that capture 3’ poly-A-tailed transcripts [28, 29]. To study host–pathogen dynamics in a comprehensive manner, we need an exploratory approach to identify what host genes are affected by the presence of the pathogen through the co-detection of pathogen-specific transcripts.

Several of the FFPE-compatible spatial transcriptomics technologies performed host–pathogen spatial characterization [21]. For instance, spatially resolved analysis of SARS-CoV-2 viral infection in the human lung provided new insights into the heterogeneous viral distribution and host response to the infection [30–36]. However, such approaches largely target a limited number of host and/or pathogen transcripts and rely on the selection of predefined regions of interest that result in a small number of assayed tissue areas and impede an unbiased analysis of whole tissue sections [21]. Other studies performed SARS-CoV-2 detection by targeting a limited number of genes with RNAScope [37] or viral proteins with immunohistochemistry [38] in sections consecutive to those processed for human whole-transcriptome analysis with Spatial Transcriptomics (10X Genomics Visium platform). In a non-probe-based approach, one study captured a single SARS-CoV-2 UMI from a COVID-19 patient using an untargeted, poly-A-based Spatial Transcriptomic (ST) method [27]; however, it is unclear if a lower viral load in the sample or low sensitivity of the method impacted the viral detection. Methods that combine the advantages of using targeted probes to detect transcripts—improving the capture efficiency of less abundant pathogen transcripts in formalin-fixed tissues—with using a whole-transcriptome probe panel for unbiased capture of gene expression information would enable host and pathogen transcriptome-wide dual RNA analysis.

Outside of pathogen-specific studies, such as those focused on SARS-CoV-2 in COVID-19 samples, researchers developed novel methods to analyze the interactions between host cells and the local microbiome with a high spatial resolution (100–55 µm) [39, 40]. These approaches provide insights into the composition and abundance of different microbial taxa present in the tissue microenvironment, along with transcriptome-wide host gene expression [39, 40]; however, such methods [39, 40] are unable to explore individual gene expression profiles of each microbe or pathogen. Additionally, these studies [39, 40] demonstrated the methods on fresh frozen tissues, and have not yet applied the approaches to FFPE tissue sections. In the case of infectious diseases caused by a specific pathogen, a spatially-resolved transcriptomics technology that captures both host and pathogen transcriptome-wide information in whole FFPE tissue sections at high spatial resolution would provide more in-depth information.

Here, we present a spatial transcriptomics strategy to unbiasedly explore host–pathogen interactions in FFPE tissues. We utilized the commercially available, high-throughput, sequencing-based Spatial Transcriptomics (ST) platform [41, 42] and introduced the co-detection of a second, pathogen transcriptome to the human one. We demonstrated the potential of such an approach through the dual capture of human and SARS-CoV-2 viral transcriptomes at 55 µm (~ 1–10 cells) spatial resolution in FFPE sections of COVID-19 patient lungs. Targeted transcriptome technologies RNAScope [3] and in situ sequencing (ISS) [8–10] validated our spatial detection of SARS-CoV-2. Our approach reaches a high spatial resolution (55 µm), is designed to spatially resolve 10 SARS-CoV-2 transcripts, and does not require the preselection of regions of interest, facilitating unbiased, whole-tissue analysis. A prominent feature of our method is the colocalization analysis of human and viral gene expression information that allows an understanding of human tissue response to SARS-CoV-2 infection by comparing areas with and without the presence of viral RNA in the same tissue section. Overall, our strategy opens up the possibility of spatially studying host response to pathogen infections in various infectious diseases through the simultaneous, unbiased detection of two transcriptomes.

## Results

### Dual Spatial Transcriptomics enables simultaneous, accurate detection of both host and pathogen whole transcriptomes in FFPE tissue sections

**Fig. 1:**
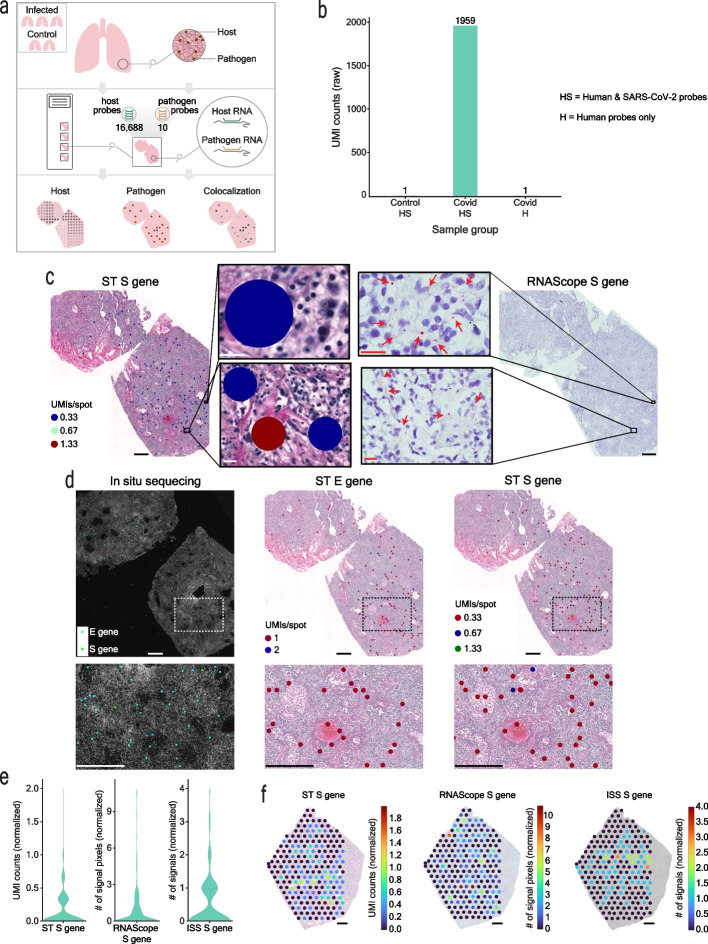
Spatial Transcriptomics SARS-CoV-2 capture validated by RNAScope and in situ sequencing (ISS). **a** Overview of the study. 16,688 human host genes and 10 SARS-CoV-2 pathogen genes were assayed across 3 lung samples from SARS-CoV-2 infected patients and 2 lung samples from control patients. **b** SARS-CoV-2 detection in control sections with human and SARS-CoV-2 probes added (HS), in COVID-19 sections with human and SARS-CoV-2 probes added (HS), and in COVID-19 sections with only human probes added (H). **c** SARS-CoV-2 S gene detection by ST and RNAScope in consecutive sections of the 1C sample. Full tissue section scale bars are 550 µm and zoomed-in panel scale bars are 20 µm. **d** Detection of SARS-CoV-2 S and E genes by ST and in situ sequencing (ISS). All scale bars are 550 µm. **e** Distribution of ST S gene, RNAScope S gene, and ISS S gene signals across sample 1C. **f** SARS-CoV-2 ST S gene, RNAScope S gene, and ISS S gene signals across a portion of sample 1C. Scale bars are 900 µm

We advanced the Visium Spatial Gene Expression assay for FFPE tissues [42] to simultaneously capture human and SARS-CoV-2 whole transcriptome (WT) information at a 55 µm resolution. Specifically, we analyzed 16,688 human genes and 10 SARS-CoV-2 gene transcripts in total, across 13 lung tissue sections from 5 lung tissue samples, 3 from COVID-19 patients (i.e., 1C, 2C, 3C), and 2 from control patients (i.e., 4nC, 5nC) (Fig. 1a, Additional file 1: Fig. S1, Additional file 2: Table S1, Additional file 3: Table S2). First, we verified the specificity of the SARS-CoV-2 probes (S) for capturing SARS-CoV-2 transcripts only by applying both human WT probes (H) and spike-in SARS-CoV-2 probes (HS) to control tissue sections. We did not identify any SARS-CoV-2 transcripts above background levels in these samples (see Methods), demonstrating that the SARS-CoV-2 probes specifically captured only SARS-CoV-2 information (Fig. 1b, Additional file 1: Fig. S2).

To independently validate the viral detection by our set of SARS-CoV-2 probes, we compared the ST viral signal to the signal obtained by orthogonal imaging-based spatial RNAScope technology [3] in consecutive sections. Specifically, we compared the distribution of spots with detected S gene signals by ST and RNAScope across all COVID-19 and control samples. To systemically and unbiasedly analyze all our samples, we developed a computational pipeline for automated signal detection across both platforms (see Methods and Additional file 1: Fig. S3-S5). Using our computational approach, we found an average specificity of the ST method of 94.92% (1C: 86.86%, 2C: 99.37%, 3C: 98.53%) (Fig. 1c). Furthermore, we performed a second validation of the ST detection of the S and E genes using in situ sequencing (ISS) [8–10] in the same sample (1C) and with the same automated pipeline (see Methods). Despite the substantial distance (~ 300 µm) between the sections used for the ST and ISS experiments, we observed an overall similar distribution of the viral signal and an average specificity of 82.20% of the ST method in comparison to ISS (E gene: 83.65%, S gene: 80.74%) (Fig. 1d, Additional file 1: Fig. S6), confirming that our platform can capture SARS-CoV-2 information accurately. Furthermore, quantification of the RNAScope and ISS S gene signals in comparison to the ST S gene-specific signal in the COVID-19 lung sample with the highest viral transcript levels, 1C, yielded a similar signal intensity distribution and spatial pattern in the S gene heatmap between the three detection methods (Fig. 1e,f). Quantitative comparison of the RNAScope and ST S gene signals resulted in a specificity of 76.37% and a sensitivity of 28.93%, yielding a higher sensitivity than that of the original ST method [41] compared to the single molecule detection method smFISH [43]. The two methods did not yield statistically significant differences in their viral signal detection (*p*-value = 0.323, see Additional file 1: Table S3), providing further support to the validation of our ST-based SARS-CoV-2 gene detection with RNAScope. Additional qPCR validation targeting the SARS-CoV-2 E gene showed a similar trend of SARS-CoV-2 related transcripts between the samples to the cumulative signal obtained by the spatial detection methods (ST, RNAScope) (Additional file 1: Fig. S7). The agreement between several alternative validation techniques and the ST-based detection provides further confirmation of the accurate capture of SARS-CoV-2 transcript by our method.

**Fig. 2:**
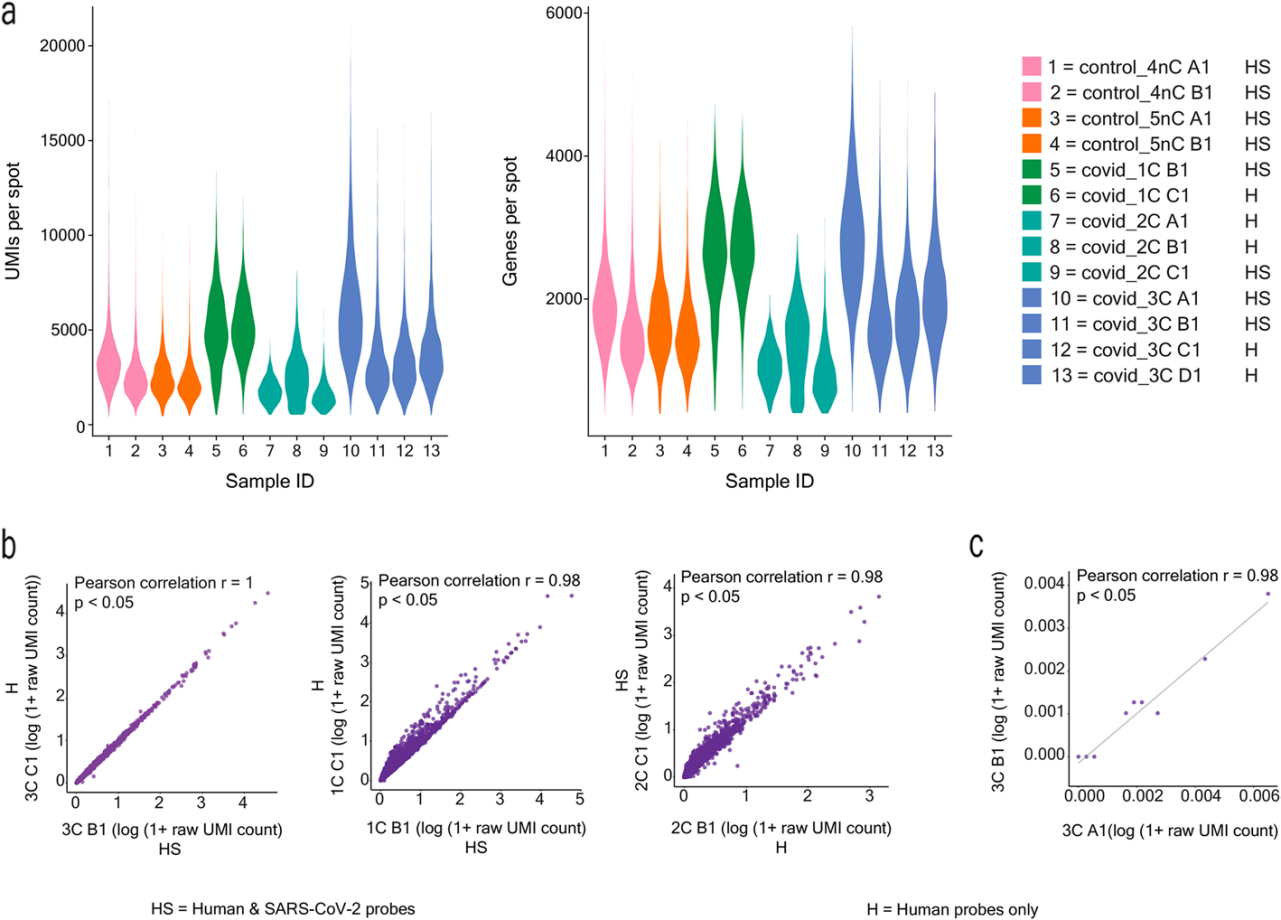
Reproducible capture of human and SARS-CoV-2 transcriptomes. **a** Distribution of UMI and gene counts per spot across patient sample sections. **b** Pearson correlation of average human gene expression between consecutive sections for each sample, one section with human and SARS-CoV-2 probes added (HS) and the other with only human probes added (H), *p*-value < 0.05. **c** Pearson correlation of average SARS-CoV-2 transcriptome capture between consecutive sections, *p*-value < 0.05

Subsequently, we sought to understand if the addition of the SARS-CoV-2 probes impacted the quality of the human gene expression information captured. To this end, we analyzed consecutive COVID-19 sections with both human WT probes and spike-in SARS-CoV-2 probes (HS) versus only human WT probes (H). Across the COVID-19 and control tissue sections, we generated a dataset consisting of 37,754 spots in total, with an average of ∼2,013 unique human genes and ∼3,809 unique human molecules (UMIs) per spot, respectively (Fig. 2a). We captured very similar human gene expression profiles between sample replicate sections and across most samples, both with and without SARS-CoV-2 probes added (*r* = 0.98–1, *p*-value < 0.05) (Fig. 2a-b, Additional file 1: Fig. S8). Furthermore, we observed a lack of correlation between SARS-CoV-2 UMI counts and human average UMIs per spot (*r* = 0.06, *p*-value < 0.05) (Additional file 1: Fig. S9a). Overall, these results demonstrate the highly reproducible capture of human transcriptomic information and the specificity of the SARS-CoV-2 probes in detecting the SARS-CoV-2 transcriptome without interfering with the capture efficiency of the human transcripts.

### Host–pathogen transcriptome co-capture enables exploration of pathogen spatial gene distributions in diseased tissue sections

**Fig. 3:**
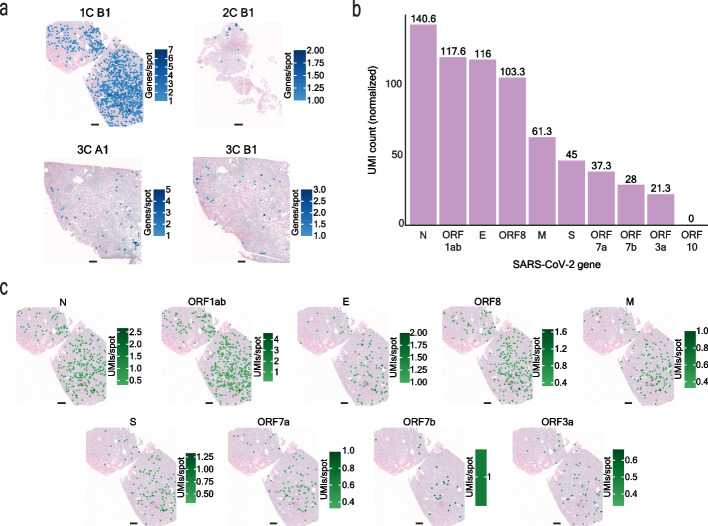
Spatial distribution and abundance of SARS-CoV-2 gene transcripts. **a** SARS-CoV-2 genes per spot across each COVID-19 sample. Scale bars are 500 µm. **b** Abundance (total normalized UMI counts) of the SARS-CoV-2 genes across all COVID-19 samples. **c** Spatial distribution of UMI counts per spot of each SARS-CoV-2 gene for COVID-19 sample 1C. Scale bars are 500 µm

We next investigated the SARS-CoV-2 transcriptome profile across our COVID-19 samples. In COVID-19 sections, 9.5% of spots (i.e., 1,132 spots in total) presented a SARS-CoV-2 transcriptional signal with highly reproducible capture of SARS-CoV-2 gene expression between consecutive sections (*r* = 0.98, *p*-value < 0.05, Fig. 2c) and a high correlation of the SARS-CoV-2 gene expression (as UMI counts) in a spot to the average SARS-CoV-2 expression in a spot (*r* = 0.96, *p*-value < 0.05) (Additional file 1: Fig. S9b). Overall, we captured 90% (i.e., 9 out of 10) of the targeted SARS-CoV-2 genes (Additional file 4: Table S4) with an average of ∼1.7 unique molecules and ∼1.5 unique genes per spot, respectively, across samples. These relatively low levels of detected viral transcripts demonstrate the high sensitivity of our approach and are likely associated with the longer disease duration (13–17 days) of the patients included in the analysis (Additional file 3: Table S2), in agreement with several studies that observed lower, or even undetectable, viral load in COVID-19 patients with longer survival times [30, 31, 33, 36, 44]. The overall distribution of SARS-CoV-2^+^ spots in COVID-19 samples showed a wide range of SARS-CoV-2^+^ spot ratios across the COVID-19 samples: 33.6% for 1C, 1.1% for 2C, and 1.0–1.6% for 3C (Fig. 3a, Additional file 4: Table S4), congruent to the heterogeneous viral loads across different samples in similar disease stages observed in other studies [30, 33, 35, 36]. In addition, we observed varied abundances of the different SARS-CoV-2 gene transcripts across the three COVID-19 samples (Fig. 3b, Additional file 1: Fig. S10). For example, N was the highest expressed SARS-CoV-2 gene, while ORF10 was not detected at all, in line with previous reports of N as the most abundant subgenomic RNA (sgRNA) [45, 46] and ORF10 as consistently either absent or the scarcest sgRNA detected [45, 46]. The abundance trend of the remaining SARS-CoV-2 genes (M, E, S, ORF1ab, ORF3a, ORF7a, ORF7b, ORF8) varied across the four COVID-19 sample sections (Additional file 1: Fig. S10), with the factors driving these differences remaining to be further investigated. SARS-CoV-2 genes that were more abundant exhibited a higher number of spots with only that gene detected (termed singleton spots) (Additional file 1: Fig. S11). However, many significantly colocalizing genes, such as gene pairs S–N, ORF8-N, ORF7a-ORF8, ORF7a-N, ORF1ab-S, ORF1ab-ORF8, ORF1ab-ORF3a, ORF1ab-N, ORF1ab-M (*p*-value < 0.05, Additional file 1: Fig. S12), were also genes with higher total UMI counts and were found in a greater number of spots. Two of these genes (N and ORF8) are expected to have higher sgRNA abundances and several genes (ORF1ab, S, ORF3a, and M) are localized in closer proximity on the SARS-CoV-2 genomic RNA (gRNA) [45, 46]. Thus, the relative abundance of gRNA, sgRNAs, and the physical proximity of genes on the gRNA could be impacting the SARS-CoV-2 gene colocalization in the ST spots. Although the SARS-CoV-2 transcripts differed in their abundances across genes, we observed a fairly even spatial distribution of each gene across samples 1C and 3C, while for 2C the transcripts showed a more localized spatial distribution (Fig. 3c, Additional file 1: Fig. S13-S14). Variation in the SARS-CoV-2 gene abundances and colocalization could be influenced by the different number of gene-specific probes across the SARS-CoV-2 genes, RNA quality, and the SARS-CoV-2 probes binding to both viral genomic RNA (gRNA) and subgenomic RNA (sgRNA). Previous studies observed sgRNA abundance variation [45, 46] and such differences could be reflected in our SARS-CoV-2 transcriptomic data.

### The human lung spatial transcriptome during COVID-19 infection

**Fig. 4:**
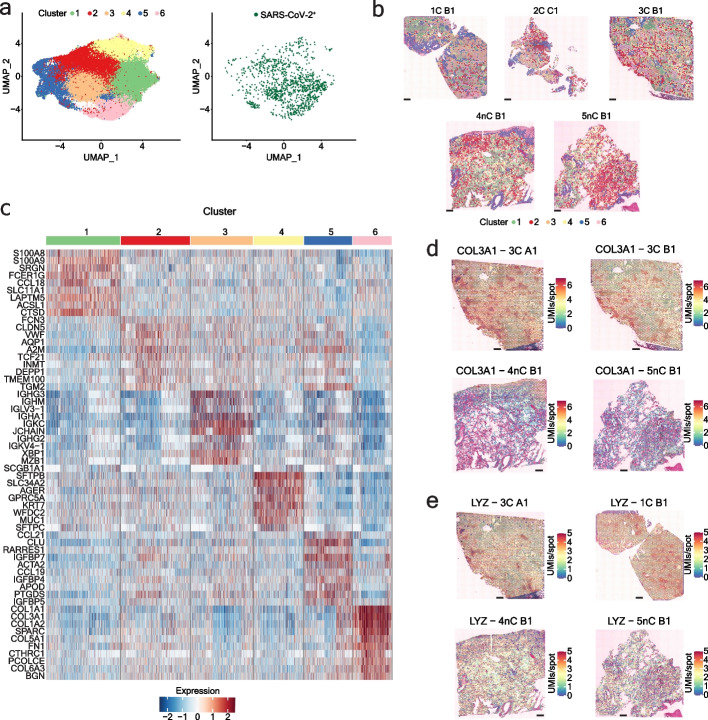
Human lung cellular landscape in response to SARS-CoV-2 infection. **a** Clustering of the human transcriptome data across COVID-19 and control sections reveals 6 distinct clusters with SARS-CoV-2^+^ spots distributed throughout the clusters. **b** Differential genes per cluster across COVID-19 and control sections. **c** Spatial distribution of the clusters on COVID-19 and control sections. Scale bars are 500 µm. **d,** **e** Spatial distribution of genes upregulated in COVID-19 sections, COL3A1 (**d**) and LYZ (**e**). Scale bars are 500 µm. Differential expression analysis used the Wilcoxon rank sum test and “bimod” Likelihood-ratio tests, *p*-value < 0.05

By simultaneously capturing the spatial human and SARS-CoV-2 transcriptomes, we were able to perform both unsupervised and supervised analyses of the infection pattern. First, we explored the human lung cellular landscape in response to SARS-CoV-2 infection by unsupervised, joint graph-based clustering of the spatial transcriptomics data collected from both COVID-19 and control sections and identified six distinct clusters (Fig. 4a). Investigation into the cluster marker genes revealed mixtures of different cell types, however, we identified clear expression signatures for clusters dominated by myeloid cells (cluster 1), endothelial cells (cluster 2), B-cells/plasma cells (cluster 3), epithelial cells (cluster 4), and fibroblasts (cluster 6) (Fig. 4b-c, Additional file 1: Fig. S15, Additional file 5: Table S5) (differential expression analysis used the Wilcoxon rank sum test and “bimod” Likelihood-ratio tests, *p*-value < 0.05). Marker genes specific for fibroblast, smooth muscle, and endothelial cells characterized cluster 5, and further subclustering resulted in three subclusters (subcluster 1: fibroblast-dominated, subcluster 2: smooth muscle cell-dominated, subcluster 3: a mixture of endothelial and immune cells), in line with our previous observations (Additional file 6: Table S6).

To identify the proportions of cell types in our ST spots, we performed cell type deconvolution of our ST data using a publicly available single-cell dataset [36]. We found various cell type compositions in our spatial spots, however, the dominant cell type identified per ST cluster largely confirmed our original annotation, purely based on DE analysis of the clusters (Additional file 1: Fig. S16). In addition, the deconvolution analysis uncovered less abundant cell types, such as T/NK cells, that were almost equally distributed between the ST myeloid-enriched cluster 1 and B cell/plasma cell-enriched cluster 3 (Additional file 1: Fig. S16). Re-annotation of the spatial spots based on the dominant cell type present resulted in 11 deconvolution-based clusters (myeloid, endothelial, B/plasma, epithelial, fibroblast, vascular contractile, ciliated, mesothelial, RBC, secretory, T/NK and mast cells), in line with the number of cell types used for label transfer.

We next explored the distribution of the SARS-CoV-2 transcripts across the different tissue regions (clusters). SARS-CoV-2^+^ spots appeared throughout different morphological areas and ST clusters (Fig. 4a), in agreement with the uniform spatial distribution of the SARS-CoV-2 genes across the same tissue sections (Fig. 3a, Additional file 1: Fig. S13-S14). However, a pronounced enrichment of SARS-CoV-2 RNA appeared in ST clusters 1 (myeloid-enriched) and 3 (B cell/plasma cell-enriched) and in the T/NK cell-dominated and B cell/plasma cell-dominated deconvolution-based clusters (Additional file 1: Fig. S17). Endothelial cell-, myeloid cell-, and red blood cell-dominated deconvolution-based clusters showed moderate SARS-CoV-2 enrichment, in line with previous reports [36] (Additional file 1: Fig. S17). We also observed a higher enrichment of SARS-CoV-2 transcripts in the ST cluster 6 (fibroblast-enriched), in contrast to the very low viral enrichment in the fibroblast-dominated deconvolution-based cluster, which suggests that the SARS-CoV-2 RNA in the fibroblast-enriched ST cluster might originate from another cell type and is masked by the gene signature of activated fibroblasts. Of note, epithelial and ciliated cell-dominated clusters, previously described as major targets of the virus [36, 47–51], had very low levels of viral transcripts, likely explained by the analyzed cases being of a late disease phase.

To investigate the transcriptomic shifts within infected lungs, we ran differential expression analysis comparing COVID-19 and control lung sections (differential expression analysis used Wilcoxon rank sum test and “bimod” Likelihood-ratio tests, *p*-value < 0.05). All COVID-19 lung samples included in this study represented the late-phase pneumonia stage (between 13–17 days post-infection) of the disease and showed consistent histopathological features with organizing diffuse alveolar damage, extensive fibrosis, and leukocyte infiltration, accompanied by low viral load [30, 33, 44] (Additional file 1: Fig. S1). We found that the transcriptome data also reflected these substantial structural differences between the COVID-19 and control lung sections. Specifically, signatures of plasma cells (IGHG3, IGKC, IGHM, JCHAIN, IGHG2, IGKV4-1, IGLV3-1, IGHA1), activated fibroblasts (COL1A1, COL1A2, COL3A1), inflammatory cytokines (CXCL9, CCL18) and complement factors (C1QB, C1QC) dominated the DE genes for the COVID-19 lung sections (Fig. 4d, Additional file 7: Table S7). Such transcriptomic changes reflect the overall tissue response to a prolonged SARS-CoV-2 infection.

We further explored the human host transcriptomic landscape within specific clusters (differential expression analysis used the Wilcoxon rank sum test and “bimod” Likelihood-ratio tests, *p*-value < 0.05). We observed marked differences in monocyte-macrophage (CD163, F13A1, CD14, LYZ, APOE, C1QA, B2M, PPARG, VCAN, FCN1, YAP1, FCGR3A) marker genes within the myeloid cell-rich ST cluster 1, activated fibroblast (COL1A1, COL3A1, COL5A1, SPP1, FN1, POSTN, CTHRC) marker genes in the fibroblast-rich ST cluster 6, and AT2 cell (SFTPC, LYZ, MUC1, SLC34A2, LAMP3, PGC, NAPSA, CEBPA, LPCAT1, SDC1, NKX2-1, ABCA3, ALPL) marker genes within the epithelial cell-dominated ST cluster 4 in the COVID-19 lung samples (Additional file 8: Table S8), consistent with substantial cell state and cell type composition changes occurring during the progression of the disease, previously reported in numerous studies [30, 36, 52–54]. Taken together, we observed differences in pathogen abundance and could explore shifts in human gene expression within different tissue compartments.

### Colocalization analysis of human and SARS-CoV-2 transcriptomes identifies novel potential biomarkers

**Fig. 5:**
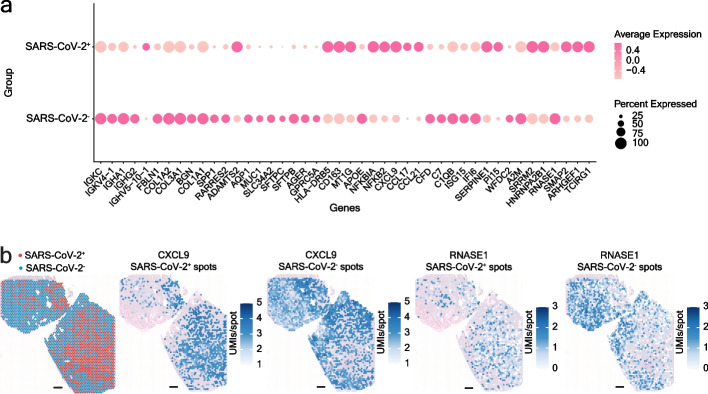
Colocalization analysis reveals human host response to SARS-CoV-2 infection. **a** Dot plot depicting differential expression of human genes in SARS-CoV-2^+^ and SARS-CoV-2^-^ spots in COVID-19 sections. **b** Spatial distribution of RNase1, downregulated in SARS-CoV-2+ spots, and CXCL9, upregulated in SARS-CoV-2^+^ spots, in COVID-19 sample 1C. Scale bars are 500 µm. Differential expression analysis used the Wilcoxon rank sum test and DESeq2 negative binomial distribution tests, *p*-value < 0.05

With the possibility of simultaneously capturing the human and SARS-CoV-2 spatial transcriptomes, our approach allowed us to conduct colocalization analysis and identify host gene expression changes caused by the presence of the viral mRNA in lung cells at 55 µm resolution. By comparing human gene expression patterns between SARS-CoV-2^+^ and SARS-CoV-2^−^ spots *en masse* in COVID-19 tissue sections (Additional file 9: Table S9), we detected a downregulation of certain immunoglobulin genes (IGKC, IGKV4-1, IGHA1, IGHG2), extracellular matrix components (FBLN, COL1A2, COL3A1, BGN, COL1A1, SPP1), as well as several AT2 (SFTPB, SFTPC, MUC1, SLC34A2), AT1 (GPRC5A, AGER), and the alveolar endothelial cell marker AQP1 in the SARS-CoV-2^+^ spots (Fig. 5a) (differential expression analysis used the Wilcoxon rank sum test and DESeq2 negative binomial distribution tests, *p*-value < 0.05). The viral infection preceding both the extensive plasma cell infiltration and fibroblast activation in time and the functional impairment or increased apoptosis of alveolar epithelial cells, known to be the primary cellular targets of SARS-CoV-2 in the lungs [36, 51], may explain these expression patterns. Notably and in line with other works exploring COVID-19-induced transcriptome changes in a spatial context [36], we observed the downregulation of the RNase1 gene in SARS-CoV-2^+^ spots, potentially blocking the degradation of viral RNA in the environment of actively infected cells (Fig. 5a-b, Additional file 9: Table S9).

We next looked into specific host pathways affected in SARS-CoV-2^+^ spots. We identified upregulation of several NFκB pathway components (NFKB2 and NFKBIA) and inflammatory cytokines (CXCL9, CCL17, and CCL21) in the presence of viral transcripts, in line with the abundant literature describing these molecules in the early steps of COVID-19 pathogenesis (Fig. 5a-b, Additional file 9: Table S9) [55–59]. Of note, we observed a localized downregulation of certain complement factors (C1QB, CFD, and C7) and interferon response genes (IFI6 and ISG15) (Fig. 5a, Additional file 9: Table S9), in contrast to their enrichment in COVID-19 lungs compared to control lungs in our samples and in others analyzed in previous works [33, 36, 51] (Additional file 7: Table S7). Furthermore, our results revealed an up- (SERPINE1 and PI15) or downregulation (A2M and WFDC2) of several protease inhibitor genes in SARS-CoV-2^+^ spots. Taken together, these results point to localized differences in the host response to the virus.

To explore the colocalization of the human host and SARS-CoV-2 RNA expression within distinct spatial tissue compartments and to potentially reveal cell type-specific responses to the presence of the virus, we identified unique DE genes between SARS-CoV-2^+^ and SARS-CoV-2^−^ spots within all ST and the five deconvolution-based clusters with the highest proportion of SARS-CoV-2 viral spots (Additional file 10: Table S10, Additional file 11: Table S11) (differential expression analysis used the Wilcoxon rank sum test and DESeq2 negative binomial distribution tests, *p*-value < 0.05). In the B-cell dominated deconvolution-based cluster, we observed the upregulation of several immunoglobulin variable genes (IGLV9-49, IGHV1-46), which, along with the enrichment of IGHV5-10–1 in the *en masse* DE analysis and of other variable genes (IGLV6-57, IGLV3-1, IGLV4-60) in a number of ST and deconvolution-based clusters, can signal the presence of virus-reactive B cell clones in the vicinity of infected cells. In addition, we identified the differential expression of several known SARS-CoV-2 entry factors between the SARS-CoV-2^+^ and SARS-CoV-2^−^ spots in several clusters, however, some of these were specific to a single cluster: we found an enrichment of MMRN1 in the SARS-CoV-2^+^ spots of the deconvolution-based endothelial cluster, while the same cluster showed downregulation of EMILIN1 and B cell/plasma cell-dominated spots showed upregulation of NRP1 in SARS-CoV-2^+^ B cell/plasma cell-dominated spots.

Beyond supporting previously described cellular mechanisms of COVID-19-related lung disease, our colocalization analysis revealed changes in the expressional levels of several novel genes, not yet discussed in this context (Fig. 5a, Additional file 9: Table S9). SRRM2 and HNRNPA2/B1 function as ubiquitous modulators of RNA processing [60, 61], while SMAP2, ARHGEF1, and TCIRG1 are leukocyte-enriched gene products implicated as regulators of intracellular vesicular trafficking and the autophagy pathway [62–64]. Based on in silico or in vitro analysis, several recent studies found evidence for a molecular interaction between SARS-CoV-2 viral components and SRRM2 [61, 65–67], HNRNPA2B1 [68–71], SMAP2 [72], and ARHGEF1 [72]. Furthermore, bulk RNA analysis identified the upregulation of TCIRG1 in SARS-CoV-2-infected lungs [31, 32]. Our colocalization analysis revealed that these genes showed a general upregulation in viral spots across all ST and most deconvolution-based clusters (B cell/plasma cell, endothelial, myeloid, and red blood cell-dominated), supporting their general relevance in the infected cells. The lack of enrichment of these transcripts in the SARS-CoV-2^+^ spots in the T/NK cell-dominated deconvolution-based cluster is likely due to these T/NK cells not being the primary target of the virus, but rather interacting with the SARS-CoV-2-infected cells as part of the adaptive immune response. Our in situ spatial analysis provides the first confirmation of the direct association of these expressional changes with defined areas actively expressing viral components in the COVID-19-diseased human lungs. In summary, by leveraging pathogen and host expression information for the same tissue section, we can explore highly localized cellular responses to the infection within distinct spatial compartments.

## Discussion

Exploratory methods to study host–pathogen interactions, especially approaches applicable to FFPE tissue blocks in which human tissue samples are routinely archived, are of major importance for understanding the pathogenesis of infectious diseases. While some of the previously published, spatially resolved transcriptomics methods [3–27, 73] are adept at working with such material, they are largely incompatible with providing a comprehensive view of both host and pathogen transcriptomic landscapes. The method proposed here enables insights into host response to pathogen infection within the spatial context of the tissue microenvironment at the whole transcriptome level, in an unbiased and high-throughput manner. Our results showcase the potential of this method through the dual capture of SARS-CoV-2 and human transcriptomes from lung tissues of COVID-19 patients and control donors. In addition, the extensive validation of our viral transcript capture with RNAscope, ISS, and qPCR demonstrates that our approach is highly reproducible, specific, and sensitive for the transcriptomes of interest.

Limitations of our proposed approach include the requirement of previous knowledge of the pathogen transcriptome of interest to develop targeted probes, the inability to detect different human RNA splice variants, the lack of capturing human non-coding RNA groups that may have important regulatory functions, and the inability to detect new viral variants since the viral RNA is not directly sequenced. However, probes targeting specific host RNA transcripts could be designed to overcome some of these shortcomings. Although the method requires the development of targeted probes for RNAs of interest, the general RNA-templated ligation probe design is already established by 10X Genomics for compatibility with the commercially available Visium Spatial Gene Expression for FFPE assay [74], enabling broad applicability. For example, future work could expand the approach by identifying regions with active viral replication with the development of probes targeting the negative strand of single-stranded positive-strand RNA viruses. In addition, although we observed SARS-CoV-2 gene colocalization in our tissue sections, where many significantly colocalized genes are located closer in proximity in the viral RNA, other SARS-CoV-2 gene pairs were not significantly colocalized in our tissues. The reasons behind these observed differences in SARS-CoV-2 gene colocalization remain to be further investigated. Viral dynamics and mechanisms over the course of infection, FFPE-induced degradation, and the detection limit of our probes (76% specificity and ~ 29% sensitivity of RNAScope) could contribute to the lack of statistically significant colocalization of some gene pairs. As the method is probe-based, aspects of viral biology (mutations, modifications, etc.), as well as degradation, could affect the ability of probes to bind and detect viral RNA. To detect a larger portion of viral RNA, our SARS-CoV-2 probe panel could be expanded to include more probes per gene and probes targeting sequences that facilitate the differentiation of gRNA and sgRNAs. A method combining probe-based capture with direct sequencing would capture degraded and fragmented viral RNA while differentiating viral strains and mutational patterns. Such enhancements to our method could elucidate additional insights into the spatial organization of SARS-CoV-2 infection.

In the context of SARS-CoV-2 infection, there is a large body of work available on exploring the transcriptomic changes occurring in the lungs of affected individuals, both on the single cell level [49, 51, 75–77] and spatially within intact tissue sections [30–35]. The latter approaches, however, largely rely on the detection of a low number of SARS-CoV-2 transcripts or immunolabeling of certain viral protein components and often require the preselection of regions of interest for the analysis. Our approach provides additional information on the relative level and distribution of viral transcripts by targeting 10 SARS-CoV-2 genes and the whole human transcriptome in the same tissue section to unbiasedly identify SARS-CoV-2^+^ and SARS-CoV-2^−^ tissue spots from the same dataset, without the need for any additional detection method. Future efforts could pursue establishing and validating techniques for the integration of different spatial datasets [78]. Such approaches could also benefit from our high-resolution, transcriptome-wide spatial co-detection of host and pathogen RNA that mitigates the technical challenges of collecting complementary information from consecutive tissue sections.

Our results largely agreed with previous works describing the distribution of different sgRNAs [45, 46] and the major lung cell types showing enrichment of viral components in the advanced disease phase [36], while also drawing attention to the potential interactions between SARS-CoV-2-infected cells and B, T, and NK cells, as part of the adaptive immune response to the infection. In addition, our methodology introduces a novel colocalization analysis between the pathogen and host transcriptomes, on the level of a tissue spot, close to single cell (1–10 cells/spot) resolution. Besides several known mediators of COVID-19 pathogenesis, our spatial analysis unveiled the upregulation of several genes (SRRM2, HNRNPA2/B1, SMAP2, ARHGEF1, and TCIRG1) likely involved in the intracellular steps of viral metabolism [31, 32, 61, 65–72], providing additional support for them as potential targets for future molecular interventions in COVID-19 disease [72, 79–81]. We also observed the upregulation of several immunoglobulin variable genes in certain subsets of SARS-CoV-2^+^ spots, potentially representing virus-associated B cell clones. With novel spatial transcriptomics technologies expanding into lymphocyte clone detection in the tissue context [82], such observations paired with antigen analysis could provide a basis for further research in the hope of identifying and generating neutralizing antibodies for clinical and scientific use.

Dual host–pathogen spatial transcriptome analysis has immense potential to provide unique information in a wide range of infectious diseases, specifically about entry factors, host-provided machinery for replication, productive and potentially harmful cellular countermeasures against the presence of the pathogen, and cell–cell interactions, in a tissue-specific manner and spatial context. In the case of recently emerged pathogens with substantial epidemiological relevance, such as SARS-CoV-1, DENV (Dengue virus), or ZIKV (Zika virus), or potential novel pathogens in the future, it would be advantageous to adopt similar spatial technologies at the forefront of related research efforts. At the same time, the high-resolution spatial co-detection of host and pathogen transcriptomes opens up the possibility of generating previously unavailable information even about otherwise well-described pathogens, making our method a promising tool for a wide range of studies in the field of infectious diseases.

## Conclusions

We demonstrate a proof-of-concept of deciphering host–pathogen interactions in FFPE sections through the colocalization of host and pathogen transcriptomes after their simultaneous capture in SARS-CoV-2-infected human lung tissue. The method has the potential to be applied to other human pathogens with the development of targeted probes and thus examine the interplay between host and pathogen across a multitude of human infectious diseases. Overall, our approach unleashes a potential new research line of studying infectious diseases in archived material at a large scale by exploring multiple transcriptomes in a single experiment.

## Methods

### Patient selection, sample collection, and processing

Collection of postmortem samples from lung tissue was performed at the 2^nd^ Department of Pathology, Semmelweis University (Budapest, Hungary) and the University Hospital Zurich (Switzerland). Autopsy cases were selected from patients who were hospitalized because of COVID-19 infection and died at the local clinical departments of the universities. Criteria for selection were: premortem positive (COVID-19 cases) or negative (control cases) SARS-CoV-2 PCR test, lack of malignancy of the lung, closed clinical documents, and less than 24 h as a postmortem interval (PMI). The autopsies were done in harmony with the World Health Organization’s (WHO) recommendation for the autopsy of COVID-19 cases [83]. The biopsies were fixated in formaldehyde (4%) and then went through a dehydration process overnight. Dehydrated samples were embedded into paraffin blocks and were stored at 4 °C until sectioning. The use of tissue specimens collected at Semmelweis University in this study was approved by the Hungarian Scientific Research Ethics Committee (ETT TUKEB IV/3961–2/2020/EKU). Samples and data were managed anonymously. At the University Hospital Zurich, small quantities of bodily substances removed in the course of an autopsy were anonymized for research purposes without consent, in the absence of a documented refusal of the deceased persons. In accordance with the Swiss Federal Act on Research involving Human Beings, this study did not require institutional board approval. Subsequent experiments were approved by the Swedish Ethical Review Authority (2010/313–31/3, 2018/689–32). Relevant clinical parameters of the patients included in this study are summarized in Additional file 3: Table S2.

### Sample selection—evaluating RNA Quality

Total RNA was extracted from each formalin-fixed paraffin-embedded (FFPE) sample block with the RNeasy FFPE kit (Qiagen, Cat. No. / ID: 73504) following the manufacturer's instructions (deparaffinization was performed using xylene (#28975.291 VWR) and 96% EtOH (#20823.290 VWR) or 100% EtOH (#1.00983.1000 VWR)). The concentration of extracted total RNA was determined with the RNA HS Qubit assay (Thermo Fisher Scientific) following the manufacturer's instructions. Total RNA was diluted to between 2-5 ng and RNA fragment length was assessed using the Agilent RNA 6000 Pico Kit following the manufacturer's instructions. The RNA quality of the sample was evaluated by the DV200 measurement (percentage of RNA fragments longer than 200 nucleotides) as specified in the Visium Spatial Gene Expression for FFPE – Tissue Preparation Guide [84]. Samples with a DV200 greater than 40% were selected for Visium FFPE, RNAScope, and in situ sequencing. Total RNA was used for RT-qPCR to determine the overall viral load in the samples (see “Viral load estimation by qPCR”).

### SARS-CoV-2 probe design

SARS-CoV-2 probes were designed as described [74], with probes designed based on the reference transcriptome Wuhan-Hu-1 isolate Sars_cov_2.ASM985889v3, Ensembl build 101 (https://covid-19.ensembl.org/Sars_cov_2/Info/Index) and were not designed for other strains or mutation patterns. Probes were designed to target the SARS-CoV-2 genes Surface glycoprotein (S), Envelope protein (E), Membrane glycoprotein (M), ORF1ab, ORF3a, ORF7a, ORF7b, ORF8, Nucleocapsid phosphoprotein (N), and ORF10 (Additional file 2: Table S1, Additional file 12: Table S12, Additional file 13: Table S13) (10X Genomics).

### Spatial Transcriptomics

Consecutive 5 µm tissue sections from each sample were placed onto Visium Spatial Gene Expression slides (PN: 2000233, 10X Genomics) and stored overnight in a desiccator [84]. 5 µm sections consecutive to the ones used for Visium FFPE were placed onto Superfrost Plus microscope slides (#631–9483, VWR) and stored at 4^◦^C until used for RNAScope and in situ sequencing. Deparaffinization, Hematoxylin and Eosin staining, and decrosslinking were performed as specified in the Visium Spatial Gene Expression for FFPE – Deparaffinization, H&E Staining, Imaging & Decrosslinking Demonstrated Protocol [85]. Spatial gene expression profiling of RNA from FFPE lung samples was performed by following all steps in the Visium Spatial Gene Expression Reagent Kits for FFPE User Guide [42] with the modifications: for COVID-19 samples (Additional file 3: Table S2), four 5 µm consecutive sections per patient sample tissue FFPE block were placed on Visium Spatial Gene Expression slides (PN: 2000233, 10X Genomics). For step 1.1.g, Human whole transcriptome (WT) probes (10X Genomics) were added to two consecutive sections (technical replicates) with the Probe Hybridization Mix: 19*.*8 µL Nuclease-free water, 77*.*0 µL FFPE Hyb Buffer, 6*.*6 µL LHS Human WT probes, and 6*.*6 µL RHS Human WT probes, per sample. Human WT and spike-in custom probes targeting SARS-CoV-2 genes (10X Genomics) were added to the remaining two consecutive sections (technical replicates) with the Probe Hybridization Mix: 14*.*5 µL Nuclease Free water, 77*.*0 µL FFPE Hyb Buffer, 6*.*6 µL LHS Human WT probes, and 6*.*6 µL RHS Human WT probes, 2*.*6 µL LHS viral probes, and 2*.*6 µL RHS viral probes, per sample. For control patient samples, two consecutive sections (technical replicates) were processed as described for the COVID-19 samples, with adding Human WT and SARS-CoV-2 spike-in probes to all sections. For step 4.1.d, qPCR (Bio-Rad) step 4 was run for a total of 30 cycles. For step 4.2.d, the Sample Index PCR was performed with 15 cycles for 1C, 15–16 cycles for 3C, 18–19 cycles for 2C, 16 cycles for 4nC, and 18 cycles for 5nC. After step 4.4, the concentration of sequence libraries was determined with 2 µL of each sample run with the dsDNA HS Qubit assay (Thermo Fisher Scientific).

### Spatial Transcriptomics hematoxylin & eosin imaging

Hematoxylin & Eosin brightfield images were acquired with a Zeiss Axiolmager.Z2 VSlide Microscope using the Metasystems VSlide scanning system with Metafer 5 v3.14.179 and VSlide software. The microscope has an upright architecture, uses a widefield system, and a 20X air objective with a numerical aperture (NA) of 0.80 was used. The camera was a CoolCube 4 m with a Scientific CMOS (complementary metal–oxide–semiconductor) architecture and monochrome with a 3.45 × 3.45 µm pixel size. All brightfield images were taken with a Camera Gain of 1.0 and an Integration Time/Exposure time of 0.00011 s.

### Spatial Transcriptomics sequencing

Sequencing libraries were pooled and diluted with Elution Buffer (EB) to a final concentration of 10 nM, using a target sequencing depth of 50,000 mean read pairs/spot to determine the dilution for each sample [42]. After sample pooling, pooled library concentrations were checked with qPCR (Bio-Rad) before loading into the sequencer. Libraries were sequenced on an Illumina NovaSeq 6000 with paired-end, dual-indexed sequencing run type, and parameters following those specified in the Visium Spatial Gene Expression Reagent Kits for FFPE User Guide sequencing instructions [42] [R1: 28 cycles, R2S: 50–52 cycles], with a spike-in of PhiX at 1% concentration, except one sample, 3C, was run with R2S: 75 cycles.

### In situ sequencing (ISS)

Optimal RNA integrity and assay conditions were assessed using *MALAT1* and *RPLP0* housekeeping genes only using the HS Library Preparation kit for CARTANA technology (part of 10X Genomics) and following manufacturer’s instructions on 5 µm tissue sections from representative sample 1C. Since the control probes test showed positive and expected results, in situ sequencing was then performed on two 5 µm consecutive sections from sample 1C and one consecutive section from each control sample (4nC and 5nC). Superfrost Plus microscope slides (#631–9483, VWR) containing 5 µm tissue sections were stored at 4℃ until processing. FFPE sections were baked for 1 h at 60°C to partially melt paraffin and increase tissue adherence. Next, sections were deparaffinized using xylene for 2 × 7 min followed by an EtOH gradient to remove xylene and rehydrate the sections. Sections were then permeabilized using citrate buffer pH 6.0 (C9999 Sigma Aldrich) for 45 min at 95°C. For library preparation, chimeric padlock probes (targeting directly RNA and containing an anchor sequence as well as a gene-specific barcode) for a custom panel of SARS-CoV-2 S and E genes were hybridized overnight at 37°C, then ligated before the rolling circle amplification was performed overnight at 30°C using the HS Library Preparation kit from CARTANA technology and following the manufacturer’s instructions. All incubations were performed in SecureSeal™ chambers (Grace Biolabs). For tissue section mounting, Slow Fade Antifade Mountant (Thermo Fisher) was used for optimal handling and imaging. Quality control of the library preparation was performed by applying anchor probes to simultaneously detect all rolling circle amplification products from all genes in all panels. Anchor probes are labeled probes with Cy5 fluorophore (excitation at 650 nm and emission at 670 nm). All samples passed the quality control l and were sent to CARTANA (part of 10X Genomics), Sweden, for a single cycle in situ barcode sequencing, imaging, and data processing. Briefly, adapter probes and a sequencing pool (containing 4 different fluorescent labels: Alexa Fluor® 488, Cy3, Cy5, and Alexa Fluor® 750) were hybridized to the in situ libraries to detect SARS-CoV-2 gene-specific barcodes. This was followed by multicolor epifluorescence microscopy, scanning the whole area and thickness of the tissues. Raw data consisting of 20 × magnification images from 5 fluorescent channels (DAPI, Alexa Fluor® 488, Cy3, Cy5, and Alexa Fluor® 750) and individual z-stacks, were flattened to 2D using maximum intensity projection with a Nikon Ti2 Nikon Ti2 (software NIS elements) utilizing Zyla 4.2 camera. After image processing, which includes image stitching, background filtering, and a sub-pixel object registration algorithm, true signals were scored based on signal intensities from individual multicolor images. The results were summarized in a CSV file and gene plots were generated using MATLAB.

### RNAScope assay and imaging

RNAScope assay was performed on lung 5 µm FFPE sections on Superfrost Plus microscope slides (#631–9483, VWR) cut from depths consecutive to the sections mounted on Visium slides. The slides were baked in a dry oven for 1 h at 60°C and then deparaffinized in xylene (2 × 5 min) and absolute ethanol (2 × 1 min) at room temperature. After drying, the sections were incubated in RNAScope Hydrogen Peroxide for 10 min at room temperature, followed by washing steps (2x) in distilled water. Target retrieval was performed using a 1 × RNAScope Target Retrieval Reagent for 15 min, at a temperature constantly kept above 99°C in a hot steamer. The slides were then rinsed in distilled water, incubated in absolute ethanol for 3 min, and dried at 60°C. After creating a hydrophobic barrier, the slides were left to dry overnight. On the second day, the sections were incubated in RNAScope Protease Plus solution for 30 min at 40°C, followed by washing in distilled water. RNAScope V-nCov2019-S probe, RNAScope Positive Control probe (Hs-PPIB), and RNAScope Negative Control Probe (DapB) were hybridized to separate sections for 2 h at 40°C, then the slides were washed twice for 2 min in 1 × Wash Buffer. The probe-specific signal was developed with an RNAScope 2.5 HD Detection Reagent – RED kit. Sequential hybridization of amplification reagents AMP1-4 happened at 40°C for 30–15-30–15 min, while AMP5 and AMP6 were applied at room temperature for 1 h and 15 min, respectively, with two washing steps in 1 × Wash Buffer after each incubation period. For signal detection, each section was incubated for 10 min at room temperature in a 120 ul RED Working Solution, consisting of Fast RED-B and Fast RED-A reagents in a 1:60 ratio. All the protease digestion, probe hybridization, signal amplification, and signal detection steps were performed in a HybEZ Humidity Control Tray, which were either placed into a HybEZ Oven for the 40°C incubation steps or kept at room temperature. Following two washing steps in tap water, the slides were counterstained with 50% Gill’s Hematoxylin staining solution for 2 min at room temperature, thoroughly rinsed with tap water, then soaked in 0.02% Ammonia water bluing solution, and finally washed again in tap water. The slides were then dried completely at 60°C and then quickly dipped into xylene before mounting them with VectaMount Permanent Mounting Medium. The RNAScope signal was imaged and evaluated with a Leica DM5500B microscope with an HC PL APO 20x/0.70 DRY objective, using Extended Depth of Field (EDoF) imaging in the Leica Application Suite X (LAS X) software platform.

### Spatial Transcriptomics—data processing

#### Count matrices generation

The gene expression matrices were generated by space ranger (version 1.3.0) ‘count’ (standard settings set except *–no-bam*). The transcriptome reference was custom-made from space ranger ‘mkref’ using the Human reference dataset (GRCh38 Reference—2020-A), and SARS-CoV-2 genome assembly (ASM985889v3). The Human Probe Set from 10X Genomics (Visium Human Transcriptome Probe Set v1.0) with 10X Genomics custom probes for SARS-CoV-2 probes appended to it, was used as the probe set reference in space ranger ‘count’.

#### Quality control

The filtered count matrices (filtered_feature_bc_matrix.h5), and tissue images from space ranger output were analyzed in R (version 4.1.1) using the Load10X_Spatial function available in Seurat (version 4.0.4) [86]. The filtered count matrices were separated into human count data and SARS-CoV-2 count data matrices. Spot level filtering was performed on the human count matrices to keep spots with at least 400 genes, and 500 UMIs. An additional spot filter was applied where a novelty score for each spot was calculated by taking the log transform of the ratio of the total genes detected divided by the total UMI counts detected in the same spot (Formula: log10(nFeature_RNA) / log10(nCount_RNA)). Spots with a score greater than 0.87 were kept. Gene level filtering was applied to omit genes that did not appear in at least 1 spot. These count matrices were also filtered for Hemoglobin gene counts (Additional file 2: Table S1). SARS-CoV-2 count matrices were normalized by dividing the SARS-CoV-2 gene UMI counts by the number of probes used to target the respective gene. 1 SARS-CoV-2 UMI was detected from two different sections, one control, and one COVID-19, that did not have SARS-CoV-2 probes added and was considered as a background signal.

#### Clustering analysis

The Seurat SCTransform function was applied to normalize the individual filtered count matrices, and integrated in Seurat using SelectIntegrationFeatures, and IntegrateData. Principal Component Analysis (PCA), and UMAP were applied using 50 principal components, and 35 were further used in downstream analysis and clustering. Batch effects were addressed, and removed using RunHarmony (version 0.1.0; group.by.vars as slide ID, and 25 iterations) applied on the PCA-computed matrix [87]. Clustering was applied at a resolution of 0.4.

#### Differential gene expression

Differentially expressed (DE) genes were found using ‘FindMarkers' in Seurat, with default settings on the SCT normalized matrix, except min.cells.group set to 2 to include at least 2 spots from each group. Both the Wilcoxon rank sum test and “bimod” Likelihood-ratio tests were used, with both tests yielding the same results. Both upregulated and downregulated DE genes were identified, with an adjusted *p*-value of 0.005**.** Celltype-specific annotation of the DE genes was performed manually, by using the Human Single Cell Atlas [88], PanglaoDB [89], and recently published single-cell transcriptomic data of the human lung [36, 51] as main resources.

#### Colocalization analysis

For the colocalization analysis, a direct spot-level comparison within the COVID-19 sections was performed. The DE genes distinguishing SARS-CoV-2^+^ spots from SARS-CoV-2^−^ spots were obtained as described in the Methods section "Differential Gene Expression" with an additional filter of average logFC ± 1.0. As an extension to the colocalization analysis, DE genes between SARS-CoV-2^+^ and SARS-CoV2^−^ spots were also identified for each cluster as well as for selected cell type-dominant spots that were determined based on the deconvolution analysis. Differential expression analysis for DE genes distinguishing SARS-CoV-2^+^ spots from SARS-CoV-2^−^ spots were found using the Wilcoxon rank sum test and DESeq2 negative binomial distribution tests (*p*-value < 0.05), with both tests giving the same results.

#### Deconvolution analysis

Spot deconvolution of the ST data was performed using Stereoscope (v0.3) [90] and single-cell data set SCP1052 [36] downloaded from the Single Cell portal (https://singlecell.broadinstitute.org/single_cell/study/SCP1052/covid-19-lung-autopsy-samples#study-summary). Given that the patient samples included in our study were of a later COVID-19 disease stage with 13–17 days from COVID-19 diagnosis to death (Additional file 3: Table S2), we selected patients from the single cell study that fell within a similar timeframe (13–30 days from symptom onset to death) as our samples. The R scripts used to prepare the input data for the deconvolution and generate the summary of the results are presented under the deconvolution folder (https://github.com/giacomellolab/DualST_Study/tree/main/R_scripts/deconvolution) in our GitHub repository DualST_Study (https://github.com/giacomellolab/DualST_Study) [91].

#### SARS-CoV-2 gene colocalization

Metrics for the colocalization of the different SARS-CoV-2 genes were calculated from the dataset containing the SARS-CoV-2 signal information. The SARS-CoV-2 genes count matrix was used to calculate, for each SARS-CoV-2 gene, the total number of spots the gene was detected in.

To determine the significantly colocalized SARS-CoV-2 gene pairs across the ST spots, we implemented an approach similar to a recent publication that calculated the probability of the co-occurrence of different cell type pairs in RNAseq data [92]. Rather than cell type pairs, we constructed matrices for SARS-CoV-2 gene pairs (present/absent × present/absent) across the SARS-CoV-2^+^ spots (matrices contained the number of spots with both genes detected, number of spots with only gene 1 detected, number of spots with only gene 2 detected, and number of SARS-CoV-2^+^ spots without either gene detected) and calculated the *p*-value for each gene pair using both the Chi-Square test for independence (chisq.test function in R), the Fisher’s exact test (fisher.test function in R) (to account for some frequencies being higher and some lower), and a permutation test of independence for a two-way contingency table (each SARS-CoV-2 gene pair matrix) based on Monte Carlo resampling (10,000 resamples) with a Approximative (Monte Carlo) Pearson chi-squared test using the coin R package [93].

### Elastic registration workflow

Lung tissue sections of various assays (ST, RNAScope, ISS) from sample 1C were registered elastically using conformal mapping. To illustrate, we used the registration between the ST section and RNAScope section as an instance, and the registration between the ST section and ISS section was done in a similar way. Firstly, four corner points were manually selected on each tissue section respectively, then each section was conformally mapped onto a square with the corners chosen mapped to the square vertices. Since the sections of various assays have been mapped onto the same square, one-to-one correspondence among ST and RNAScope sections can be easily established. The positions of aligned RNAScope section pixels can be transferred from the position of the corresponding pixels in the ST section via the aforementioned one-to-one correspondence. Hence, the elastic registration was achieved by displaying the RNAScope section pixel colors in the positions of ST section pixels.

### Validation by RNAScope

RNAScope and ST images from all samples were manually aligned with Adobe Photoshop 2022. The RNAScope chromogenic detection of the S gene with FastRed was used to distinguish the RNAScope signal from lung pigmentation and tar deposits. All dots of chromogenic red signal were considered as positive SARS-CoV-2 S gene signal, since the majority of the signal was above 1 dot per 10 nuclei area, in line with how others assessed RNAScope signal in SARS-CoV-2 viral low samples [30, 36, 94]. RNAScope was considered the gold standard for comparison to the ST signal. The number of ST spots where the SARS-CoV-2 S gene was detected, and where the RNAScope S gene signal was also obtained, was calculated. To adjust for the use of consecutive sections for ST and RNAScope experiments, the agreement of ST and RNAScope in 200 × 200 µm^2^ block areas was evaluated. Since a manual annotation of sample 1C was in close agreement with the computational approach, the computational approach to calculate the specificity of the SARS-CoV-2 S gene detection by ST was used.

The computational validation was performed as follows: the RNAScope signal was detected with an ad hoc Matlab (version R2021b) algorithm, which is specified in the next section “Automatic detection of RNAScope signal”; then both the binary ST and RNAScope signal images were aligned and binned into 200 × 200 µm^2^ blocks (Additional file 1: Fig. S3-S4). Each block in an RNAScope/ST signal image was regarded as an observation (those blocks that contain no tissue area were regarded as no observation and were excluded from any further analysis and counting). The specificity of our method to capture the SARS-CoV-2 expression was calculated by considering the RNAScope approach as the ground truth and as follows:

$$Specificity = \frac{\#TN}{\#TN + \#FP}$$

Where the number (#) of True Negatives (TN) was defined as the number of blocks containing neither RNAScope nor ST signals and the number of False Positives (FP) as the number of blocks containing only ST but no RNAScope signals.

For signal quantification in the sample with the highest viral load (1C), ST, RNAScope, and ISS images were elastically registered using conformal mapping (see “Elastic registration workflow” above). The RNAScope signal was detected as described below under “Automatic detection of RNAScope signal”, and quantified as the number of signal pixels per area. The signal was normalized by dividing by 80 to place signals on a similar scale to ST. Both the ST and RNAScope signal images were aligned and binned into circular disks of 300 µm diameter, the positions of which were chosen by merging every seven neighboring ST spots (one central spot plus its six neighboring spots) into a larger circular disk area. The distance between any two neighboring spot centers is 100 µm, and the circular disk diameter of 300 µm is just triple that distance. Each disk in an RNAScope/ST signal image was regarded as an observation (those disks that contain no tissue area were regarded as no observation and were excluded from any further analysis and counting). The specificity of our method to capture the SARS-CoV-2 expression was calculated by considering the RNAScope approach as the ground truth and as follows:

$$\begin{array}{c}Specificity = \frac{\#TN}{\#TN + \#FP}\\\\ Sensitivity = \frac{\#TP}{\#TP + \#FN}\end{array}$$

Where the number (#) of True Negatives (TN) was defined as the number of disks containing neither RNAScope nor ST signals, the number of False Positives (FP) as the number of disks containing only ST but no RNAScope signals, the number of True Positives (TP) as the number of disks containing both ST and RNAScope signals, and the number of False Negatives (FN) as the number of disks containing only RNAScope but no ST signals. A Chi-squared test of independence was performed on the resulting confusion matrix using the chisq.test function in R, based on the approach taken by a recent study that compared the degree of agreement between virtual staining and immunohistochemistry staining methods [95].

### Automatic detection of RNAScope signal

RNAScope signals were detected with a chromatical analytic method. First, the original RGB image was transformed into the Hue-Saturation-Value (HSV) format, where the bright regions in the hue channel correspond to the RNAScope signals in the original histological image. The brightest regions became the foreground by thresholding the hue value of the image. Morphological post-processing steps were performed to refine the shape of the signal regions, the details of which are available in the code (see “Availability of data and materials”). The pixels whose hue was over 0.85, saturation over 0.25, and value over 0.40 were recognized as signal candidates. After performing a morphological opening operation, the collection of signal candidates was output as final RNAScope signals. Additional file 1: Fig. S5 displays the original tissue subimage, the hue channel, and the RNAScope signal subimage after the thresholding.

### Validation by ISS

ISS consecutive section images and ST images for sample 1C were manually aligned with Adobe Photoshop 2022. Due to the use of non-consecutive sections, there was ~ 300 µm in between the ST and ISS sections. The agreement between E and S gene signals for ST and ISS in block areas of 200 × 200 µm^2^ was evaluated using the same computational approach as used for the RNAScope validation. The quantification of the S gene signal in 300 µm^2^ disk areas was evaluated using the same approach as that for RNAScope validation.

### Viral load estimation by qPCR

The relative amount of SARS-CoV-2 RNA present in the tissue-extracted RNA was determined by qRT-PCR targeting the E-gene of the virus using the Takara PrimeDirect probe, RT-qPCR mix (Takara Bio Inc, Japan). qRT-PCR was performed in a 25 uL reaction volume, with 175 ng input RNA, 400 nM each primers (Eurofins, USA; Forward: 5’-ACAGGTACGTTAATAGTTAATAGCGT-3’ and  reverse: 5’-ATATTGCAGCAGTACGCACACA-3’),  200 nM probes (Sigma-Aldrich, UK; 5’- [FAM]-ACACTAGCCATCCTTACTGCGCTTCG-[BBQ650]) and cycling conditions of  initial denaturation 90℃ for 3 min, reverse transcription 60℃ for 5 min, followed by 45 cycles of 95°C for 5 sec, 58°C for 30 sec (PMID: 34006825**)**. The human RNase P gene was used as an internal control in the PCR to validate the quality of the extracted RNA. The relative quantification of viral copies per ng of input RNA was performed by comparing the results to the known viral copies/reaction (Lowest detectable: 750 copies/reaction) of the All-WHO-CDC-Genes-nCoV-Control-Plasmid (Eurofins Genomics, #5004ALL001).

### Supplementary Information

**Additional file 1: Fig. S1 - S17. Table S3.** Confusion matrix comparing RNAScope and ST SARS-CoV-2 S gene signal. The confusion matrix is generated from 300 µm disk areas used in the quantitative validation analysis that determines the SARS-CoV-2 S gene signal in each area for RNAScope and our ST approach.

**Additional file 2: Table S1.** Spatial transcriptomics genes. The human (16,688) and SARS-CoV-2 (10) gene transcripts targeted in the study.

**Additional file 3: Table S2.** Patient data. Relevant clinical parameters of the 5 patients included in this study are summarized.

**Additional file 4: Table S4.** Spatial transcriptomics sample section summary. Sequence library information for each of the 13 patient tissue sections assayed with spatial transcriptomics (ST).

**Additional file 5: Table S5.** Clustering differentially expressed genes. Differentially expressed (DE) marker genes for each spatial transcriptomic (ST) cluster. Differential expression analysis to identify cluster specific DE genes used Wilcoxon rank sum test and “bimod” Likelihood-ratio tests, *p-*value < 0.05.

**Additional file 6: Table S6.** Cluster 5 subclustered differentially expressed genes. Differentially expressed (DE) genes for each subcluster of cluster 5. Differential expression analysis used Wilcoxon rank sum test and “bimod” Likelihood-ratio tests, *p*-value < 0.05.

**Additional file 7: Table S7.** Differentially expressed genes in COVID-19 vs. control sections. Differentially expressed (DE) genes for COVID-19 versus control lung tissue samples. Differential expression analysis used Wilcoxon rank sum test and “bimod” Likelihood-ratio tests, *p*-value < 0.05.

**Additional file 8: Table S8.** Differentially expressed genes in COVID-19 vs. control per cluster. DE genes per cluster for COVID-19 versus control lung tissue samples. Differential expression analysis used Wilcoxon rank sum test and “bimod” Likelihood-ratio tests, *p*-value < 0.05.

**Additional file 9: Table S9.** Colocalization analysis results of COVID-19 sections. Differentially expressed genes in SARS-CoV-2^+^ vs. SARS-CoV-2^- ^spots in COVID-19 sections. Differential expression analysis between SARS-CoV-2^+^ and SARS-CoV-2^-^ spots in COVID-19 sections overall used Wilcoxon rank sum test and DESeq2 negative binomial distribution tests, *p*-value < 0.05.

**Additional file 10: Table S10.** Colocalization analysis results of COVID-19 sections within ST clusters. Differentially expressed genes in SARS-CoV-2^+^ vs. SARS-CoV-2^-^ spots within ST clusters across COVID-19 sections. Differential expression analysis between SARS-CoV-2^+^ and SARS-CoV-2^-^ spots within ST clusters across COVID-19 sections overall used Wilcoxon rank sum test and DESeq2 negative binomial distribution tests, *p*-value < 0.05.

**Additional file 11: Table S11.** Colocalization analysis results of COVID-19 sections within cell types. Differentially expressed genes in SARS-CoV-2^+^ vs. SARS-CoV-2^- ^spots within cell types across COVID-19 sections. Differential expression analysis between SARS-CoV-2^+^ and SARS-CoV-2^-^ spots within cell type clusters across COVID-19 sections overall used Wilcoxon rank sum test and DESeq2 negative binomial distribution tests, *p*-value < 0.05.

**Additional file 12: Table S12.** SARS-CoV-2 ST gene probe sequences. Probe sequence information for the ST SARS-CoV-2 gene probes used in the study, including the GC content and melting temperature (Tm).

**Additional file 13: Table S13.** ST SARS-CoV-2 ordered probe sequences. Ordered SARS-CoV-2 probe sequences for the ST SARS-CoV-2 gene probes used in the study.

**Additional file 14.** Review history.

## Data Availability

Raw sequence data for all samples included in this study are available on the European Genome-Phenome Archive (EGA) under controlled access due to reasons of sensitivity and can be requested from the Dataset Access Committee (DAC). The accession IDs for the study and DAC are EGAS00001006186 [96] and EGAC00001002635 [97] respectively. Processed gene count matrices, related metadata, corresponding ST tissue microscopy images, and RNAScope images used in the Validation analysis are available on Mendeley Data [98]. ISS images for SARS-CoV-2 S and E genes are also available on Mendeley Data [99]. RNAscope images for the SARS-CoV-2 S gene for each sample are available on FigShare [100–104] and the deconvolution results as an R object are available on FigShare [105], all under FigShare project ID 134597. All RNAScope images for the SARS-CoV-2 S gene probe, positive control probe, and negative control probe are also available on Zenodo at 10.5281/zenodo.8039011 [106]. Scripts to generate the count matrices and all related R scripts used in the clustering, differential expression, colocalization analysis, and the program for the computational validation can be accessed from our GitHub repository, DualST_Study (https://github.com/giacomellolab/DualST_Study) [91]. All versions of scripts and source code used in the manuscript are additionally deposited on Zenodo at 10.5281/zenodo.8314652 [107]. All source code is compliant with the Open Source Initiative under the MIT license.
