# Supplementary material for: Dual spatially resolved transcriptomics for human host–pathogen colocalization studies in FFPE tissue sections
Source: Genome Biol. 2023 Oct 19;24:237. doi: 10.1186/s13059-023-03080-y (PMC10588020; doi:10.1186/s13059-023-03080-y)
Supplement: Supplementary file 1 — Additional file 1: Fig. S1 - S17. Table S3. Confusion matrix comparing RNAScope and ST SARS-CoV-2 S gene signal. The confusion matrix is generated from 300 µm disk areas used in the quantitative validation analysis that determines the SARS-CoV-2 S gene signal in each area for RNAScope and our ST approach. [file 13059_2023_3080_MOESM1_ESM.pdf]

**Supplementary Information**

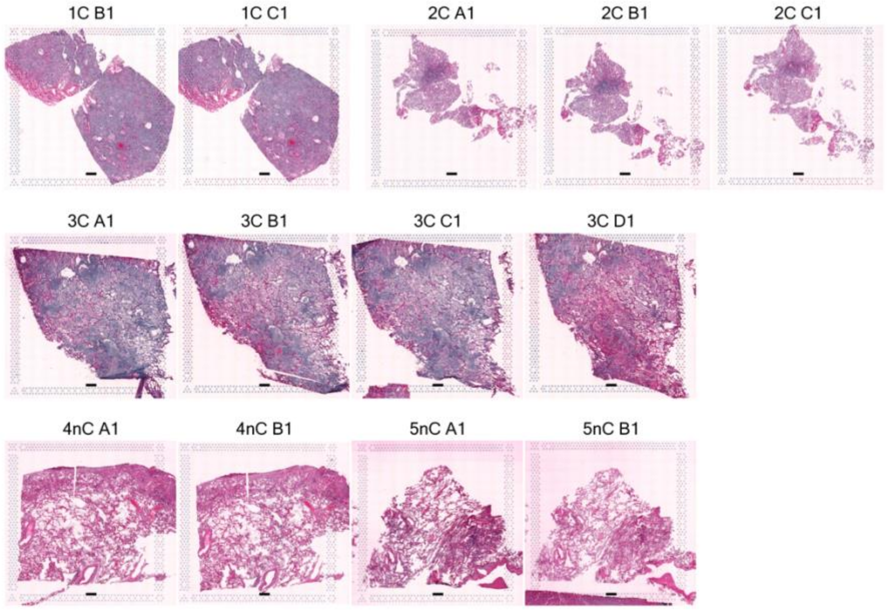


**Figure S1. Study sample sections.** The 13 tissue sections across 5 patient lung samples, 3 COVID-19 samples (1C, 2C, 3C) and 2 control samples (4nC, 5nC), used for ST in this study. Scale bars are 500µm.

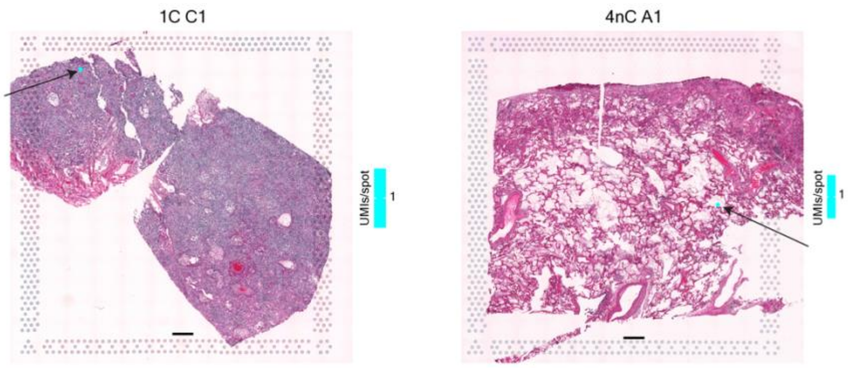


**Figure S2. Background SARS-CoV-2 detection.** 1 UMI count across all assayed SARS-CoV-2 genes was detected in two tissue sections, a COVID-19 section with no SARS-Cov-2 probes added (1C C1) and a control section (4nC A1). Scale bars are 500µm.

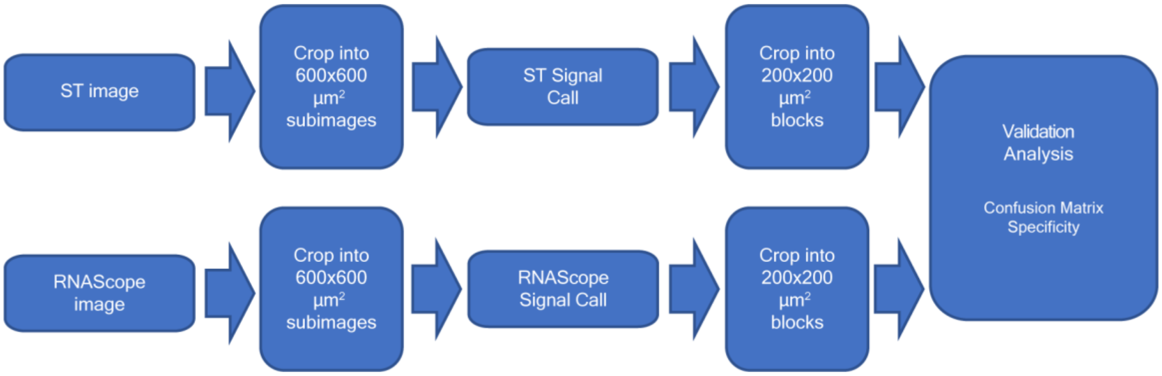

**Figure S3. RNAScope Validation Workflow.** The RNAScope validation workflow consists of two independent and similar procedures, which process the RNAScope or ST image signals respectively, and compare the two procedure outputs. In each RNAScope/ST procedure, the original histological image is cropped into median sized subimages. The RNAScope/ST signals are detected by the corresponding method. Afterwards, the signal images are cropped into small blocks, which are utilized for the validation analysis.

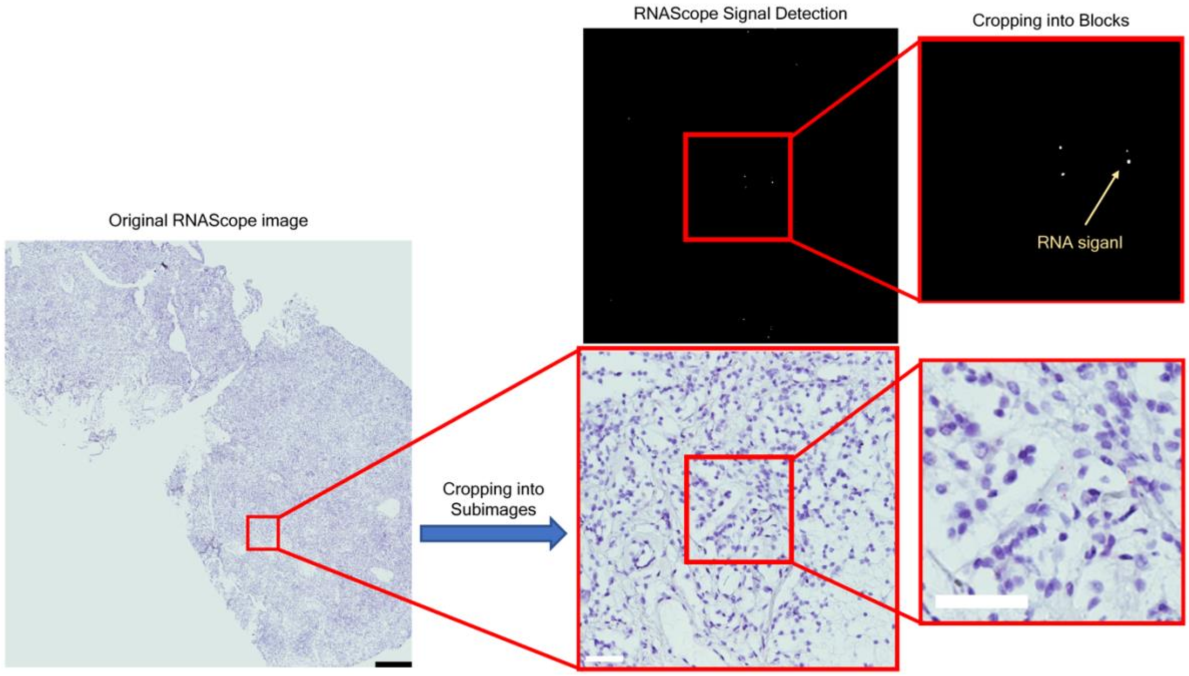


**Figure S4. Workflow of RNAScope light-field image processing.** A visualization of the RNAScope signal processing procedure, where the RNAScope signal images are the same size as the corresponding light field images. The ST signal processing shares essentially the same procedure, except for the RNAScope/ST signal call. The black scale bar represents the length of 500µm and the white bars represent the length of 50µm.

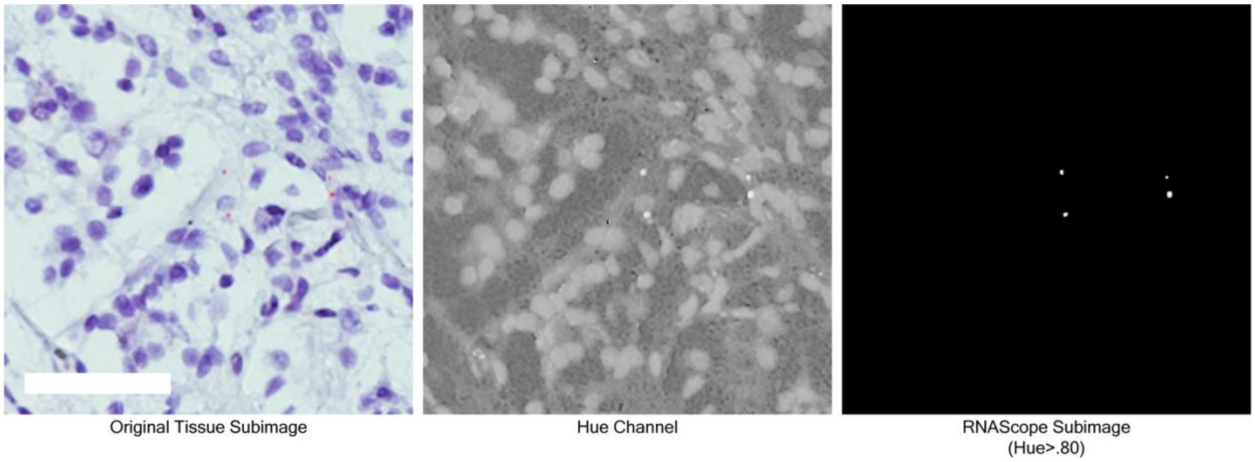


**Figure S5**. **RNAScope Signal Call.** The original tissue subimage, the hue channel, and the RNAScope signal subimage after the thresholding, where the white scale bar represents the length of 50µm, and all the images are of the same size and resolution.

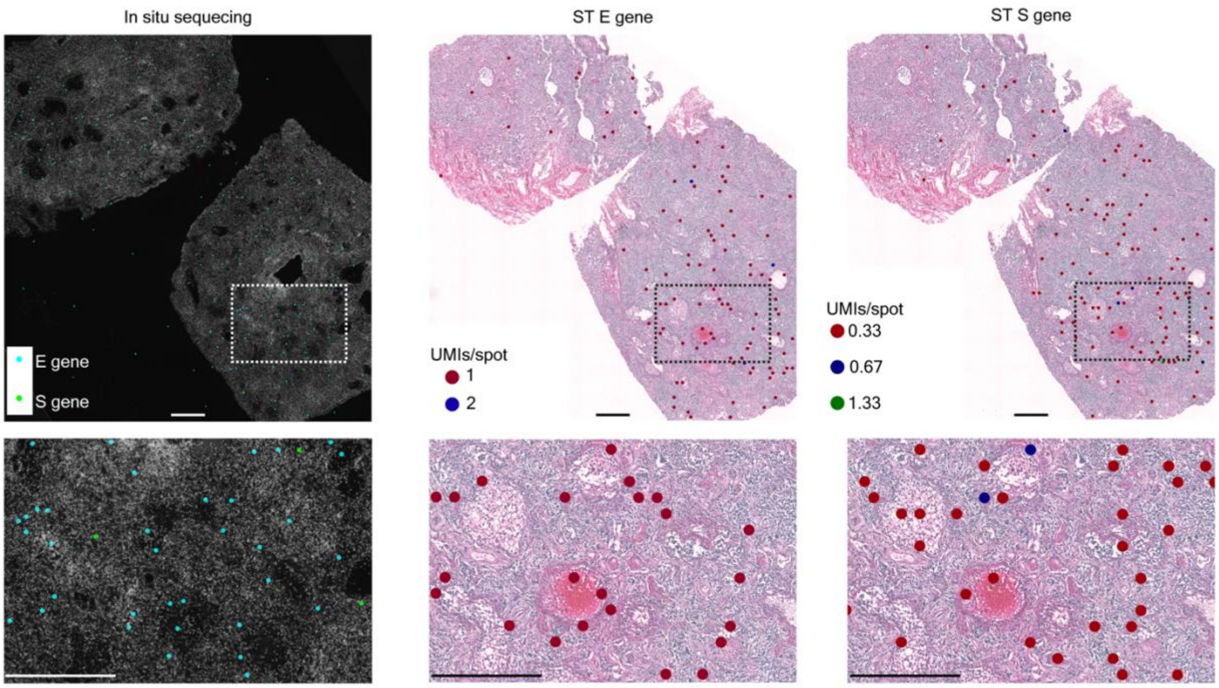

**Figure S6. *In situ* sequencing validation of ST S & E gene signal.** Enlargement of Figure 1c showing the *in situ* sequencing (ISS) signal and ST signal for genes S and E (~300µm between ISS and ST sections). Scale bars are 550µm.

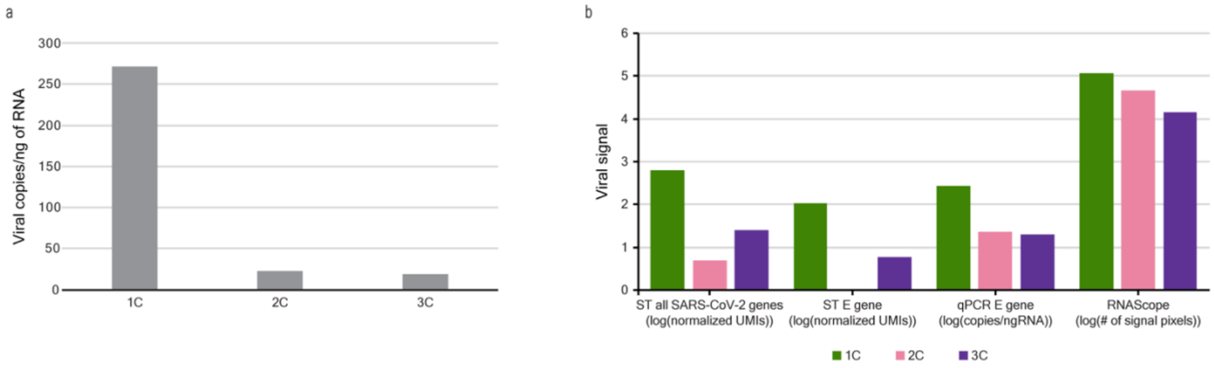


**Figure S7. Viral signal across RNA detection techniques.** (a) Viral copies per ng of input RNA across each COVID-19 sample section detected by qPCR. (b) Viral signal across all SARS-CoV-2 genes detected by ST, E gene detected by ST E gene detected by qPCR, and S gene detected by RNAScope.

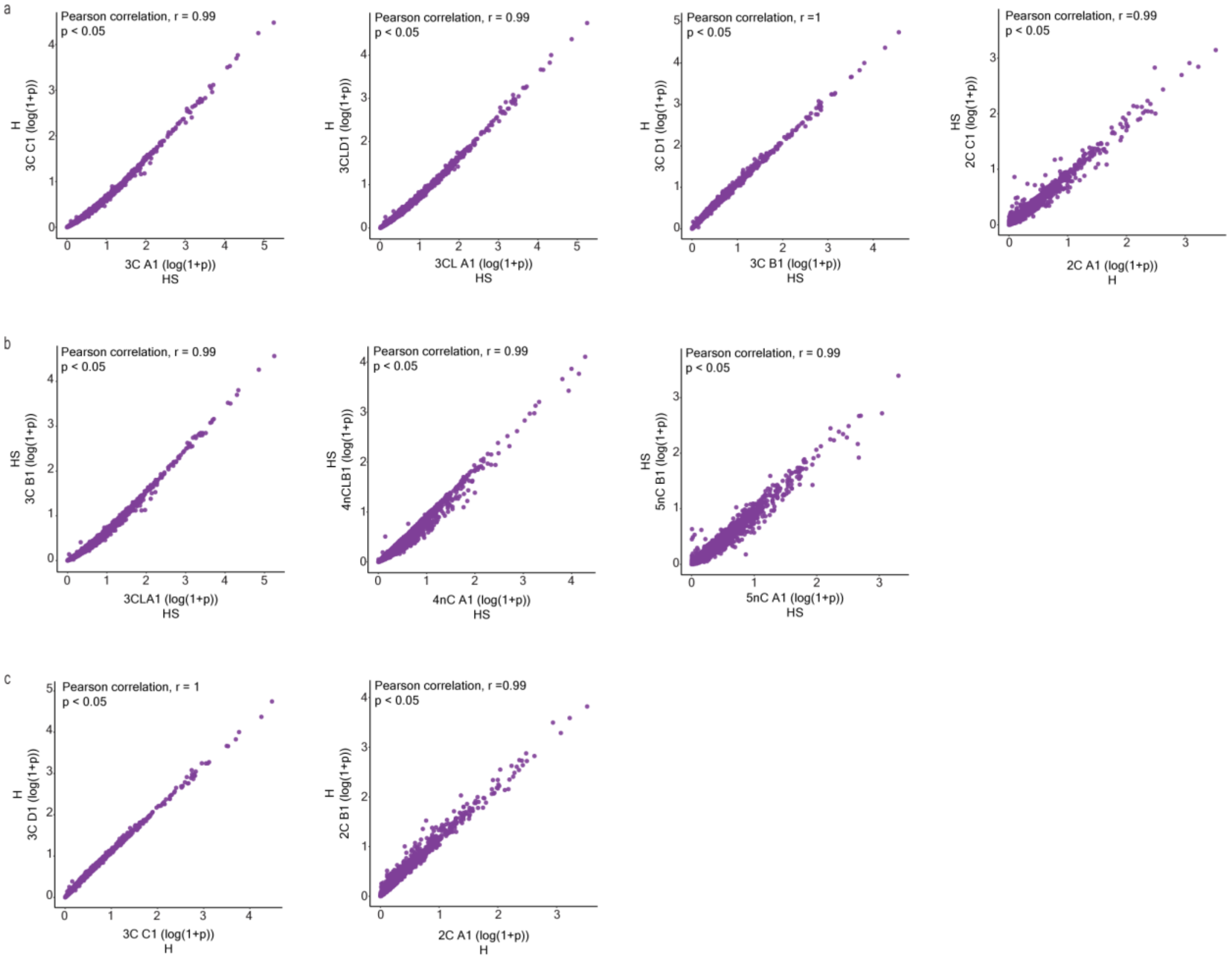


**Figure S8**. **Highly reproducible capture of human transcriptome data.** Pearson correlation of average human gene expression between consecutive sections for each sample (n=5 samples), with either (a) one section with human and SARS-CoV-2 probes added (HS) and the other with only human probes added (H), (b) both sections with human and SARS-CoV-2 probes added (HS), or both sections with only human probes added (H), p-value < 0.05.

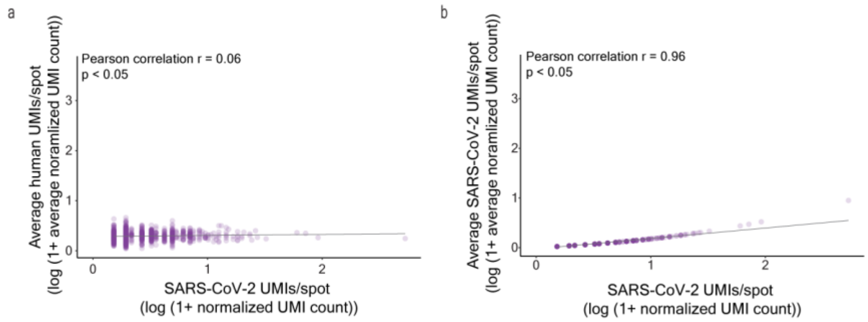


**Figure S9. Correlation of SARS-CoV-2 gene expression levels (UMIs) to the average UMI counts per spot.** (a) Pearson correlation between the SARS-CoV-2 UMI counts per spot and the average human UMI counts per spot, p-value < 0.05. (b) Pearson correlation between the SARS-CoV-2 UMI counts per spot and the average SARS-CoV-2 UMI counts per spot, p-value < 0.05.

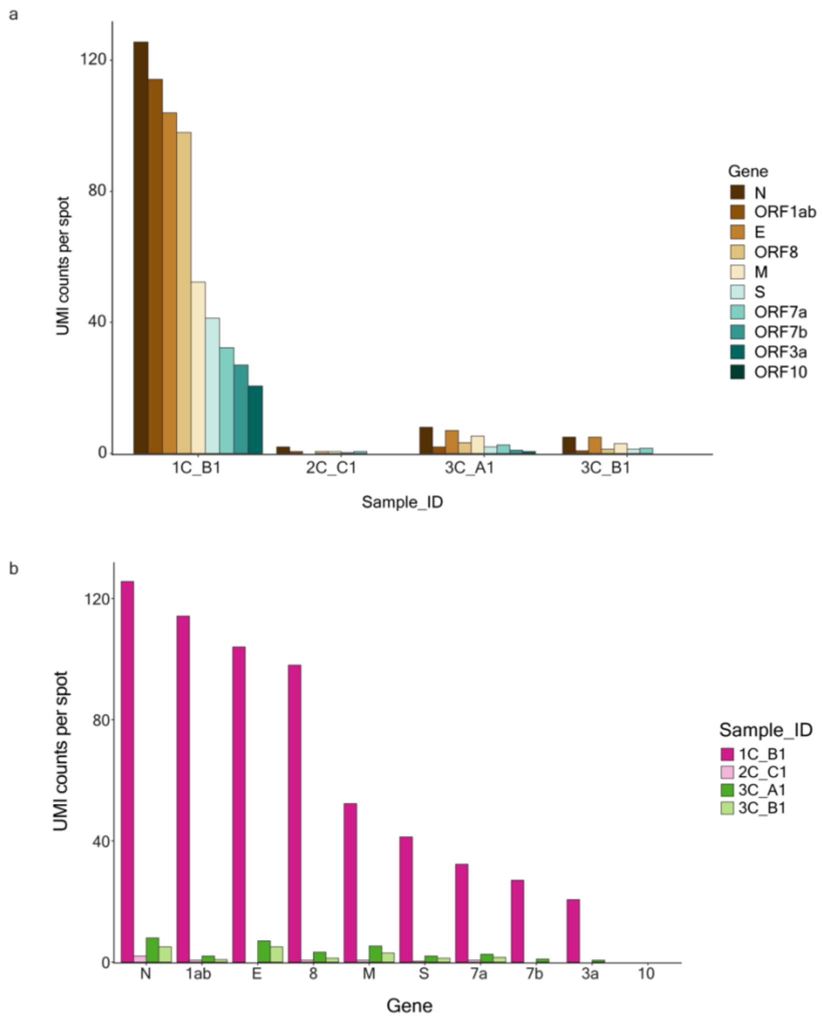


**Figure S10. SARS-CoV-2 gene distributions for each sample section assayed.** (a) Total UMI counts per spot of each SARS-CoV-2 gene for each COVID-19 sample section (n=4 sections). (b) Total UMI counts per spot of each SARS-CoV-2 gene across each COVID-19 sample section (n=4 sections).

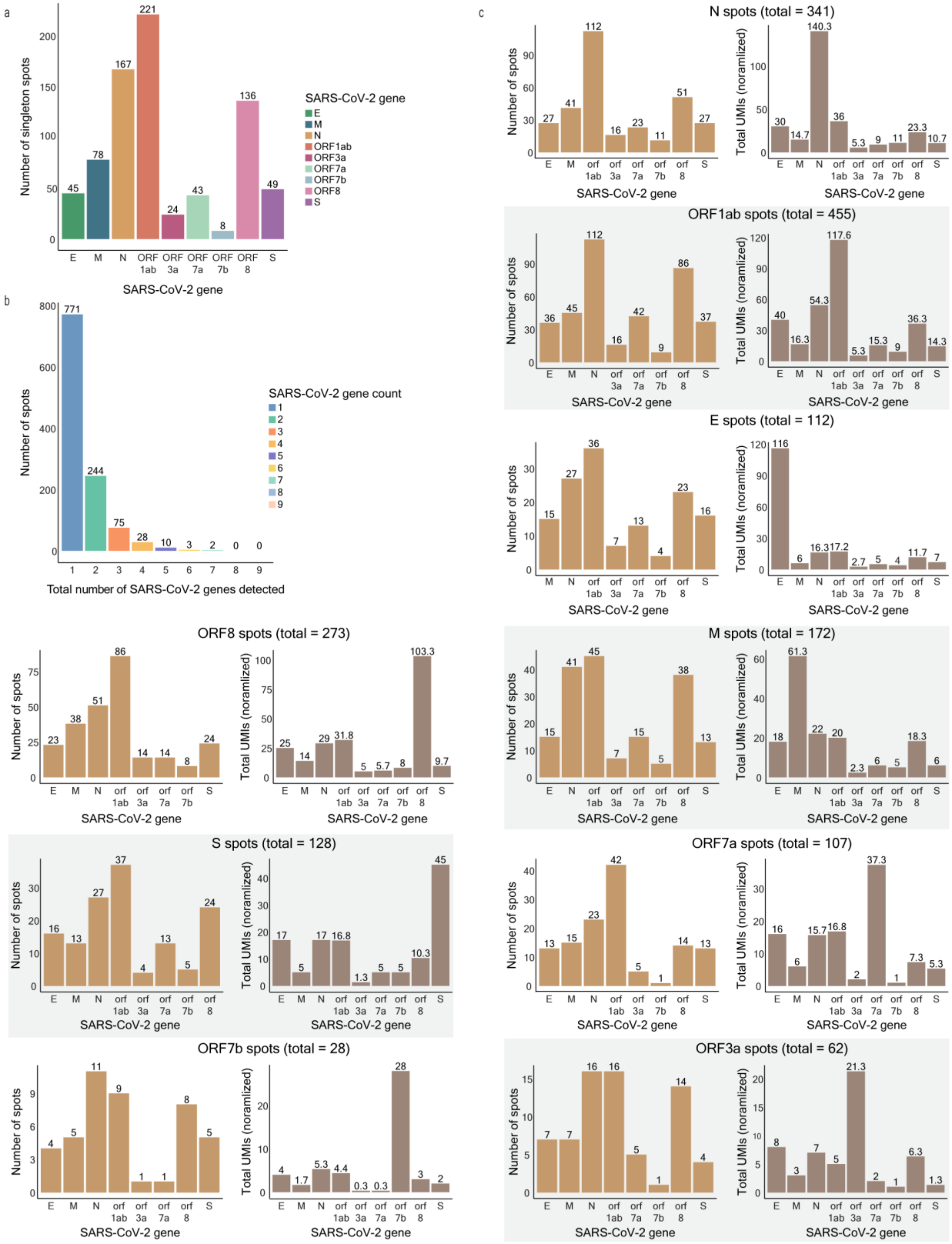
a
Number of singleton spots
SARS-CoV-2 gene
E
M
N
ORF1ab
ORF3a
ORF7a
ORF7b
ORF8
S
45
78
167
221
24
43
8
136
49
b
Number of spots
SARS-CoV-2 gene count
Total number of SARS-CoV-2 genes detected
771
244
75
28
10
3
2
0
0
c
N spots (total = 341)
ORF1ab spots (total = 455)
E spots (total = 112)
M spots (total = 172)
ORF8 spots (total = 273)
S spots (total = 128)
ORF7a spots (total = 107)
ORF7b spots (total = 28)
ORF3a spots (total = 62)
Number of spots
Total UMIs (noramlized)
SARS-CoV-2 gene

**Figure S11. SARS-CoV-2 gene abundance dynamics.** (a) The total number of singleton spots for each SARS-CoV-2 gene. (b) The total number of spots that 1, 2, 3, 4, 5, 6, 7, 8, or 9 different SARS-CoV-2 genes are detected in. (c) Number of spots and total UMI counts for each SARS-CoV-2 gene within the set of spots each particular SARS-CoV-2 gene is detected in.

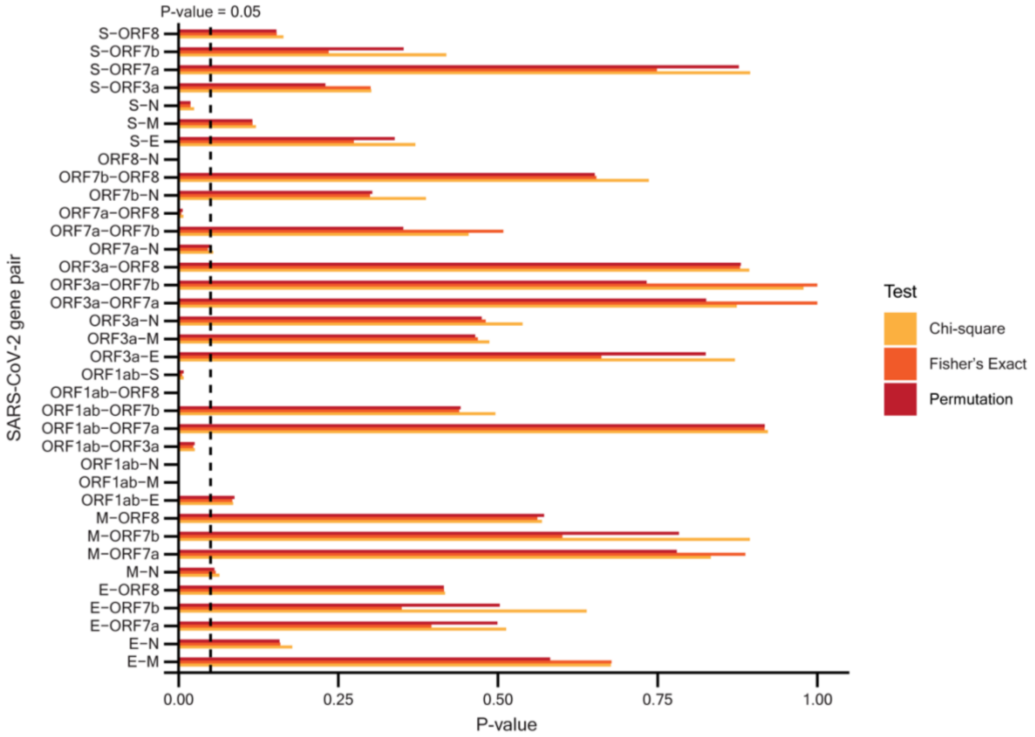


**Figure S12. SARS-CoV-2 gene colocalization metrics.** Analytical p-values for the colocalization of SARS-CoV-2 gene pairs across the SARS-CoV-2+ spots from Chi-Square test for independence, Fisher's exact test, and Approximative (Monte Carlo) Pearson chi-squared test for independence (Permutation). The dotted vertical line indicates a significance threshold p-value of 0.05.

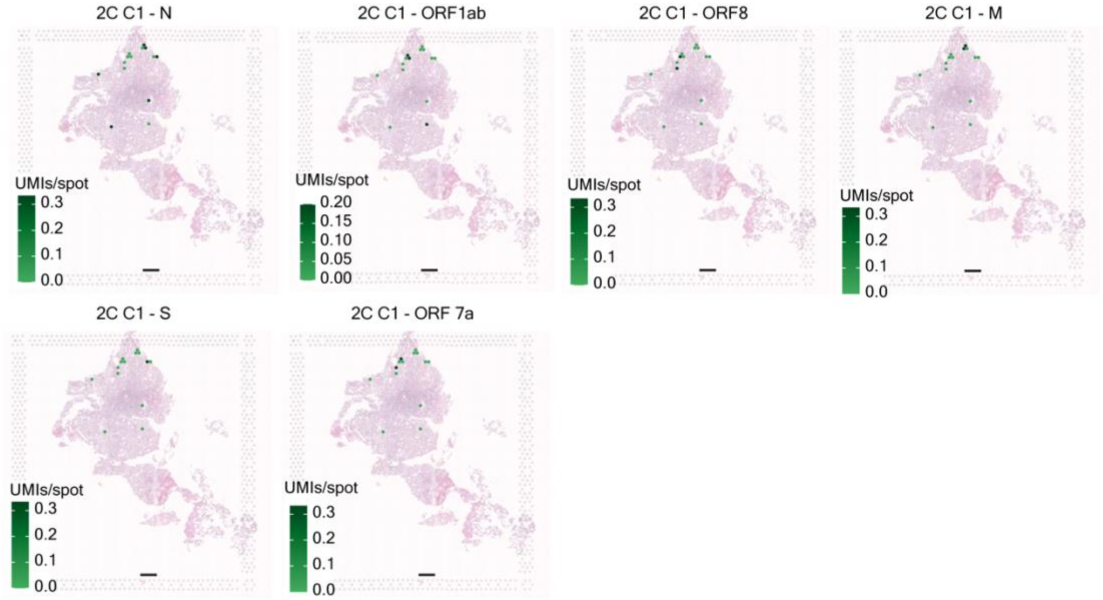


**Figure S13. Spatial SARS-CoV-2 gene distributions across tissue sample section 2C C1.** The spatial distribution of UMI counts per capture spot of each SARS-CoV-2 gene across COVID-19 sample section 2C C1. Scale bars are 500μm.

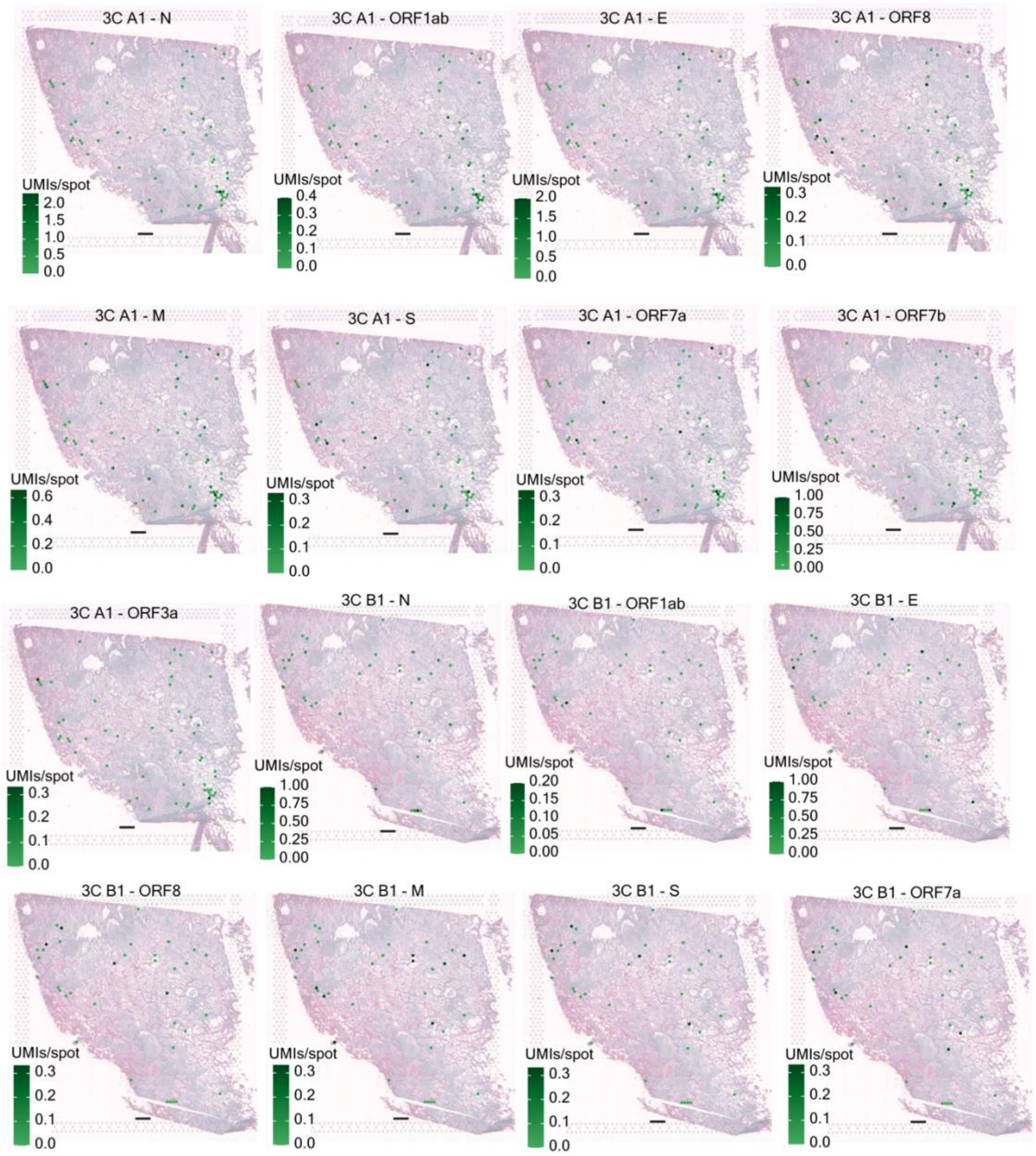


**Figure S14. Spatial SARS-CoV-2 gene distributions across tissue sample 3C.** The spatial distribution of UMI counts per capture spot of each SARS-CoV-2 gene across COVID-19 sample sections 3C A1 and 3C B1. Scale bars are 500μm.

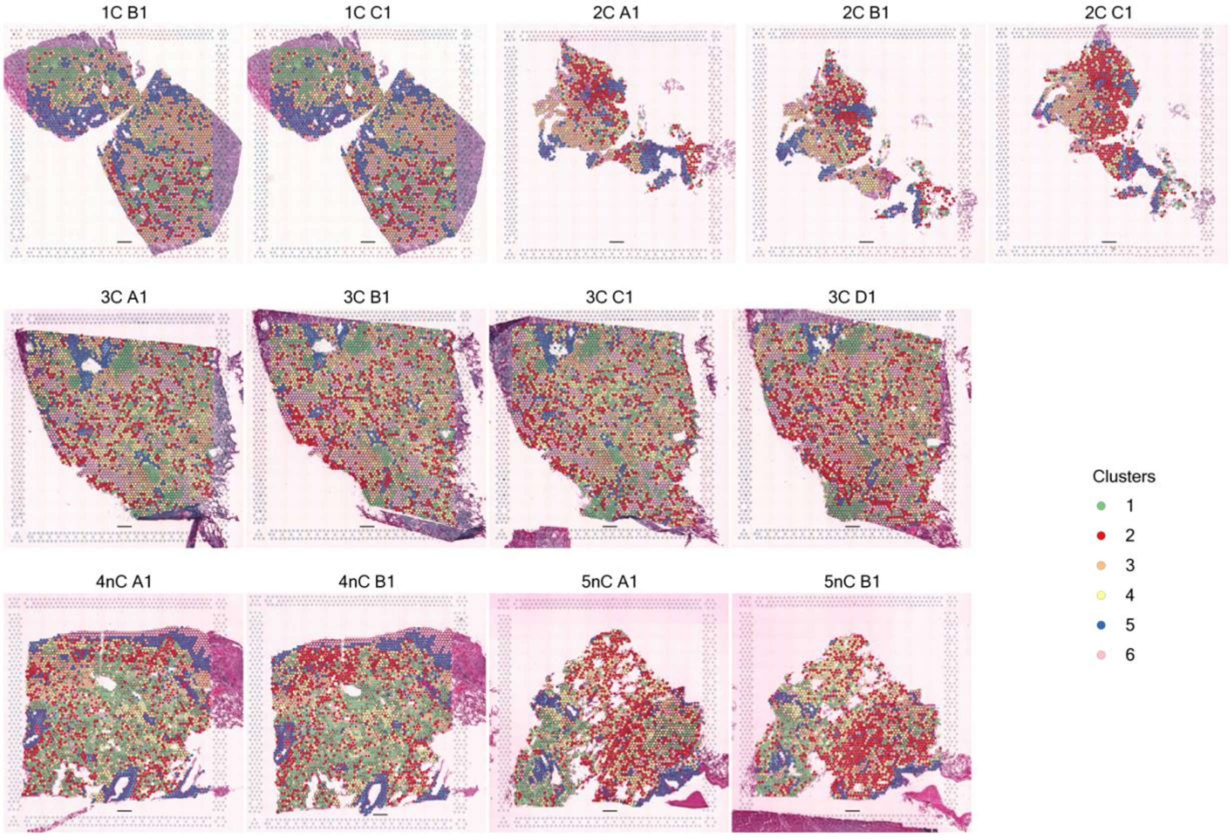


**Figure S15. Clusters mapped onto the 13 ST tissue sections.** Spatial distribution of the clusters on COVID-19 (1C, 2C, 3C) and control (4nC, 5nC) sections. Scale bars are 500µm. Differential expression analysis to identify cluster specific DE genes used Wilcoxon rank sum test and “bimod” Likelihood-ratio tests, $p < 0.05$.

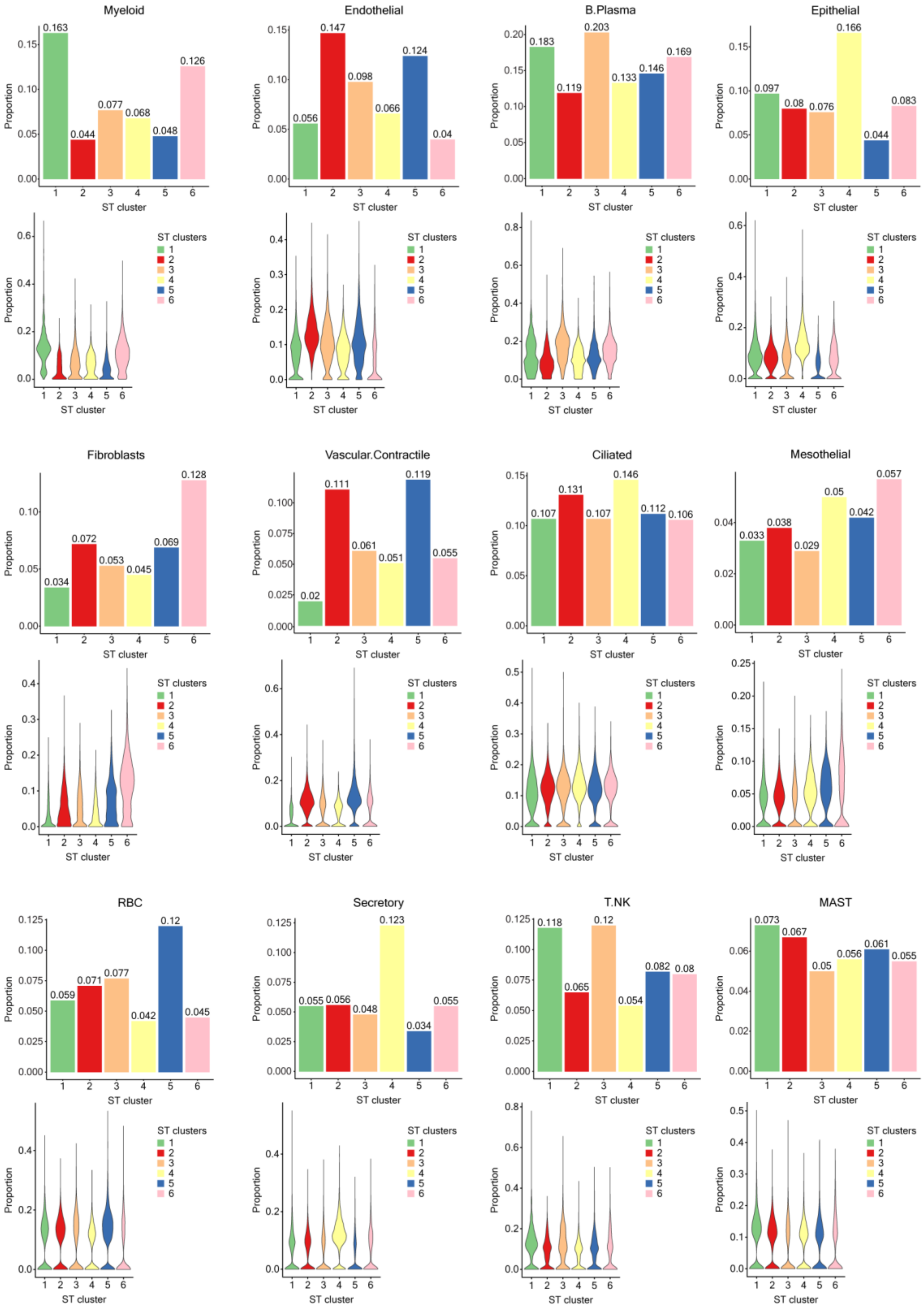
Myeloid
0.163
0.044
0.077
0.068
0.048
0.126
Endothelial
0.056
0.147
0.098
0.066
0.124
0.04
B.Plasma
0.183
0.119
0.203
0.133
0.146
0.169
Epithelial
0.097
0.08
0.076
0.166
0.044
0.083
Fibroblasts
0.034
0.072
0.053
0.045
0.069
0.128
Vascular.Contractile
0.02
0.111
0.061
0.051
0.119
0.055
Ciliated
0.107
0.131
0.107
0.146
0.112
0.106
Mesothelial
0.033
0.038
0.029
0.05
0.042
0.057
RBC
0.059
0.071
0.077
0.042
0.12
0.045
Secretory
0.055
0.056
0.048
0.123
0.034
0.055
T.NK
0.118
0.065
0.12
0.054
0.082
0.08
MAST
0.073
0.067
0.05
0.056
0.061
0.055
Proportion
ST cluster
ST clusters
1
2
3
4
5
6

**Figure S16. Single cell deconvolution of ST clusters.** Proportion of each single cell cell type across the ST clusters (top plots per cell type) and distribution of the proportion of each single cell cell type across the ST clusters (bottom plots per cell type).

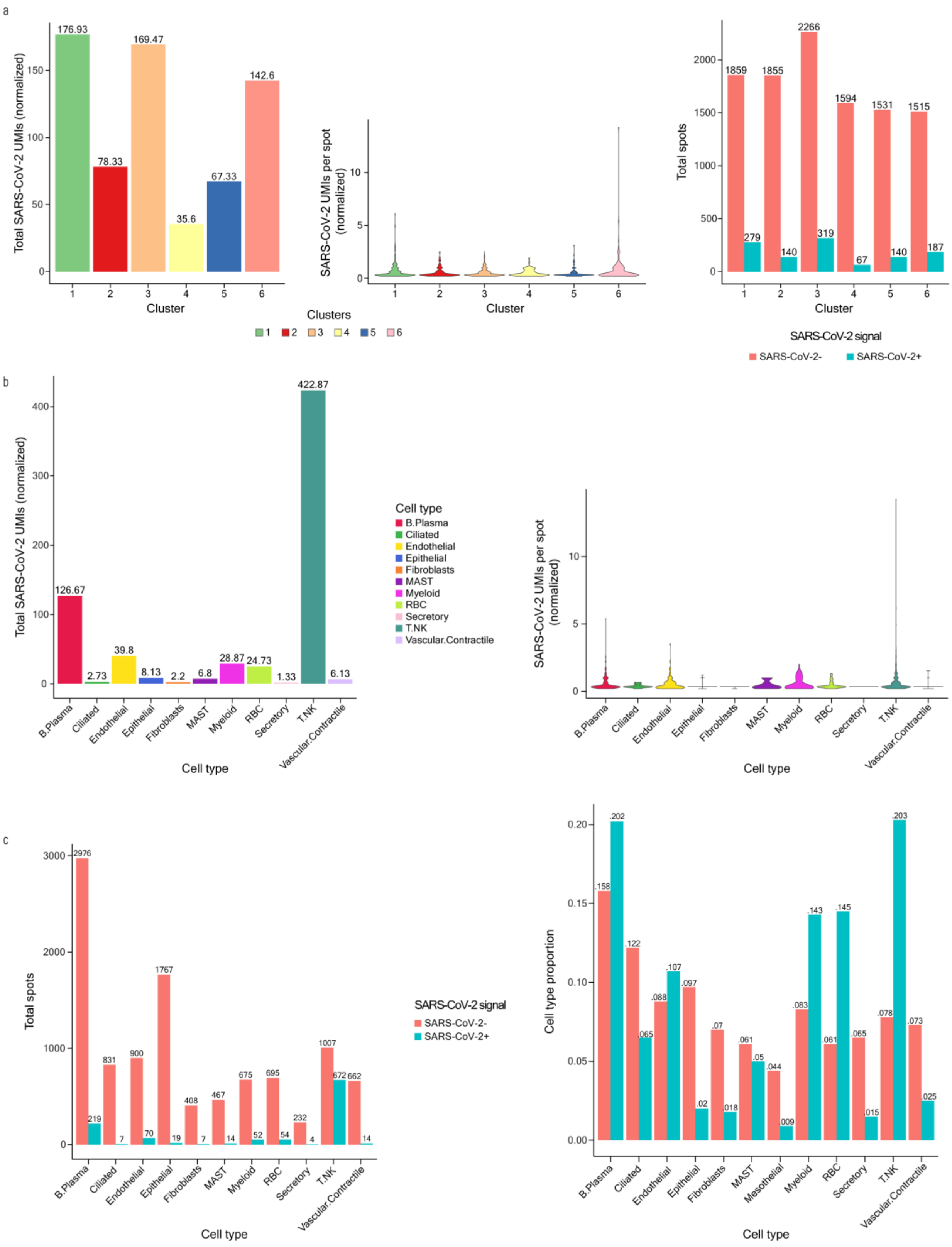
a
Total SARS-CoV-2 UMIs (normalized)
176.93
78.33
169.47
35.6
67.33
142.6
Cluster
Clusters
1 2 3 4 5 6
SARS-CoV-2 UMIs per spot (normalized)
Cluster
Total spots
1859 1855 2266 1594 1531 1515
279 140 319 67 140 187
Cluster
SARS-CoV-2 signal
SARS-CoV-2-
SARS-CoV-2+
b
Total SARS-CoV-2 UMIs (normalized)
422.87
126.67
2.73
39.8
8.13
2.2
6.8
28.87
24.73
1.33
6.13
Cell type
B.Plasma
Ciliated
Endothelial
Epithelial
Fibroblasts
MAST
Myeloid
RBC
Secretory
T.NK
Vascular.Contractile
SARS-CoV-2 UMIs per spot (normalized)
Cell type
c
Total spots
2976 219 831 7 900 70 1767 19 408 7 467 14 675 52 695 54 232 4 1007 672 662 14
Cell type
SARS-CoV-2 signal
SARS-CoV-2-
SARS-CoV-2+
Cell type proportion
.158 .202 .122 .065 .088 .107 .097 .02 .07 .018 .061 .05 .044 .009 .083 .143 .061 .145 .065 .015 .078 .203 .073 .025
Mesothelial
Cell type

**Figure S17. Viral enrichment across ST clusters and single cell cell types.** (a) Total SARS-CoV-2 UMI counts across all SARS-CoV-2 genes detected per ST cluster (left), distribution of SARS-CoV-2 UMI counts across all SARS-CoV-2 genes per spot across the ST clusters (middle), and total number of SARS-CoV-2$^{+}$ spots (blue) and SARS-CoV-2$^{-}$ spots (red) per ST cluster (right). (b) Total SARS-CoV-2 UMI counts across all SARS-CoV-2 genes detected per deconvolution-based cluster (left) and distribution of SARS-CoV-2 UMI counts across all SARS-CoV-2 genes per spot across the deconvolution-based clusters (right). (c) Total number of SARS-CoV-2$^{+}$ spots (blue) and SARS-CoV-2$^{-}$ spots (red) per deconvolution-based cluster (left) and cell type proportion (for each cell type) within SARS-CoV-2$^{+}$ spots (blue) and SARS-CoV-2$^{-}$ spots (red) (right).

## Supplementary Tables

| | ST-positive | ST-negative |
|---|---|---|
| **RNAScope-positive** | 46 | 113 |
| **RNAScope-negative** | 43 | 139 |

**Table S3. Confusion matrix comparing RNAScope and ST SARS-CoV-2 S gene signal**. The confusion matrix is generated from 300 µm disk areas used in the quantitative validation analysis that determines the SARS-CoV-2 S gene signal in each area for RNAScope and our ST approach.
