# Supplementary material for: Dual spatially resolved transcriptomics for human host–pathogen colocalization studies in FFPE tissue sections
Source: Genome Biol. 2023 Oct 19;24:237. doi: 10.1186/s13059-023-03080-y (PMC10588020; doi:10.1186/s13059-023-03080-y)
Supplement: Supplementary file 14 — Additional file 14. Review history. [file 13059_2023_3080_MOESM14_ESM.docx]

Review History

**First round of review**

**Reviewer 1**

**Were you able to assess all statistics in the manuscript, including the appropriateness of statistical tests used?**

There are no statistics in the manuscript.

**Comments to author:**

General

In this manuscript, Sounart et al describe a novel approach to analyze the host-pathogen interaction. Particular focus is put on the interaction between human lungs and SAR-CoV-2 virus. For this purpose, the authors design a set of probes for the SAR-Cov-2 transcripts (10 genes) to be used for the VISIUM FFPE platform. Mixing these probes with those for the human transcriptome, simultaneous detection of the human and viral transcriptomes is enabled. The precise detection of the tissue parts having viral transcripts is validated by RNAScope and in situ sequencing (ISS), which showed an overall good consistency. To demonstrate the performance of the developed platform, the authors subjected a total of 13 lung FFPE specimens harvested from five postmortem patients of COVID-19 to this analysis. They found that several categories of the human genes are differentially expressed between the regions of viral infections and those of not. Some of these expressions are consistent with the results of the previous studies. Others are novel to this study, although no direct validation analysis was conducted for those cases in this particular study.

Overall, I consider that this platform model should be useful for analyzing the host-pathogen interactions not only for COVID-19, but also future infectious diseases. Admitting overall importance, I have a remaining concern, that is, on the quantitative measurement of the viral transcripts.

Major comments:

1. How accurate do the detected signals (or sequence counts) represent the viral transcripts? The extent of the quantitative measurements should be further carefully evaluated. Particularly, detection at the "absolute" expression level (as of the counts of number of molecules), instead of "relative" expression level, should be desirable. In fact, VISIUM is a good platform for the "relative" expression level analysis. However, given the efficacy of the hybridization for each probe is not always equal, it may not be regarded as a "absolute" molecular count. Nevertheless, the quantitation for the viral load or viral transcript loads at the "absolute expression level" should have a very important meaning. Cannot be some spike-in controls utilized?

2. Detected expression levels of the transcripts should be compared to the viral genome load of the cell. Given the viral genome size is not large, is not it possible to employ a set of tiling probes covering the entire viral genome, regardless of expressed or not (or rarely) expressed regions?

3. The detection limit of the viral load and transcript levels should be further scrutinized. For this purpose, animal models having varying viral loads may be useful.

Minor comments:

4. The writing style should be revised. Many of the observed biological features as described in this manuscript have been previously reported. Or, at least, they are not directly confirmed by other biological assays for the materials presented here. In fact, a number of papers have been published in these two years, having a similar focus, also including spatial analysis of the host-pathogen interactions. For example, Margaroli et al (Cell Reports Medicine, 2021) demonstrated spatial profiling of SARS-CoV-2 and H1N1infected lung. Desai et al analyzed temporal and spatial heterogeneity of the SARS-CoV-2-infected samples (Nature Communication, 2020). Therefore, I'd recommend the "observation part" should be toned-down and more focus should be put on the methodological development.

5. Relatedly, a major fraction of the "observation part" should be made concise and the related descriptions should be moved to the Discussion section. For the discussion, the authors should also mention previous studies focusing on host-pathogen transcriptome signature during the SARS-CoV-2 infection made at the single-cell level, for example, Bost et al (Cell, 2020).

6. In this paper, the authors compared the host gene expression only by SARS-CoV-2 positive and negative. Generally, as described above, further quantitative measurements should widen the view of this analysis. For example, may not the viral transcriptome figures differ, depending on the interactions with immune cells? Or is not it different between different viral strains? (Also, please specify the viral strain and their mutation patterns, if any, used in this study somewhere.)

7. Judging from on "Patients selection, sample collection and processing" in the Methods section, it seems the authors have employed COVID-19 autopsy cases without considering their severity, treatment and other features. A number of papers have described that the clinical symptoms and related infection phenomena are truly diverse. At least some background information of on the clinical records of the patients should be presented in more details, further than Supplemental Table 2. Further detailed analyses on the relation between the patients' background should be also plausible.

8. As a method paper, it is desirable that the authors should demonstrate the power of this approach for other infectious diseases. At least, some future perspectives should be described in a more concrete manner.

9, It seems that some descriptions about the spatial analysis in the Background section should be updated, as the situation surrounding this area is rapidly changing. For example, they wrote previous methods can provide only "10 to 1000 genes", but a very recent paper reported that MERFISH can analyze >1000 genes (Fang et al, Science 2022).

**Reviewer 2**

**Were you able to assess all statistics in the manuscript, including the appropriateness of statistical tests used?**

Yes, and I have assessed the statistics in my report.

**Comments to author:**

In this manuscript, Sounart and colleagues have performed a spatial characterization of the COVID-19 lung by capturing and sequencing concurrently host and SARS-CoV-2 RNA in situ. The approach to expand the commercial 10x Genomics FFPE assay with SARS-CoV-2 probes enables not only an efficient capture of the pathogen RNA but also the ability to analyze effectively FFPE samples, which are way more abundant and easier to access in clinical settings. Even though the manuscript is a "Reseach" and not a "Method" article, the authors do not maximally utilize the rich datasets they have generated, in terms of bioinformatic analysis, but also in comparison with other similar efforts or public data integration. My enthusiasm remains high for this project, since the team has the experience and data to go deeper in the analysis of these samples. My comments are provided in further detail below:

1) The manuscript could benefit from proof-reading. There are syntax/grammar issues and typos.
2) There are different efforts that have attempted to perform similar host-pathogen spatial characterizations for COVID-19 samples or different settings. I believe that since it's a very novel domain, it would be helpful for the Readers if these efforts were briefly mentioned and commented. It would also put into perspective the innovation aspect of this manuscript and the potential remaining challenges. Even though I might be missing other studies, some that come to mind include:
A) Studies that employed spatial assays to characterize lung tissue from COVID-19 patients. E.g. Visium + RNAScope: e.g. Shojaei et al, MedRxiv 2021, or Visium + immunohistochemical capture of SARS-CoV-2 nucleocapsid: Mothes et al MedRxiv 2022. Villacampa et al (from the Lundeberg lab), performed an untargeted in situ transcriptomics assay on a lung sample from a COVID-19 patient but the viral load of the sample seemed to be very low, and was unclear whether the low yield was due to the low sensitivity of the method or the lack of SARS-CoV-2 RNA in the sample.
B) Studies that expanded existing spatial investigations of COVID-19 lung samples with targeted probes for SARS-CoV-2, such as Delorey et al, Nature 2021, where they added SARS-CoV-2 specific probes in the NanoString GeoMx Whole Transcriptome Atlas assay, or Kulasinghe et al, again using the GeoMx assay. Both studies were performed completely or partially on FFPE samples using assay extension with SARS-CoV-2 probes.
C) High resolution custom spatial assays to capture host-pathogen interactions not in COVID-19 settings, such as the efforts of Lötstedt et all (Biorxiv 2022), who recently performed a similar approach which they termed spatial host-microbiome sequencing (SHM-seq) to capture microbes and host genes in mouse intestine samples or the approach of Saarenpää et al (Giacomello Lab) who employed degenerate probes to capture bacterial and archaeal 16S rRNA hypervariable regions, together with poly-d(T) probes for host mRNA.
There are more, but the above show that a) this development did not take place in a vacuum, while on the other hand capture that even though there are approaches capable to tackle FFPE sample analysis with targeted SARS-CoV-2 probes or 55um resolution methods that work on frozen tissue, there is no method combining the specific characteristics of the presented approach (55um resolution + FFPE support).
3) The Visium dataset is treated mostly like a single cell dataset in terms of bionformatic analyses. This is evident in the pipeline followed, in the lack of spatial/Visium-specific analyses (QC approaches, lack of spatially-aware clustering, neighborhood/spatial region calling, spatially-aware cell-cell communication, spot deconvolution, etc. This is a major missed opportunity from a group specializing in the domain.
4) Since this is a Research manuscript (and not a Method), I would have expected also efforts to integrate the generated data with the spatial and single cell data generated from lung samples from COVID-19 patients as well as efforts to leverage knowledge and observations to generate new knowledge. For the latter, there is mostly a single section and feels rather forced, not as the main topic of the manuscript.
5) Following up on the above, the authors could integrate or compare against the currently available datasets with single cell and spatial data (GeoMx, Visium, RNAScope) from COVID-19 lung samples as well as use the public snRNAseq/scRNASeq samples for deconvolution and label transfer.
6) The authors observed that "SARS-CoV-2+ spots appeared throughout different morphological areas without obvious enrichment in any of the spatial clusters". However, in single cell publications examining SARS-CoV-2 abundance in lung samples from human patients, there is clear enrichment of SARS-CoV-2 in specific cell types (e.g. Delorey et al, Nature 2021). Could the authors comment on these differences?
7) The comparison of SARS-CoV-2+ and SARS-CoV-2- spot comparisons can be a bit more nuanced than as approached, since viral abundance can differacross architectural components or cell types. Could the analysis be repeated by matching the regions compared (e.g. alveoli vs alveoli, bronchial epithelium vs bronchial epithelium), cluster types (if possible), as well as cellular proportions? Since the comparisons could be confounded by the differences in cellular proportions, and lead to performing a DE between cell populations.
8) The presence of SARS-CoV-2 reads in the myeloid compartment could potentially point to phagocytosis as opposed to infection (e.g. as potentially observed in the epithelial compartments). Could the authors comment and expand?
9) The authors mention a set of suggested genes: SRRM2, HNRNPA2/B1, SMAP2, ARHGEF1, and TCIRG1 as "plausible novel targets for molecular interventions aiming to block COVID-19 pathogenesis in the human lungs". Could they expand on why they consider them to be plausible targets and how they envision the function of targeted therapeutics? To my understanding, the main supporting data is that these targets were differentially expressed in the SARS-CoV-2 RNA+ spots.
10) The analysis is performed en bloc with all the SARS-CoV-2+ spots, regardless of the cluster type. I would be very cautious lumping them all together, since some (as mentioned above) could be through phagocytosis or ambient RNA. There is also RNA transfer across spots which was also not evaluated during the QC (as was mentioned the QC was similar to that followed for single cell data).
11) The authors mention that SRRM2 could be a substrate for SARS-CoV-2. Do the same spots co-express other known factors/co-factors for SARS-CoV-2? In published studies of tissue data from deceased/severe patients, there is usually low expression of the molecules known or hypothesized to be factors/co-factors for SARS-CoV-2 entry, such as ACE2 or TMPRSS2 in the SARS-CoV-2 RNA+ cells. Therefore, detecting a gene up-regulated in the (+) sub-fraction, doesn't automatically make it a potential case for an entry or interaction molecule.
12) The authors mention "our in situ spatial analysis provides the first confirmation of these expressional changes to be directly associated to cells actively expressing viral components in the COVID-19 diseased human lungs". The resolution of the assay utilized does not enable this conclusion since in the same spot there can be a collection of different cells (5+ for the lung). It's impossible to identify if the signal is coming directly from a specific cell, unless a complementary assay is performed that permits single cell resolution. Alternatively, the authors could use publicly available single cell (nucleus) data from lung samples of COVID-19 patients to show that indeed these expression differences take place in the SARS-CoV-2 RNA+ subfraction when compared to cells from the same cluster/population without SARS-CoV-2 RNA presence.

Minor Comments
13) The high variability of the expression levels observed in the M, E, S, ORF1ab, ORF3a, ORF7a, ORF7b, and ORF8 genes was observed also across the SARS-CoV-2+ spots within the same slide or the overall ranking was consistent per slide but differed mostly across slides?
14) P-values reported: many pvalues are reported as < 2.2 * 10-16. This is a common issue dictated by technical reasons (machine epsilon) from the programming language or statistical package used. Can the authors substitute with the actual p-values observed? Could they also add a mention of the statistical test performed in the text and figures as well as the p-values when describing the outcome of statistical test?
15) When working with pathogen RNA in single cell or spatial, you want to ascertain that you have a bona fide event. Some helpful statistics could include: a) the co-occurrence of expressed viral genes in the same spots (pairwise and in larger combinations), b) the spots where a singleton viral gene expression event occurs, c) correlation of SARS-CoV-2 gene expression levels (UMIs) vs the average UMI counts per spot in a slide. Could these statistics be added and plotted?
16) The color scales in some graphs (e.g. Figure 4d, e) are not the same, leading to a different meaning for each color.
17) Fig 5b: I find the colocalization plots quite challenging as implemented to show the co-occurrence of SARS-CoV-2 RNA and of the relevant gene. For instance, it's very difficult to discern differences (at least for me) between + and - for SRRM2 and potentially CXCL9. Could the color scale or a two-axis color gradient (viral RNA abundance on the X axis and UMIs on the Y) be more helpful?
18) Line 446: There is an abbreviation of ORF10 as 10x Genomics
19) Novelty Score: The term isn't widely used. The formula should be added for the log10 UMI/gene ratio calculation for reference.
20) Registration: Adobe photoshop was used for image registration. How was registration quality evaluated? Would an elastic registration that can tackle tissue deformations from sectioning be preferable?

**Authors Response**

**Point-by-point responses to the reviewers’ comments:**

Reviewer #1

General

In this manuscript, Sounart et al describe a novel approach to analyze the host-pathogen interaction. Particular focus is put on the interaction between human lungs and SAR-CoV-2 virus. For this purpose, the authors design a set of probes for the SAR-Cov-2 transcripts (10 genes) to be used for the VISIUM FFPE platform. Mixing these probes with those for the human transcriptome, simultaneous detection of the human and viral transcriptomes is enabled. The precise detection of the tissue parts having viral transcripts is validated by RNAScope and in situ sequencing (ISS), which showed an overall good consistency. To demonstrate the performance of the developed platform, the authors subjected a total of 13 lung FFPE specimens harvested from five postmortem patients of COVID-19 to this analysis. They found that several categories of the human genes are differentially expressed between the regions of viral infections and those of not. Some of these expressions are consistent with the results of the previous studies. Others are novel to this study, although no direct validation analysis was conducted for those cases in this particular study.
Overall, I consider that this platform model should be useful for analyzing the host-pathogen interactions not only for COVID-19, but also future infectious diseases. Admitting overall importance, I have a remaining concern, that is, on the quantitative measurement of the viral transcripts.

Thank you for your comments, we are happy to hear that the reviewer finds the work of interest for the broad use of studying host-pathogen interactions in future infectious diseases. We have addressed the reviewer’s comments below, with a particular focus on the concern of the quantitative measurement of the viral transcripts.

Major comments:

1. How accurate do the detected signals (or sequence counts) represent the viral transcripts? The extent of the quantitative measurements should be further carefully evaluated. Particularly, detection at the "absolute" expression level (as of the counts of number of molecules), instead of "relative" expression level, should be desirable. In fact, VISIUM is a good platform for the "relative" expression level analysis. However, given the efficacy of the hybridization for each probe is not always equal, it may not be regarded as an "absolute" molecular count. Nevertheless, the quantitation for the viral load or viral transcript loads at the "absolute expression level" should have a very important meaning. Cannot be some spike-in controls utilized?

We agree with the reviewer that the measurement of the viral load and number of transcripts on an absolute level would provide useful information regarding the sensitivity of our viral detection and its comparability to other available methods for RNA quantification. This, however, requires a bona fide quantification method that can be reliably implemented in the analyzed sample type. The use of spike-in controls, suggested by the reviewer, would be an elegant way of addressing this question, but unfortunately, this procedure is not yet fully established for the Visium platform. Most alternative RNA detection methods (RNAscope, ISS) are also based on the hybridization of target-specific probes, where hybridization efficiency, even with optimal probe design, RNA quality and experimental conditions, never reaches 100%. Therefore, one must be cautious in interpreting the obtained results as absolute transcript values, even in the case of techniques with reportedly single transcript resolution(RNAscope ISH Technology, n.d.).

To address the reviewer’s comment, we further evaluated the viral load in our samples with an alternative approach by performing qPCR with primers targeting the E gene of the SARS-CoV-2 virus. For the qPCR experiments, we used RNA extracted from several sections (between 4 and 30 sections, depending on the area of the tissue piece) of the same tissue regions used for the spatial methods (ST, RNAScope, ISS). In some cases this meant including relatively distant segments of the tissue block compared to the sections used for spatial detection, thus we considered these samples to represent the overall viral load in these tissues. In the qPCR reactions, we also used a plasmid control with a known number of copies, which allowed us to calculate the theoretical copies per ng of RNA value for all analyzed samples. However, given the suboptimal RNA quality of our samples, the actual viral presence is likely higher than what we estimate based on this analysis. We compared log-transformed values of the summed UMI counts detected by the ST approach for all target SARS-CoV-2 genes, and separately for the E gene, to the copies per ng of RNA value calculated based on the E gene detection, and to the summed S gene-specific RNAscope signal. We saw highly similar signal distribution across the samples with all detection methods, except for the E gene not being detected by ST in one sample, likely due to a low viral load and lower RNA quality (DV200: 45) compared to the other samples. This semi-quantitative analysis corroborates our previous results, showing a good overall agreement between these methods. The results of this analysis are presented in Supplementary Figure 7 and lines 150-153 and 742-755.

In addition, we thoroughly validated our Visium approach on consecutive sections analyzed with RNAscope and with the ISS platform. Since RNAscope is reported to have the highest sensitivity of these technologies and to detect target genes on a single transcript level(RNAscope ISH Technology, n.d.), we considered this detection method as a gold-standard reference for close-to-absolute transcript levels. To compare the virus-related signal levels detected across different platforms, we expanded our previous analysis of comparing the overlap between SARS-CoV-2+ and SARS-CoV-2- spots in a binary fashion. For this, we further quantified the RNAscope signal obtained with a probe against the S gene and the in situ sequencing (ISS) S gene signal, and compared the signal density to the S gene-specific Visium counts in the corresponding tissue areas in one COVID-19 lung sample with the highest signal values. To increase the precision of our comparison and allow for minor positional differences between the signals originating from consecutive sections, we implemented elastic image registration to align the ST, RNAscope, and ISS signal images and then binned the signals into circular disks of 300 µm diameter by merging every seven neighboring ST spots (one central and its six neighbors). With this new validation approach, we found a 72.44% specificity of ST to RNAScope S gene detection for this sample. We also quantified the SARS-CoV-2 specific signal (S gene) captured with ISS, from a non-consecutive (~300 µm distance) section, and obtained an overall similar spatial distribution to the other two methods, and a 58.99% specificity of the ST to ISS signal detection. These values are somewhat below the specificity determined for all three pathological samples by the square-based binning used for the binary (SARS-CoV-2+ vs. SARS-CoV-2-) categorization discussed in the manuscript, probably due to the increase in the bin area. We found a similar spatial pattern in the S signal heatmap and signal intensity distribution between the three detection methods, supporting their comparability even when performing analysis in a quantitative manner. We present these results in Figure1e-f and in lines 147-150, 668-679, 705-722, and 740-741.

Lastly, we emphasize that to enable working with infectious human material in BSL-1 conditions, we established our dual spatial transcriptomics platform for compatibility with FFPE tissue samples, where tissue fixation inactivates the virus. A trade-off of FFPE fixation and embedding is lower RNA quality, where RNA degradation can affect viral detection with methods based on different probe sets and experimental protocols, limiting their comparability. The RNA quality of our samples (2-2.4 RIN, 45-72% DV200, Supplementary Table 2) is within a typical range for FFPE material and largely falls into the Illumina scale(Illumina. Evaluating RNA Quality from FFPE Samples. Technical Note: RNA Sequencing. Https://emea.illumina.com/content/dam/illumina-Marketing/documents/products/technotes/evaluating-Rna-Quality-from-Ffpe-Samples-Technical-Note-470-2014-001.pdf, n.d.) medium-quality category (50-70% DV200). However, even with this level of RNA quality in our samples, we were able to demonstrate a thorough, quantitative validation of our ST-based viral detection with qPCR, RNAScope, and ISS.

2. Detected expression levels of the transcripts should be compared to the viral genome load of the cell. Given the viral genome size is not large, is it not possible to employ a set of tiling probes covering the entire viral genome, regardless of expressed or not (or rarely) expressed regions?

We reasoned that since several of our probes target ORF1ab, which is not transcribed into subgenomic RNAs and thus is present only in the viral genomic RNA, detection of ORF1ab could be considered as representative of the viral genome load. However, even with the largest number of probes (5) targeting ORF1ab, we detected ORF1ab in only 40% of the SARS-CoV-2+ spots (referred to as ORF1ab+ spots). For 48.6% of these ORF1ab+ spots, ORF1ab was the only SARS-CoV-2 transcript detected (termed singleton spots). We also detected other SARS-CoV-2 transcripts in singleton spots (meaning spots with only one SARS-CoV-2 gene detected). We previously observed various levels (as UMI counts) of different SARS-CoV-2 transcripts (as shown in Figure 3 of the manuscript), with trends that largely corresponded to that expected from subgenomic RNA (sgRNA), where higher levels come from transcripts closer to the 3’ end(Kim et al., 2020; Long, 2021). The portion of singleton spots also follows this trend of abundance, where more abundantly detected genes have more singleton spots. The results of this analysis are presented in the plots in Additional Figure 1.

In addition, as stated in response to comment #1, we performed a qPCR of the SARS-CoV-2 E gene to determine the overall viral load in our samples. We found similar trends between the qPCR detection of the overall levels of the virus and our detection by ST (across all viral genes and for the E gene), confirming the reliability of our approach.

Certain viruses have very small genomes, which can be successfully mapped using tiling probes in some instances. However, SARS-CoV-2, in particular, belongs to the Nidovirales order of positive-sense single-stranded RNA viruses that have some of the largest known viral genomes(Grellet et al., 2022; Saberi et al., 2018). The SARS-CoV-2 genome assembly used to design the probes in this study is 29,903 bp in size (https://covid-19.ensembl.org/Sars_cov_2/Info/Annotation), which would require about 600 of our 50 bp structured probes to tile, making such an approach infeasible. Of note, in our platform, we target 10 different viral genes, many of them with more than 1 probe, and thus cover a larger portion of the viral genome compared to other spatial methods (such as GeoMx [NanoString](Merritt et al., 2020; Zollinger et al., 2020) and RNAScope(Wang et al., 2012)) designed for SARS-CoV-2 detection in COVID-19 patient lungs(Butler et al., 2021; Delorey et al., 2021; Desai et al., 2020; Kulasinghe et al., 2021; Margaroli et al., 2021; Rendeiro et al., 2021). A more detailed exploration of the gRNA (viral genome load) to sgRNA (expression levels of the transcripts) would be of great use to the research community, however, the technical challenges described above puts such an endeavor outside of the current possible realm of this research study.

3. The detection limit of the viral load and transcript levels should be further scrutinized. For this purpose, animal models having varying viral loads may be useful.

While we agree that it would be beneficial to have an understanding of the detection limits of our platform, the discussed challenges regarding the absolute quantification of the viral load and transcript levels in our response to comments #1 and #2 make it difficult to perform such analysis in a reliable manner. While the use of well-characterized animal models could mitigate some of these issues, it also has various shortcomings that make other experimental approaches better suited to address this question:

1. Selecting an animal model that is both easily accessible and reliably reproduces the characteristics of human SARS-CoV-2 infection is challenging. While wild-type mouse strains would be the obvious choice for this type of work, other studies reported that SARS-CoV-2 showed low affinity to murine tissues, mainly due to sequence differences in major entry factors, such as ACE2, between the two species(Da Costa et al., 2022). To produce biologically relevant data, we would need to analyze infected tissue from human ACE2-expressing transgenic or humanized mice, or other, better suited animal species (i.e. non-human primates), that were unfortunately not accessible to us.

2. Local viral load in any target organ of infected animals is not necessarily directly proportional to the standardized inoculation doses. Assessment of the viral genome load reached in the lungs would require additional analysis steps such as qPCR from bulk lung RNA, which we performed on the human samples already included in the study, as discussed in response to comments #1 and #2.

Considering these points, we added region-specific qPCR against the E gene of SARS-CoV-2 (discussed in our reply to comment #1) to our validation approaches, reasoning that this would convey more reliable and relevant information than including new animal tissues in our study. We selected the E gene as the target SARS-Cov-2 gene, since among other qPCR probes available to us, that reaction showed the highest sensitivity. With all four SARS-CoV-2 detection methods applied (ST, RNAscope, ISS, and qPCR), we observed that the COVID-19 samples in our study covered a range of viral loads, likely close to the lower end of the clinically relevant spectrum. This is in line with the late disease stage of the samples, where other studies also described a lower SARS-CoV-2 load in comparison to the acute phase of the infection(Desai et al., 2020). In addition, a recent study focusing on the absolute quantification of SARS-CoV-2 genomic and subgenomic RNAs from nasal swabs reported high, medium, and low categories in terms of viral transcript levels for the ORF1ab, N, and S genes(Oranger et al., 2021). While the tested samples and target genes differ between the two approaches, it is worth noting that the copies per ng of RNA values obtained in our E-gene-specific qPCR fall into their “low viral content” category, providing further support to the sensitivity of our detection method. One must keep in mind, however, that the detection of the viral RNA is negatively affected by degradation, which is an additional reason why the quantification of the absolute viral RNA content and a direct comparison between the results of these technologies do not reliably describe the relationship between the SARS-CoV-2 viral load and transcripts in FFPE samples.

Minor comments:

4. The writing style should be revised. Many of the observed biological features as described in this manuscript have been previously reported. Or, at least, they are not directly confirmed by other biological assays for the materials presented here. In fact, a number of papers have been published in these two years, having a similar focus, also including spatial analysis of the host-pathogen interactions. For example, Margaroli et al (Cell Reports Medicine, 2021) demonstrated spatial profiling of SARS-CoV-2 and H1N1 infected lung. Desai et al analyzed temporal and spatial heterogeneity of the SARS-CoV-2-infected samples (Nature Communication, 2020). Therefore, I'd recommend the "observation part" should be toned-down and more focus should be put on methodological development.

We thank the reviewer for this suggestion. The studies mentioned by the reviewer(Desai et al., 2020; Margaroli et al., 2021), among others performing spatial analysis on lung tissue collected from COVID-19 patients, gave important contributions to understanding the organ’s response to the SARS-CoV-2 infection, and thus we have shortly discussed and referenced them in our manuscript in lines 76-88 and 409-414. It is important to note that both mentioned studies rely on the detection of a few SARS-CoV-2-specific gene targets or immunolabeling of a few viral protein components, which, assuming similar degradation of RNA in their samples and considering the high number of singleton SARS-CoV-2 transcript spots in our dataset, might limit their viral detection and the drawn biological conclusions. Furthermore, our study encompasses a different scope by spatially detecting SARS-CoV-2 through targeting 10 transcripts of the virus to spatially co-resolve both host and pathogen transcriptomes on the same tissue section without preselection of a region of interest, and thus provides an unbiased view of the tissue response to viral infection.

In addition, we toned down the biological portions of our manuscript to shift the focus to the presented methodological development in lines 229-297 and 307-375, and correspondingly changed the format of the manuscript to a Method article.

5. Relatedly, a major fraction of the "observation part" should be made concise and the related descriptions should be moved to the Discussion section. For the discussion, the authors should also mention previous studies focusing on host-pathogen transcriptome signature during the SARS-CoV-2 infection made at the single-cell level, for example, Bost et al (Cell, 2020).

As stated in response to the previous comment and in line with changing the format of the manuscript to a Method paper, we toned down and made more concise the observational part of the manuscript in lines 229-297 and 307-375. We also included the suggested single-cell studies(Bost et al., 2020; Lamers & Haagmans, 2022; Melms et al., 2021; Ren et al., 2021; Tian et al., 2022) in our discussion and updated the related section according to the reviewer’s suggestions, as can be seen in lines 409-411.

6. In this paper, the authors compared the host gene expression only by SARS-CoV-2 positive and negative. Generally, as described above, further quantitative measurements should widen the view of this analysis. For example, may not the viral transcriptome figures differ, depending on the interactions with immune cells? Or is it not different between different viral strains? (Also, please specify the viral strain and their mutation patterns, if any, used in this study somewhere.)

We agree with the reviewer that the level of local viral load, and consequently of viral transcripts, can affect the host responses, as previous studies demonstrated(Butler et al., 2021; Desai et al., 2020; Kulasinghe et al., 2021; Margaroli et al., 2021; Park et al., 2022; Rendeiro et al., 2021).

To address the reviewer’s comment about assessing the interactions between the virus and the host cells in a more nuanced manner, we performed colocalization analysis within our separate ST clusters by performing differential expression (DE) gene analysis between SARS-CoV-2+ and SARS-CoV-2- spots within each ST cluster level. We found most of the highest-ranking DE genes to be shared across the clusters and showed a large overlap with the en masse DE genes.

To look more deeply into the interactions with immune cells, and in line with the request of the other reviewer, we ran deconvolution on our ST data using a publicly available single-cell dataset(Delorey et al., 2021) to determine the cell type distributions in our spatial spots. Assigning each ST spot to its dominant cell type resulted in 11 clusters (referred to as deconvolution-based clusters) that are in line with the number of cell types in the single cell dataset(Delorey et al., 2021) used for label transfer and largely confirmed our original, solely ST data-based cluster annotations.

To further address the reviewer’s point on how viral loads interact with immune cells, we observed that clusters dominated by immune cells (ST clusters 1 and 3, deconvolution-based B- , T/NK- and myeloid cell-dominated clusters) contained the highest levels of SARS-CoV-2 UMIs. Interestingly, the fibroblast-enriched ST cluster 6 has the third highest value of SARS-CoV-2 UMIs, while the fibroblast-dominated spots identified by deconvolution have one of the lowest levels; this suggests that the viral transcripts in cluster 6 spots likely originated from a non-fibroblast cell type, which was less prominent in the transcriptome captured from these ST spots. The cell type deconvolution identified T/NK cells in the tissue that were masked in our previous analysis due to their relatively low abundance. T/NK cell-dominated spots displayed the highest absolute and relative SARS-CoV-2-related UMI counts, in line with a potent cellular immune response against cells actively expressing viral components.

To reveal potential cell type-specific modulators of the host response, we generated DE genes between SARS-CoV-2+ and SARS-CoV-2- spots unique for each analyzed deconvolution-based cluster and found two immunoglobulin variable genes (IGLV9-49 and IGHV1-46) to be upregulated in the B-cell dominated deconvolution-based cluster; this may signal the presence of virus-reactive B-cell clones in the close proximity of the infected cells, and could warrant further research aiming to confirm the presence of such clones and to determine their BCR sequence in the hope of generating neutralizing antibodies against the virus. While it was beyond the scope of our project to perform an in-depth analysis of the immune response to the presence of the virus, these results demonstrate the potential of conducting such studies based on dual spatial transcriptome information obtained by our technique. The results of this analysis are presented in Supplementary Figure 14, and also in Supplementary Tables 9-10, and lines 256-271, 339-349, and 432-437.

To address the reviewer’s question on the differences across viral strains, we would like to clarify that the probes that bind to SARS-CoV-2 RNA used in the study were designed based on the Wuhan-Hu-1 isolate, Sars_cov_2.ASM985889v3, Ensembl build 101 (https://covid-19.ensembl.org/Sars_cov_2/Info/Index), as stated in the Methods section lines 491-494. In our assay, the left (LHS) and right (RHS) probes for each SARS-CoV-2 gene bound in proximity on the RNA target, are ligated, and then the probes themselves bind to the Visium slide and are ultimately sequenced. Then, the sequence data is mapped to a reference containing the different probe sequences to identify what viral genes are present (S gene, E gene, etc.) rather than the actual viral RNA being sequenced(10X Genomics. Visium Spatial Gene Expression Reagent Kits for FFPE User Guide. Document Number CG000407, Rev A. (2021).CG000407_VisiumSpatialGeneExpressionforFFPE_UserGuide_RevA.pdf, n.d.). Therefore, we are not able to identify the viral strains present in the samples with the specific probes used here. We added further clarification about this to the Methods section lines 494-495. Future users of the probes could run the sequences in GISAID (https://gisaid.org/) to see how the probes would bind to specific sequences or variants of interest. The probes are ordered from IDT and the sequences could be altered for specific variants of interest in future studies.

7. Judging from "Patients selection, sample collection and processing" in the Methods section, it seems the authors have employed COVID-19 autopsy cases without considering their severity, treatment and other features. A number of papers have described that the clinical symptoms and related infection phenomena are truly diverse. At least some background information on the clinical records of the patients should be presented in more details, further than Supplemental Table 2. Further detailed analyses on the relation between the patients' background should be also plausible.

In line with the reviewer’s request, we added the patients’ age, sex, and comorbidities in a pseudonymized manner to Supplementary Table 2 for the potential benefit of future studies reanalyzing our dataset. Nevertheless, we would like to point out that the scope of our study is presenting the methodological advancement of detecting spatial colocalization of both host and pathogen transcriptomes. Our goal was to cover different viral loads, aiming for the lower end of the clinically relevant range to demonstrate the sensitivity of our method, rather than covering a range of clinical parameters. The sample size of our study enabled us to demonstrate our novel technology and that we could detect the virus even with lower loads, however, this sample size does not suffice to draw any meaningful conclusion on the relation between our results and the available clinical parameters.

8. As a method paper, it is desirable that the authors should demonstrate the power of this approach for other infectious diseases. At least, some future perspectives should be described in a more concrete manner.

We agree with the reviewer that describing future perspectives in a more concrete manner will emphasize the power of our approach, and thus we added text in the discussion section to address this point in lines 438-448. Briefly, we think that dual host-pathogen spatial transcriptome analysis has immense potential to provide unique information in a wide range of infectious diseases, specifically about entry factors, host-provided machinery for replication, productive and potentially harmful cellular countermeasures against the presence of the pathogen, and cell-cell interactions, in a tissue-specific manner. The quick spread of similar techniques during the COVID-19 pandemic highlights the relevance of spatially aware approaches in understanding the pathogenesis of the disease. Specifically, we think our approach would be highly relevant for the spatial detection of already known RNA viruses, such as the hepatitis C virus (HCV) in the human liver to localize previously identified host dysregulated gene expression patterns/pathways(Hamdane et al., 2019) across involved host cell populations; this would be highly valuable for understanding the progression of HCV-induced liver disease. This knowledge could be crucial for identifying patients at risk of developing cirrhosis and cancer and highlight potential targets for future chemopreventive approaches. In the case of other recently emerged pathogens with substantial epidemiological relevance, such as SARS-CoV-1, DENV (Dengue virus), or ZIKV (Zika virus), or potential novel pathogens in the future, it would also be advantageous to adopt spatial technologies at the forefront of related research efforts.

Notably, we also observed an upregulation of a number of immunoglobulin variable genes in certain subsets of SARS-CoV-2+ spots, potentially representing virus-associated B cell clones. With novel spatial transcriptomics technologies expanding into lymphocyte clone detection in the tissue context(Engblom et al., 2022), such observations paired with antigen analysis could provide a basis for further research in the hope of identifying and generating neutralizing antibodies for clinical and scientific use, also in the case of other pathogens causing infectious diseases.

9. It seems that some descriptions about the spatial analysis in the Background section should be updated, as the situation surrounding this area is rapidly changing. For example, they wrote previous methods can provide only "10 to 1000 genes", but a very recent paper reported that MERFISH can analyze >1000 genes (Fang et al, Science 2022).

As the reviewer pointed out, the field of spatial transcriptome analysis is indeed rapidly evolving, and in line with this, we updated the background section of our revised manuscript with the following spatially-resolved transcriptomic methods in FFPE tissues: Xenium In Situ Technology [10X Genomics] (In Situ Gene Expression, n.d.; Janesick et al., 2022), Improved in situ sequencing (IISS) (X. Tang et al., 2022), HybISS as part of ISS from Cartana(Gyllborg et al., 2020), CosMX [Nanostring] (He et al., 2022), clampFISH 2.0 (Dardani et al., 2022), MOSAICA (Vu et al., 2022), and RNA-seq of FFPE PuTi-spot (Matsunaga et al., 2022) in lines 58-66. We focused on technologies that are compatible with FFPE tissues, as there are fewer such methods to date compared to ones adept at analyzing only fresh frozen tissues, and infectious tissues are most often stored in the form of FFPE blocks. The mentioned study by Fang et al.(Fang et al., 2022) managed to analyze more than 1000 genes, but only worked with fresh frozen rather than FFPE tissues. To our knowledge, MERFISH on FFPE tissue is not yet demonstrated to exceed 1000 genes. In line with this, the current MERSCOPE FFPE Solution (https://info.vizgen.com/merscope-ffpe-solution) appears to have gene panels for 500 genes, and a study from Tang et al.(Z. Tang et al., 2023) used this solution to spatially resolve 500 genes in FFPE tissues.

Reviewer #2

In this manuscript, Sounart and colleagues have performed a spatial characterization of the COVID-19 lung by capturing and sequencing concurrently host and SARS-CoV-2 RNA in situ. The approach to expand the commercial 10x Genomics FFPE assay with SARS-CoV-2 probes enables not only an efficient capture of the pathogen RNA but also the ability to analyze effectively FFPE samples, which are way more abundant and easier to access in clinical settings. Even though the manuscript is a "Research" and not a "Method" article, the authors do not maximally utilize the rich datasets they have generated, in terms of bioinformatic analysis, but also in comparison with other similar efforts or public data integration. My enthusiasm remains high for this project, since the team has the experience and data to go deeper in the analysis of these samples. My comments are provided in further detail below:

We are happy to hear that the reviewer finds our work valuable. We address the reviewer’s comment to explore our rich dataset more deeply and describe our new analysis and results in the response to the points below.

1) The manuscript could benefit from proof-reading. There are syntax/grammar issues and typos.

Thank you for your suggestion. We have further proofread the manuscript and addressed any syntax/grammar issues to the best of our knowledge.

2) There are different efforts that have attempted to perform similar host-pathogen spatial characterizations for COVID-19 samples or different settings. I believe that since it's a very novel domain, it would be helpful for the Readers if these efforts were briefly mentioned and commented on. It would also put into perspective the innovation aspect of this manuscript and the potential remaining challenges. Even though I might be missing other studies, some that come to mind include:
A) Studies that employed spatial assays to characterize lung tissue from COVID-19 patients. E.g. Visium + RNAScope: e.g. Shojaei et al, MedRxiv 2021, or Visium + immunohistochemical capture of SARS-CoV-2 nucleocapsid: Mothes et al MedRxiv 2022. Villacampa et al (from the Lundeberg lab), performed an untargeted in situ transcriptomics assay on a lung sample from a COVID-19 patient but the viral load of the sample seemed to be very low, and was unclear whether the low yield was due to the low sensitivity of the method or the lack of SARS-CoV-2 RNA in the sample.
B) Studies that expanded existing spatial investigations of COVID-19 lung samples with targeted probes for SARS-CoV-2, such as Delorey et al, Nature 2021, where they added SARS-CoV-2 specific probes in the NanoString GeoMx Whole Transcriptome Atlas assay, or Kulasinghe et al, again using the GeoMx assay. Both studies were performed completely or partially on FFPE samples using assay extension with SARS-CoV-2 probes.
C) High resolution custom spatial assays to capture host-pathogen interactions not in COVID-19 settings, such as the efforts of Lötstedt et all (Biorxiv 2022), who recently performed a similar approach which they termed spatial host-microbiome sequencing (SHM-seq) to capture microbes and host genes in mouse intestine samples or the approach of Saarenpää et al (Giacomello Lab) who employed degenerate probes to capture bacterial and archaeal 16S rRNA hypervariable regions, together with poly-d(T) probes for host mRNA.
There are more, but the above show that a) this development did not take place in a vacuum, while on the other hand capture that even though there are approaches capable to tackle FFPE sample analysis with targeted SARS-CoV-2 probes or 55um resolution methods that work on frozen tissue, there is no method combining the specific characteristics of the presented approach (55um resolution + FFPE support).

Thank you for your suggestion on this point, we agree that additional background information on spatial host-pathogen characterization would be beneficial for readers. We added all the studies mentioned by the reviewer in points A-C to the background section of the manuscript at lines 76-103.

3) The Visium dataset is treated mostly like a single cell dataset in terms of bioinformatic analyses. This is evident in the pipeline followed, in the lack of spatial/Visium-specific analyses (QC approaches, lack of spatially-aware clustering, neighborhood/spatial region calling, spatially-aware cell-cell communication, spot deconvolution, etc.). This is a major missed opportunity from a group specializing in the domain.

We understand the reviewer's concern and to address it we performed spot deconvolution (lines 656-667) utilizing publicly available single-cell data(Delorey et al., 2021) as it allowed us to extract additional information from our ST dataset. To increase the reliability of our results from incorporating the integration of the single cell dataset, we prefiltered the dataset to only include cases within the age and disease duration comparable to the three pathological cases included in our study.

The derived cell type compositions of the ST spots largely confirmed our original, purely ST data-based cluster annotation, and also confirmed the mixed cell type composition of the ST spots. We gained additional insight into the position of less abundant cell types (i.e. NK/T cells, mast cells, etc.) that might have remained hidden without this approach. Of most significance, T/NK cell-dominated spots showed an enrichment and almost equal distribution between the ST clusters 1 and 3 (originally annotated as myeloid- and B cell/plasma cell-enriched, respectively). The results of this analysis are presented in the plots in Supplementary Figure 13 and lines 245-255.

Regarding the general analysis pipeline that we applied: we followed standard practice when handling ST/Visium data in terms of our QC approaches and clustering analysis by utilizing Seurat(Hao et al., 2021) for spatial analysis, as has been performed in many other published studies(Gracia Villacampa et al., 2021; Parigi et al., 2022; Wu et al., 2021) (some use STUtility(Bergenstråhle et al., 2020), a Seurat wrapper package). We also added the original code used for visualization purposes during QC, batch effect removal, and clustering to the GitHub repository (https://github.com/giacomellolab/DualST_Study) for more transparency and reproducibility.

Some approaches listed by the reviewer, while undoubtedly having the potential to expand ST data-based analysis in general, could not be gainfully applied in our sample of choice. The lung sections included in our study were highly homogenous in structure, partly due to sampling peripheral regions of the lungs dominated by alveolar structures, as well as to the general fibrotic transformation and inflammation present in the diseased organs. Spatially aware clustering methods are most useful if there are major distinct structures present in the tissues, however, their implementation can lead to smaller structures (as in smaller vessels, alveoli, and bronchioles, which have more relevance to our study) being completely overlooked, because of which we instead opted for an unbiased clustering approach. While a neighborhood analysis of regions with the presence of SARS-CoV-2 RNA would be of interest, especially for detecting virus-reactive lymphocytes that could have potential clinical relevance, we found the SARS-CoV-2 spots to be dispersed throughout the tissue, as opposed to more-defined viral ‘hot spots’, which made a classical neighborhood analysis less adept for these samples.

4) Since this is a Research manuscript (and not a Method), I would have expected efforts also to integrate the generated data with the spatial and single cell data generated from lung samples from COVID-19 patients as well as efforts to leverage knowledge and observations to generate new knowledge. For the latter, there is mostly a single section and feels rather forced, not as the main topic of the manuscript.

We agree that leveraging previous datasets can generate new insights in the case of our study. To do so, we added a spot deconvolution analysis (lines 245-271 and 656-667) as we described in our previous reply to comment #3. Moreover, taking into account our original goal and the feedback from our reviewers and editor, we now present our work as a Method manuscript, since we want to emphasize the technological development of our spatial host-pathogen transcriptome co-detection, which has various advantages (spatially unbiased detection; whole transcriptome capture of both host and pathogen; 55 µm spatial resolution; FFPE-adapted chemistry) that were not previously combined in any alternative available approaches. Consequently, we shortened the biological interpretation of our findings, while retaining key observations derived from the spot deconvolution analysis and colocalization DE analysis on SARS-CoV-2+ and SARS-CoV-2- spots at lines 245-271, 339-355, and 365-371.

5) Following up on the above, the authors could integrate or compare against the currently available datasets with single cell and spatial data (GeoMx, Visium, RNAScope) from COVID-19 lung samples as well as use the public snRNAseq/scRNASeq samples for deconvolution and label transfer.

We agree that leveraging previous datasets can generate new insight in the case of our study. Besides performing spot deconvolution as suggested by the reviewer (lines 245-271, 339-375, and 656-667), we also took into account the reviewer’s suggestion to incorporate external spatial transcriptome datasets into our analysis. However, unlike for single cell- and Visium-based spatial transcriptome information, there are no readily available, thoroughly validated tools for the integration of GeoMx and Visium data, thus we opted against such an approach and rather used the related studies as references for the biological interpretation of our data. A recent study(Tan et al., 2023) integrated Visium and CODEX data to compare viral high, viral low, and uninfected samples, also leveraging GeoMx data, and RNAScope to identify the SARS-CoV-2 virus. The study presents a promising tool for integrating different modalities, which can advance the spatial field, however, external datasets (from different patients, which can differ in their morphologies and corresponding cell type compositions) were not incorporated for spatial integration. Our technique, by spatially resolving the pathogen transcriptome within the same tissue section using the same platform for host capture (Visium), avoids the challenges of combining information obtained from consecutive sections, where there can be slight spatial shifts in corresponding structural components. On the other hand, integration of various modalities (protein, RNA) from techniques that do not yet allow for co-detection on the same section, can provide a more detailed understanding of the ongoing molecular processes in the tissue, as the above-mentioned study(Tan et al., 2023) clearly demonstrates. This effort, along with others aiming to decipher host-pathogen interactions on a tissue level, could benefit from the high-resolution, transcriptome-wide co-detection of the host and the pathogen mRNA that our technique offers. We mention several of these points in the manuscript in lines 417-421.

6) The authors observed that "SARS-CoV-2+ spots appeared throughout different morphological areas without obvious enrichment in any of the spatial clusters". However, in single cell publications examining SARS-CoV-2 abundance in lung samples from human patients, there is clear enrichment of SARS-CoV-2 in specific cell types (e.g. Delorey et al, Nature 2021). Could the authors comment on these differences?

To assess the interaction between the virus and the host cells in a more nuanced manner, we performed colocalization analysis on the cluster level, first in the original ST clusters, and then in the newly defined deconvolution-based clusters. Deconvolution-based clusters were determined by re-annotating our spatial spots based on the dominant cell type, which resulted in 11 clusters, in line with the number of cell types used for label transfer.

To address the reviewer’s point on cell type specific viral enrichment, we observed that while all 6 ST clusters of the original analysis contained SARS-CoV-2-positive spots, cluster 1 (myeloid-enriched), 3 (B cell/plasma cell-enriched), and 6 (fibroblast-enriched) had a substantially higher number of total viral UMIs, although this difference disappeared when normalized to the number of spots in the cluster. The number of SARS-CoV-2 positive spots in each cluster followed a similar trend, with the immune cell-enriched clusters 1 and 3 having the most spots with viral transcripts detected, although the fibroblast-dominated cluster 6 showed a lower enrichment of viral spots.

Similar analysis of the deconvolution-based clusters revealed a pronounced enrichment of viral UMIs in the T/NK cell-dominated and the B cell/plasma cell-dominated clusters, while the endothelial cell-, myeloid cell- and red blood cell-dominated clusters showed a moderate enrichment of viral transcripts, both on the absolute and per spot level. Interestingly, here the fibroblast-dominated cluster showed the second lowest absolute and the lowest per spot viral transcript content, which suggests that in the fibroblast-enriched ST cluster, the SARS-CoV-2 RNA might often originate from another cell type, masked by the gene signature of activated fibroblasts. The number of SARS-CoV-2-positive spots per cluster followed a similar pattern. Of note, epithelial and ciliated cells (described as major targets of the virus) showed very low levels of viral transcripts, likely due to the analyzed cases being of late disease phase. Overall, the relative abundance of SARS-CoV-2 RNAs in the myeloid and endothelial cell clusters is in line with the observations of the study of Delorey et al.(Delorey et al., 2021), while the strong enrichment of viral transcripts in the lymphocyte-dominated clusters (especially the T/NK cluster) most likely represents, at least in part, these cells’ interaction with other infected cell types as part of the adaptive immune response. The results of this analysis are presented in the plots in Supplementary Figure 14 and lines 256-271.

7) The comparison of SARS-CoV-2+ and SARS-CoV-2- spot can be a bit more nuanced than as approached, since viral abundance can differ across architectural components or cell types. Could the analysis be repeated by matching the regions compared (e.g. alveoli vs alveoli, bronchial epithelium vs bronchial epithelium), cluster types (if possible), as well as cellular proportions? Since the comparisons could be confounded by the differences in cellular proportions, and lead to performing a DE between cell populations.

To address the reviewer’s point on conducting colocalization analysis within cluster types and cell types, we repeated the colocalization analysis and listed the DE genes between SARS-CoV-2+ and SARS-CoV-2- spots on an ST cluster level. The highest-ranking DE genes were shared across the ST clusters, which also largely overlapped with the results of our earlier analysis, performed without taking the cellular source of viral transcripts into account. We also generated a list of unique DE genes in all analyzed deconvolution-based clusters, to allow for revealing cell type-specific responses to the presence of the virus. In the B-cell dominated deconvolution-based clusters, we found two immunoglobulin variable genes (IGLV9-49 and IGHV1-46) upregulated in the SARS-CoV-2 positive spots, which, along with the IGKV4-1 transcript that we found to be enriched in the viral spots in our en masse analysis, can potentially indicate virus-reactive B cell clones in close proximity to infected cells. We also found differential expression of known SARS-CoV-2 entry factors between the SARS-CoV-2+ and SARS-CoV-2- spots in a number of clusters, however, some of these were specific to a single cluster: we found MMRN1 to be enriched in the SARS-CoV-2+ spots of the deconvolution-based endothelial cluster, while SARS-CoV-2+ B cell/plasma cell-dominated spots showed downregulation of EMILIN1 and upregulation of NRP1. While the biological interpretation of these differences is beyond the scope of our paper, they show the potential of our approach to detect cell type-specific differences in the presence of the virus. Therefore, we provide the results of this analysis in Supplementary Tables 9-10 of the manuscript and lines 339-355.

Unfortunately, the samples included in our study were not optimal for comparison of microscopically annotated regions, as suggested by the reviewer, since the tissues were highly homogenous, showing extensive fibrosis and leukocyte infiltration, and were scarce in major, easily distinguishable structural components. We agree that such an approach has the potential to provide a more nuanced analysis, but for the listed reasons we could not perform it in a meaningful manner in our experimental material. However, we would like to point out that our technology can accommodate such an approach, which, for example, can be advantageous in the analysis of lung samples of earlier disease phases, with more preserved tissue structure.

8) The presence of SARS-CoV-2 reads in the myeloid compartment could potentially point to phagocytosis as opposed to infection (e.g. as potentially observed in the epithelial compartments). Could the authors comment and expand?

Previous works described professional phagocytes (monocytes, macrophages and dendritic cells) as able to internalize SARS-CoV-2 viral particles directly through membrane receptors similar to other cell types, or through the classical, Fc gamma receptor-mediated endocytic way(Matveeva et al., 2022). Independent from the mechanism, viral entry into the immune cells most often leads to an abortive infection, causing apoptosis (pyroptosis) of the cell and release of inflammatory mediators. Alternative outcomes, such as productive, or even latent infection of the phagocytes are also possible, however, they are likely less frequent and their biological relevance is still unclear. Of note, B cells, as non-professional phagocytes, might also internalize SARS-CoV-2 particles through phagocytosis(Matveeva et al., 2022).

In our dual transcriptome detection, we capture all forms of viral transcripts that have intact RNA sequences complementary to our probe sets. Consequently, just based on the captured viral RNA, we are not able to distinguish between different entry pathways of the SARS-CoV-2 or the outcome of the uptake of the virus.

9) The authors mention a set of suggested genes: SRRM2, HNRNPA2/B1, SMAP2, ARHGEF1, and TCIRG1 as "plausible novel targets for molecular interventions aiming to block COVID-19 pathogenesis in the human lungs". Could they expand on why they consider them to be plausible targets and how they envision the function of targeted therapeutics? To my understanding, the main supporting data is that these targets were differentially expressed in the SARS-CoV-2 RNA+ spots.

In our colocalization analysis and the subsequent DE gene analysis, we identified several genes to be differentially expressed between SARS-CoV-2+ and SARS-CoV-2- tissue spots, and previous studies, referenced between lines 309-355 of the manuscript, described many of these genes in COVID-19 pathogenesis. As opposed to these genes, previously only in silico analysis or cell culture-based experiments supported the potential involvement of SRRM2, HNRNPA2/B1, SMAP2, ARHGEF1, and TCIRG1 in SARS-CoV-2 infection, however, supporting data from in situ studies in the spatial context of diseased human lungs was lacking. Upon more nuanced colocalization analysis performed on the cluster level, we found these genes to be upregulated across all ST and four out of five analyzed deconvolution-based clusters (B/plasma cell, endothelial, myeloid, and RBC), suggesting their general role in the metabolism of viral components in infected cells, likely independent of cell type. In the T/NK cell-dominated cluster we did not observe a similar enrichment of these genes in the SARS-CoV-2+ spots, likely due to these cells not being the primary target of the virus, but rather interacting with the infected cells as part of the cellular immune response. As discussed in the manuscript at lines 356-375 and 428-432, others described the protein products of these genes as playing a role in RNA processing or vesicular transport, both processes central to the replication and spread of SARS-CoV-2, in line with their ubiquitous upregulation in SARS-CoV-2+ spots in our samples. There are also a number of studies available in support of ARHGEF1(Jha et al., 2022) or the vacuolar ATPase complex (involving TCIRG1)(Icho et al., 2022; Stefanik et al., 2021) being potential drug targets in COVID-19 or other viral infections such as influenza(Hu et al., 2018). Of note, the exploration of several inhibitors of the hnRNP A/B proteins in cancer therapy is ongoing(Lu et al., 2022), which could also potentially be tested in the context of SARS-CoV-2 infection. Taking all this into account, these genes might indeed deserve attention as potentially relevant modulators of viral metabolism and serve for targeted therapeutics, however, it is beyond the scope of our study to further validate them in this context, thus we decided to tone down our statement related to their pharmacological targeting in the manuscript.

10) The analysis is performed en bloc with all the SARS-CoV-2+ spots, regardless of the cluster type. I would be very cautious lumping them all together, since some (as mentioned above) could be through phagocytosis or ambient RNA. There is also RNA transfer across spots which was also not evaluated during the QC (as was mentioned the QC was similar to that followed for single cell data).

In response to the reviewer’s concern on the transfer of RNA across spots, while an increasing number of tools for ambient RNA decontamination are currently being implemented in scRNAseq analysis pipelines, this type of contamination is relevant in droplet-based sequencing strategies, and thus is of no concern during the preparation of sequencing-ready libraries on a Visium slide. To our knowledge, there is no generally accepted method available to mitigate the effect of potential RNA bleed-through (also referred to as ‘spot swapping’ in a recent paper proposing a tool for such corrections(Ni et al., 2022)) between spots on ST data generation, thus we did not implement such a quality control step in our analysis. For the Visium FFPE assay(10X Genomics. Visium Spatial Gene Expression Reagent Kits for FFPE User Guide. Document Number CG000407, Rev A. (2021).CG000407_VisiumSpatialGeneExpressionforFFPE_UserGuide_RevA.pdf, n.d.) used in the study, a standard tissue permeabilization time is used across human and mouse tissues to minimize RNA transfer across spots, as 10X Genomics tested this on many different tissue types, including lung(10X Genomics. Experimental Planning and Design - Official 10x Genomics Support, n.d.).

11) The authors mention that SRRM2 could be a substrate for SARS-CoV-2. Do the same spots co-express other known factors/co-factors for SARS-CoV-2? In published studies of tissue data from deceased/severe patients, there is usually low expression of the molecules known or hypothesized to be factors/co-factors for SARS-CoV-2 entry, such as ACE2 or TMPRSS2 in the SARS-CoV-2 RNA+ cells. Therefore, detecting a gene up-regulated in the (+) sub-fraction, doesn't automatically make it a potential case for an entry or interaction molecule.

We agree with the reviewer that the upregulation of SRRM2 in the SARS-CoV-2+ tissue spots does not automatically mean that we can consider SRRM2 an entry factor.

Upon the reviewer’s request, we analyzed on a cluster level the expression levels of known SARS-CoV-2 entry factors in the subset of SARS-CoV-2 spots also expressing SRRM2. We found strong co-expression of EMILIN2, CTSB, and CTSL in the myeloid cell-dominated clusters, and of EMILIN1 in the fibroblast-dominated clusters. In line with the reviewer’s comment, however, we detected no upregulation of the membrane receptor ACE2 or NRP1 in these clusters. In fact, ACE2 showed minimal expression in the analyzed spots, while NPR1 showed moderate expression levels in a number of clusters. In addition, PCSK3, DPP4, and PCK5 were not expressed in these SRRM2+/SARS-CoV-2+ spots. These observations, along with other studies, make it likely that the expression of these factors is not regulated in a coordinated manner. The results of this analysis are presented in the plots in Additional Figure 2.

Based on the results discussed above, we think that the enrichment of the SRRM2 gene in the viral spots is a reactive response from the host cells to the viral infection, rather than a prerequisite that allows for the effective uptake of the virus. This interpretation is supported by the fact that most known SARS-CoV-2 entry factors are either cellular receptors (ACE2, NRP1) that directly bind the virus on the cell membrane, or proteases (TMPRSS2, PCSK3, CTSB, CTSL, EMILIN1, EMILIN2, MMRN1, MMRN2, DPP4) that facilitate viral entry by site-specific cleavage of surface protein components of the virion, while SRRM2 is involved in RNA processing, presumably both for the host and viral transcripts.

12) The authors mention "our in situ spatial analysis provides the first confirmation of these expressional changes to be directly associated to cells actively expressing viral components in the COVID-19 diseased human lungs". The resolution of the assay utilized does not enable this conclusion since in the same spot there can be a collection of different cells (5+ for the lung). It's impossible to identify if the signal is coming directly from a specific cell, unless a complementary assay is performed that permits single cell resolution. Alternatively, the authors could use publicly available single cell (nucleus) data from lung samples of COVID-19 patients to show that indeed these expression differences take place in the SARS-CoV-2 RNA+ subfraction when compared to cells from the same cluster/population without SARS-CoV-2 RNA presence.

As the reviewer pointed out, the Visium technology at this moment does not reach single-cell resolution, and consequently, the transcripts detected at every spot represent a mixture of 1-10 cells. Our re-performed colocalization analysis on the cluster level confirmed a general upregulation of all five discussed genes (SRRM2, HNRNPA2/B1, TCIRG1, SMAP2, ARHGEF1) across all ST and four of the five analyzed deconvolution-based spot clusters, as discussed in our reply to Comment #9. This provides further evidence that the increased number of transcripts in the SARS-CoV-2+ spots likely originate from actively infected cells, and not their neighbors.

With the sentence quoted above, we tried to emphasize that we observed a localized upregulation of the listed genes in tissue spots with the active presence of the SARS-CoV-2 virus, unlike in other studies, where some of these genes were found to be enriched in bulk lung RNA from COVID-19 patients vs. healthy controls. These latter works were not adept to discern if the upregulation of these genes is a direct or indirect response to the presence of the virus. Based on our results, the observed enrichment of these transcripts either originates from cells expressing SARS-CoV-2 RNA or their direct neighbors, thus it is both spatially and temporally coordinated with the presence of viral RNA, which is in good agreement with the involvement of these genes in RNA processing and vesicular transport pathways.

Minor Comments

13) The high variability of the expression levels observed in the M, E, S, ORF1ab, ORF3a, ORF7a, ORF7b, and ORF8 genes was observed also across the SARS-CoV-2+ spots within the same slide or the overall ranking was consistent per slide but differed mostly across slides?

The histogram in Figure 3b shows the variability of each SARS-CoV-2 gene summed across all samples from all slides. In addition, Supplementary Figure 9 shows the distribution of the different SARS-CoV-2 genes by sample section and by the gene. The overall ranking appears largely consistent across sample sections, which were distributed across different slides.

14) P-values reported: many p values are reported as < 2.2 * 10-16. This is a common issue dictated by technical reasons (machine epsilon) from the programming language or statistical package used. Can the authors substitute with the actual p-values observed? Could they also add a mention of the statistical test performed in the text and figures as well as the p-values when describing the outcome of statistical test?

In line with the reviewer’s comment, we altered how we present the p-values in the Methods text and figure legends in the manuscripts, opting for reporting that p-values were less than the significance threshold of 0.05 (p-value < 0.05) (Figure 2, Figure 4, Figure 5, Supplementary Figure 8). We also added the statistical tests and p-values to the text and figure legends for those used for:
the Pearson correlations (Figure 2, Supplementary Figure 8),
the differential expression analysis identifying cluster-specific DE genes and cluster 5 subcluster-related DE genes (differential expression analysis used Wilcoxon rank sum test and “bimod” Likelihood-ratio tests, p < 0.05) (Figure 4, Supplementary Figure 12, Supplementary Tables 4-5),
the differential expression analysis comparing COVID-19 and control lung sections (differential expression analysis used Wilcoxon rank sum test and “bimod” Likelihood-ratio tests, p < 0.05) (Figure 4, Supplementary Table 6),
differential expression analysis comparing COVID-19 and control lung sections within clusters (differential expression analysis used Wilcoxon rank sum test and “bimod” Likelihood-ratio tests, p < 0.05) (Supplementary Table 7),
differential expression analysis for SARS-CoV-2+ and SARS-CoV-2- spots in COVID-19 sections, within ST clusters, and within deconvolution-based clusters (differential expression analysis used Wilcoxon rank sum test and DESeq2 negative binomial distribution tests, p-value < 0.05) (Figure 5, Supplementary Tables 8-10).

15) When working with pathogen RNA in single cell or spatial, you want to ascertain that you have a bona fide event. Some helpful statistics could include: a) the co-occurrence of expressed viral genes in the same spots (pairwise and in larger combinations), b) the spots where a singleton viral gene expression event occurs, c) correlation of SARS-CoV-2 gene expression levels (UMIs) vs the average UMI counts per spot in a slide. Could these statistics be added and plotted?

We thank the reviewer for raising this important point. To visualize the spots where a singleton viral gene expression event occurs, as mentioned by the reviewer in point b), we plotted the number of singleton spots for each SARS-CoV-2 gene in Additional Figure 1a. In addition, we present the total number of spots where 1, 2, 3, 4, 5, 6, 7, 8, or 9 different SARS-CoV-2 genes are detected (Additional Figure 1b).

To visualize the co-occurrence of expressed viral genes in the same spot, mentioned by the reviewer in point a), we looked into two aspects. First, for each SARS-CoV-2 gene, we looked into the set of spots where that gene is detected and how many other SARS-CoV-2 genes are detected there (Additional Figure 1c). This is visualized as both the number of spots each SARS-CoV-2 gene is detected in (left panels) and the total UMIs of each SARS-CoV-2 gene (right panels).

We found that the number of singleton spots in our dataset (Additional Figure 1b) varies per SARS-CoV-2 gene (Additional Figure 1a). Of note, these levels largely follow the abundance of detected SARS-CoV-2 genes shown in Figure 3 of the main manuscript. In addition, as discussed in the main manuscript text at lines 205-220 and 422-423, these levels also largely correspond to the order of genes from the 3’ end of the viral genomic RNA (gRNA).

To address point c) raised by the reviewer on the correlation of SARS-CoV-2 gene expression levels (UMIs) vs the average UMI counts per spot in a slide, we show the correlation of SARS-CoV-2 gene expression (as UMI counts) in a spot to the average SARS-CoV-2 expression in a spot and observe a high correlation of r= 0.96, p < 0.05 (Additional Figure 3a). In addition, we observed a lack of correlation between SARS-CoV-2 UMI counts and human average UMIs per spot (r=0.06, p < 0.05) (Additional Figure 3b), supporting that the presence of viral RNA does not affect the overall RNA output of the host cells and that our viral probes are specific and do not detect human genes.

16) The color scales in some graphs (e.g. Figure 4d, e) are not the same, leading to a different meaning for each color.

Thank you for pointing out this discrepancy. Now we have updated Figure 4d and 4e of the manuscript with the same color scale, and also present the updated graphs below.

17) Fig 5b: I find the colocalization plots quite challenging as implemented to show the co-occurrence of SARS-CoV-2 RNA and of the relevant gene. For instance, it's very difficult to discern differences (at least for me) between + and - for SRRM2 and potentially CXCL9. Could the color scale or a two-axis color gradient (viral RNA abundance on the X axis and UMIs on the Y) be more helpful?

We thank the reviewer for pointing out this problem with our visualization of DE gene hits in our colocalization analysis. Since the plot was difficult to interpret, we decided to exclude the SRRM2 panel from Figure 5, and we also changed the related paragraphs of the manuscript. Since we performed our analysis between binary, SARS-CoV-2+ versus SARS-CoV-2-, spots and did not consider the abundance of viral transcripts per spot, the two-axis color gradient suggested by the reviewer (with viral RNA abundance on the x-axis and UMIs on the y-axis) would not be in line with our approach. We tried many different color schemes for visualizing the expression between SARS-CoV-2+ and SARS-CoV-2- spots and found the colors visualized below and in Figure 5b of the main manuscript to work the best.

18) Line 446: There is an abbreviation of ORF10 as 10x Genomics

Thank you for highlighting this ambiguity. This is not an abbreviation of ORF10, but to show that the probes are obtained from 10X Genomics. 10X Genomics was moved to the end of the sentence to make this distinction clearer (now at line 498).

19) Novelty Score: The term isn't widely used. The formula should be added for the log10 UMI/gene ratio calculation for reference.

We added the formula where the Novelty Score is mentioned under “Quality Control” in the Methods section at lines 621-625. We added the clarification: “An additional spot filter was applied where a novelty score for each spot was calculated by taking the log transform of the ratio of the total genes detected divided by the total UMI counts detected in the same spot (Formula: log10(nFeature_RNA) / log10(nCount_RNA)). Spots with this score greater than 0.87 were kept”.

20) Registration: Adobe photoshop was used for image registration. How was registration quality evaluated? Would an elastic registration that can tackle tissue deformations from sectioning be preferable?

We thank the reviewer for the suggestion. As part of our newly added quantitative validation pipeline, we achieved an elastic registration by displaying the RNAscope section pixel colors adjusted to the positions of ST section pixels. Specifically, sections of sample 1C, analyzed in the different RNA detection assays (ST, RNAScope, ISS), were registered elastically using conformal mapping. First, four corner points were manually selected on each tissue section, then they were conformally mapped onto a square with the chosen corners adjusted to the vertices. Since all images were mapped onto the same square, one-to-one correspondence between ST, RNAScope and ISS sections could be established. The positions of aligned RNAScope section pixels were transferred from the position of the corresponding pixels in the ST section via the aforementioned one-to-one correspondence. We added this information in the Methods section at lines 668-679.

References
10X Genomics. Experimental planning and design - official 10x genomics support. (n.d.). Retrieved April 3, 2023, from https://www.10xgenomics.com/support/spatial-gene-expression-ffpe/documentation/workflows/ffpe-v-1/steps/experimental-design-and-planning/visium-spatial-gene-expression-for-ffpe-tested-tissues
10X Genomics. Visium Spatial Gene Expression Reagent Kits for FFPE User Guide. Document Number CG000407, Rev A. (2021).CG000407_VisiumSpatialGeneExpressionforFFPE_UserGuide_RevA.pdf. (n.d.).
Bergenstråhle, J., Larsson, L., & Lundeberg, J. (2020). Seamless integration of image and molecular analysis for spatial transcriptomics workflows. BMC Genomics, 21(1), 482. https://doi.org/10.1186/s12864-020-06832-3
Bost, P., Giladi, A., Liu, Y., Bendjelal, Y., Xu, G., David, E., Blecher-Gonen, R., Cohen, M., Medaglia, C., Li, H., Deczkowska, A., Zhang, S., Schwikowski, B., Zhang, Z., & Amit, I. (2020). Host-Viral Infection Maps Reveal Signatures of Severe COVID-19 Patients. Cell, 181(7), 1475–1488.e12. https://doi.org/10.1016/j.cell.2020.05.006
Butler, D., Mozsary, C., Meydan, C., Foox, J., Rosiene, J., Shaiber, A., Danko, D., Afshinnekoo, E., MacKay, M., Sedlazeck, F. J., Ivanov, N. A., Sierra, M., Pohle, D., Zietz, M., Gisladottir, U., Ramlall, V., Sholle, E. T., Schenck, E. J., Westover, C. D., … Mason, C. E. (2021). Shotgun transcriptome, spatial omics, and isothermal profiling of SARS-CoV-2 infection reveals unique host responses, viral diversification, and drug interactions. Nature Communications, 12(1), 1660. https://doi.org/10.1038/s41467-021-21361-7
Da Costa, C. B. P., Cruz, A. C. D. M., Penha, J. C. Q., Castro, H. C., Da Cunha, L. E. R., Ratcliffe, N. A., Cisne, R., & Martins, F. J. (2022). Using in vivo animal models for studying SARS-CoV-2. Expert Opinion on Drug Discovery, 17(2), 121–137. https://doi.org/10.1080/17460441.2022.1995352
Dardani, I., Emert, B. L., Goyal, Y., Jiang, C. L., Kaur, A., Lee, J., Rouhanifard, S. H., Alicea, G. M., Fane, M. E., Xiao, M., Herlyn, M., Weeraratna, A. T., & Raj, A. (2022). ClampFISH 2.0 enables rapid, scalable amplified RNA detection in situ. Nature Methods, 19(11), 1403–1410. https://doi.org/10.1038/s41592-022-01653-6
Delorey, T. M., Ziegler, C. G. K., Heimberg, G., Normand, R., Yang, Y., Segerstolpe, Å., Abbondanza, D., Fleming, S. J., Subramanian, A., Montoro, D. T., Jagadeesh, K. A., Dey, K. K., Sen, P., Slyper, M., Pita-Juárez, Y. H., Phillips, D., Biermann, J., Bloom-Ackermann, Z., Barkas, N., … Regev, A. (2021). COVID-19 tissue atlases reveal SARS-CoV-2 pathology and cellular targets. Nature, 595(7865), 107–113. https://doi.org/10.1038/s41586-021-03570-8
Desai, N., Neyaz, A., Szabolcs, A., Shih, A. R., Chen, J. H., Thapar, V., Nieman, L. T., Solovyov, A., Mehta, A., Lieb, D. J., Kulkarni, A. S., Jaicks, C., Xu, K. H., Raabe, M. J., Pinto, C. J., Juric, D., Chebib, I., Colvin, R. B., Kim, A. Y., … Deshpande, V. (2020). Temporal and spatial heterogeneity of host response to SARS-CoV-2 pulmonary infection. Nature Communications, 11(1), 6319. https://doi.org/10.1038/s41467-020-20139-7
Engblom, C., Thrane, K., Lin, Q., Andersson, A., Toosi, H., Chen, X., Steiner, E., Mantovani, G., Hagemann-Jensen, M., Saarenpää, S., Jangard, M., Michaëlsson, J., Hartman, J., Lagergren, J., Mold, J., Lundeberg, J., & Frisén, J. (2022). Spatial transcriptomics of T and B cell receptors uncovers lymphocyte clonal dynamics in human tissue. In bioRxiv (p. 2022.11.22.516865). https://doi.org/10.1101/2022.11.22.516865
Fang, R., Xia, C., Close, J. L., Zhang, M., He, J., Huang, Z., Halpern, A. R., Long, B., Miller, J. A., Lein, E. S., & Zhuang, X. (2022). Conservation and divergence of cortical cell organization in human and mouse revealed by MERFISH. Science, 377(6601), 56–62. https://doi.org/10.1126/science.abm1741
Gracia Villacampa, E., Larsson, L., Mirzazadeh, R., Kvastad, L., Andersson, A., Mollbrink, A., Kokaraki, G., Monteil, V., Schultz, N., Appelberg, K. S., Montserrat, N., Zhang, H., Penninger, J. M., Miesbach, W., Mirazimi, A., Carlson, J., & Lundeberg, J. (2021). Genome-wide spatial expression profiling in formalin-fixed tissues. Cell Genomics, 1(3), 100065. https://doi.org/10.1016/j.xgen.2021.100065
Grellet, E., L’Hôte, I., Goulet, A., & Imbert, I. (2022). Replication of the coronavirus genome: A paradox among positive-strand RNA viruses. The Journal of Biological Chemistry, 298(5), 101923. https://doi.org/10.1016/j.jbc.2022.101923
Gyllborg, D., Langseth, C. M., Qian, X., Choi, E., Salas, S. M., Hilscher, M. M., Lein, E. S., & Nilsson, M. (2020). Hybridization-based in situ sequencing (HybISS) for spatially resolved transcriptomics in human and mouse brain tissue. Nucleic Acids Research, 48(19), e112. https://doi.org/10.1093/nar/gkaa792
Hamdane, N., Jühling, F., Crouchet, E., El Saghire, H., Thumann, C., Oudot, M. A., Bandiera, S., Saviano, A., Ponsolles, C., Roca Suarez, A. A., Li, S., Fujiwara, N., Ono, A., Davidson, I., Bardeesy, N., Schmidl, C., Bock, C., Schuster, C., Lupberger, J., … Baumert, T. F. (2019). HCV-Induced Epigenetic Changes Associated With Liver Cancer Risk Persist After Sustained Virologic Response. Gastroenterology, 156(8), 2313–2329.e7. https://doi.org/10.1053/j.gastro.2019.02.038
Hao, Y., Hao, S., Andersen-Nissen, E., Mauck, W. M., 3rd, Zheng, S., Butler, A., Lee, M. J., Wilk, A. J., Darby, C., Zager, M., Hoffman, P., Stoeckius, M., Papalexi, E., Mimitou, E. P., Jain, J., Srivastava, A., Stuart, T., Fleming, L. M., Yeung, B., … Satija, R. (2021). Integrated analysis of multimodal single-cell data. Cell, 184(13), 3573–3587.e29. https://doi.org/10.1016/j.cell.2021.04.048
He, S., Bhatt, R., Brown, C., Brown, E. A., Buhr, D. L., Chantranuvatana, K., Danaher, P., Dunaway, D., Garrison, R. G., Geiss, G., Gregory, M. T., Hoang, M. L., Khafizov, R., Killingbeck, E. E., Kim, D., Kim, T. K., Kim, Y., Klock, A., Korukonda, M., … Beechem, J. M. (2022). High-plex imaging of RNA and proteins at subcellular resolution in fixed tissue by spatial molecular imaging. Nature Biotechnology, 40(12), 1794–1806. https://doi.org/10.1038/s41587-022-01483-z
Hu, C.-M. J., Chen, Y.-T., Fang, Z.-S., Chang, W.-S., & Chen, H.-W. (2018). Antiviral efficacy of nanoparticulate vacuolar ATPase inhibitors against influenza virus infection. International Journal of Nanomedicine, 13, 8579–8593. https://doi.org/10.2147/IJN.S185806
Icho, S., Rujas, E., Muthuraman, K., Tam, J., Liang, H., Landreth, S., Liao, M., Falzarano, D., Julien, J.-P., & Melnyk, R. A. (2022). Dual Inhibition of Vacuolar-ATPase and TMPRSS2 Is Required for Complete Blockade of SARS-CoV-2 Entry into Cells. Antimicrobial Agents and Chemotherapy, 66(7), e0043922. https://doi.org/10.1128/aac.00439-22
Illumina. Evaluating RNA Quality from FFPE Samples. Technical Note: RNA Sequencing. https://emea.illumina.com/content/dam/illumina-marketing/documents/products/technotes/evaluating-rna-quality-from-ffpe-samples-technical-note-470-2014-001.pdf. (n.d.).
In Situ gene expression. (n.d.). 10x Genomics. Retrieved January 25, 2023, from https://www.10xgenomics.com/products/in-situ-gene-expression
Janesick, A., Shelansky, R., Gottscho, A. D., Wagner, F., Rouault, M., Beliakoff, G., de Oliveira, M. F., Kohlway, A., Abousoud, J., Morrison, C. A., Drennon, T. Y., Mohabbat, S. H., Williams, S. R., & Taylor, S. E. B. (2022). High resolution mapping of the breast cancer tumor microenvironment using integrated single cell, spatial and in situ analysis of FFPE tissue. In bioRxiv (p. 2022.10.06.510405). https://doi.org/10.1101/2022.10.06.510405
Jha, P., Singh, P., Arora, S., Sultan, A., Nayek, A., Ponnusamy, K., Syed, M. A., Dohare, R., & Chopra, M. (2022). Integrative multiomics and in silico analysis revealed the role of ARHGEF1 and its screened antagonist in mild and severe COVID-19 patients. Journal of Cellular Biochemistry, 123(3), 673–690. https://doi.org/10.1002/jcb.30213
Kim, D., Lee, J.-Y., Yang, J.-S., Kim, J. W., Kim, V. N., & Chang, H. (2020). The Architecture of SARS-CoV-2 Transcriptome. Cell, 181(4), 914–921.e10. https://doi.org/10.1016/j.cell.2020.04.011
Kulasinghe, A., Tan, C. W., Dos Santos Miggiolaro, A. F. R., Monkman, J., SadeghiRad, H., Bhuva, D. D., da Silva Motta Junior, J., Vaz de Paula, C. B., Nagashima, S., Baena, C. P., Souza-Fonseca-Guimaraes, P., de Noronha, L., McCulloch, T., Rodrigues Rossi, G., Cooper, C., Tang, B., Short, K. R., Davis, M. J., Souza-Fonseca-Guimaraes, F., … O’Byrne, K. (2021). Profiling of lung SARS-CoV-2 and influenza virus infection dissects virus-specific host responses and gene signatures. The European Respiratory Journal: Official Journal of the European Society for Clinical Respiratory Physiology. https://doi.org/10.1183/13993003.01881-2021
Lamers, M. M., & Haagmans, B. L. (2022). SARS-CoV-2 pathogenesis. Nature Reviews. Microbiology, 20(5), 270–284. https://doi.org/10.1038/s41579-022-00713-0
Long, S. (2021). SARS-CoV-2 Subgenomic RNAs: Characterization, Utility, and Perspectives. Viruses, 13(10). https://doi.org/10.3390/v13101923
Lu, Y., Wang, X., Gu, Q., Wang, J., Sui, Y., Wu, J., & Feng, J. (2022). Heterogeneous nuclear ribonucleoprotein A/B: an emerging group of cancer biomarkers and therapeutic targets. Cell Death Discovery, 8(1), 337. https://doi.org/10.1038/s41420-022-01129-8
Margaroli, C., Benson, P., Sharma, N. S., Madison, M. C., Robison, S. W., Arora, N., Ton, K., Liang, Y., Zhang, L., Patel, R. P., & Gaggar, A. (2021). Spatial mapping of SARS-CoV-2 and H1N1 lung injury identifies differential transcriptional signatures. Cell Reports. Medicine, 2(4), 100242. https://doi.org/10.1016/j.xcrm.2021.100242
Matsunaga, H., Arikawa, K., Yamazaki, M., Wagatsuma, R., Ide, K., Samuel, A. Z., Takamochi, K., Suzuki, K., Hayashi, T., Hosokawa, M., Kambara, H., & Takeyama, H. (2022). Reproducible and sensitive micro-tissue RNA sequencing from formalin-fixed paraffin-embedded tissues for spatial gene expression analysis. Scientific Reports, 12(1), 19511. https://doi.org/10.1038/s41598-022-23651-6
Matveeva, O., Nechipurenko, Y., Lagutkin, D., Yegorov, Y. E., & Kzhyshkowska, J. (2022). SARS-CoV-2 infection of phagocytic immune cells and COVID-19 pathology: Antibody-dependent as well as independent cell entry. Frontiers in Immunology, 13, 1050478. https://doi.org/10.3389/fimmu.2022.1050478
Melms, J. C., Biermann, J., Huang, H., Wang, Y., Nair, A., Tagore, S., Katsyv, I., Rendeiro, A. F., Amin, A. D., Schapiro, D., Frangieh, C. J., Luoma, A. M., Filliol, A., Fang, Y., Ravichandran, H., Clausi, M. G., Alba, G. A., Rogava, M., Chen, S. W., … Izar, B. (2021). A molecular single-cell lung atlas of lethal COVID-19. Nature, 595(7865), 114–119. https://doi.org/10.1038/s41586-021-03569-1
Merritt, C. R., Ong, G. T., Church, S. E., Barker, K., Danaher, P., Geiss, G., Hoang, M., Jung, J., Liang, Y., McKay-Fleisch, J., Nguyen, K., Norgaard, Z., Sorg, K., Sprague, I., Warren, C., Warren, S., Webster, P. J., Zhou, Z., Zollinger, D. R., … Beechem, J. M. (2020). Multiplex digital spatial profiling of proteins and RNA in fixed tissue. Nature Biotechnology, 38(5), 586–599. https://doi.org/10.1038/s41587-020-0472-9
Ni, Z., Prasad, A., Chen, S., Halberg, R. B., Arkin, L. M., Drolet, B. A., Newton, M. A., & Kendziorski, C. (2022). SpotClean adjusts for spot swapping in spatial transcriptomics data. Nature Communications, 13(1), 2971. https://doi.org/10.1038/s41467-022-30587-y
Oranger, A., Manzari, C., Chiara, M., Notario, E., Fosso, B., Parisi, A., Bianco, A., Iacobellis, M., d’Avenia, M., D’Erchia, A. M., & Pesole, G. (2021). Accurate detection and quantification of SARS-CoV-2 genomic and subgenomic mRNAs by ddPCR and meta-transcriptomics analysis. Communications Biology, 4(1), 1215. https://doi.org/10.1038/s42003-021-02748-0
Parigi, S. M., Larsson, L., Das, S., Ramirez Flores, R. O., Frede, A., Tripathi, K. P., Diaz, O. E., Selin, K., Morales, R. A., Luo, X., Monasterio, G., Engblom, C., Gagliani, N., Saez-Rodriguez, J., Lundeberg, J., & Villablanca, E. J. (2022). The spatial transcriptomic landscape of the healing mouse intestine following damage. Nature Communications, 13(1), 828. https://doi.org/10.1038/s41467-022-28497-0
Park, J., Foox, J., Hether, T., Danko, D. C., Warren, S., Kim, Y., Reeves, J., Butler, D. J., Mozsary, C., Rosiene, J., Shaiber, A., Afshin, E. E., MacKay, M., Rendeiro, A. F., Bram, Y., Chandar, V., Geiger, H., Craney, A., Velu, P., … Mason, C. E. (2022). System-wide transcriptome damage and tissue identity loss in COVID-19 patients. Cell Reports. Medicine, 3(2), 100522. https://doi.org/10.1016/j.xcrm.2022.100522
Rendeiro, A. F., Ravichandran, H., Bram, Y., Chandar, V., Kim, J., Meydan, C., Park, J., Foox, J., Hether, T., Warren, S., Kim, Y., Reeves, J., Salvatore, S., Mason, C. E., Swanson, E. C., Borczuk, A. C., Elemento, O., & Schwartz, R. E. (2021). The spatial landscape of lung pathology during COVID-19 progression. Nature, 593(7860), 564–569. https://doi.org/10.1038/s41586-021-03475-6
Ren, X., Wen, W., Fan, X., Hou, W., Su, B., Cai, P., Li, J., Liu, Y., Tang, F., Zhang, F., Yang, Y., He, J., Ma, W., He, J., Wang, P., Cao, Q., Chen, F., Chen, Y., Cheng, X., … Zhang, Z. (2021). COVID-19 immune features revealed by a large-scale single-cell transcriptome atlas. Cell, 184(7), 1895–1913.e19. https://doi.org/10.1016/j.cell.2021.01.053
RNAscope ISH Technology. (n.d.). Retrieved April 3, 2023, from https://www.bio-techne.com/reagents/rnascope-ish-technology
Saberi, A., Gulyaeva, A. A., Brubacher, J. L., Newmark, P. A., & Gorbalenya, A. E. (2018). A planarian nidovirus expands the limits of RNA genome size. PLoS Pathogens, 14(11), e1007314. https://doi.org/10.1371/journal.ppat.1007314
Stefanik, M., Strakova, P., Haviernik, J., Miller, A. D., Ruzek, D., & Eyer, L. (2021). Antiviral Activity of Vacuolar ATPase Blocker Diphyllin against SARS-CoV-2. Microorganisms, 9(3). https://doi.org/10.3390/microorganisms9030471
Tang, X., Chen, J., Zhang, X., Liu, X., Xie, Z., Wei, K., Qiu, J., Ma, W., Lin, C., & Ke, R. (2022). Improved in situ sequencing for high-resolution targeted spatial transcriptomic analysis in tissue sections. In bioRxiv (p. 2022.10.16.512401). https://doi.org/10.1101/2022.10.16.512401
Tang, Z., Zhang, T., Yang, B., Su, J., & Song, Q. (2023). spaCI: deciphering spatial cellular communications through adaptive graph model. Briefings in Bioinformatics, 24(1). https://doi.org/10.1093/bib/bbac563
Tan, X., Grice, L. F., Tran, M., Mulay, O., Monkman, J., Blick, T., Vo, T., Almeida, A. C. S., da Silva Motta Junior, J., de Moura, K. F., Machado-Souza, C., Souza-Fonseca-Guimaraes, P., Baena, C. P., de Noronha, L., Guimaraes, F. S. F., Luu, H. N., Drennon, T., Williams, S., Stern, J., … Nguyen, Q. (2023). A robust Platform for Integrative Spatial Multi-omics Analysis to Map Immune Responses to SARS-CoV-2 infection in Lung Tissues. In bioRxiv (p. 2023.02.19.529128). https://doi.org/10.1101/2023.02.19.529128
Tian, Y., Carpp, L. N., Miller, H. E. R., Zager, M., Newell, E. W., & Gottardo, R. (2022). Single-cell immunology of SARS-CoV-2 infection. Nature Biotechnology, 40(1), 30–41. https://doi.org/10.1038/s41587-021-01131-y
Vu, T., Vallmitjana, A., Gu, J., La, K., Xu, Q., Flores, J., Zimak, J., Shiu, J., Hosohama, L., Wu, J., Douglas, C., Waterman, M. L., Ganesan, A., Hedde, P. N., Gratton, E., & Zhao, W. (2022). Spatial transcriptomics using combinatorial fluorescence spectral and lifetime encoding, imaging and analysis. Nature Communications, 13(1), 169. https://doi.org/10.1038/s41467-021-27798-0
Wang, F., Flanagan, J., Su, N., Wang, L.-C., Bui, S., Nielson, A., Wu, X., Vo, H.-T., Ma, X.-J., & Luo, Y. (2012). RNAscope: a novel in situ RNA analysis platform for formalin-fixed, paraffin-embedded tissues. The Journal of Molecular Diagnostics: JMD, 14(1), 22–29. https://doi.org/10.1016/j.jmoldx.2011.08.002
Wu, S. Z., Al-Eryani, G., Roden, D. L., Junankar, S., Harvey, K., Andersson, A., Thennavan, A., Wang, C., Torpy, J. R., Bartonicek, N., Wang, T., Larsson, L., Kaczorowski, D., Weisenfeld, N. I., Uytingco, C. R., Chew, J. G., Bent, Z. W., Chan, C.-L., Gnanasambandapillai, V., … Swarbrick, A. (2021). A single-cell and spatially resolved atlas of human breast cancers. Nature Genetics, 53(9), 1334–1347. https://doi.org/10.1038/s41588-021-00911-1
Zollinger, D. R., Lingle, S. E., Sorg, K., Beechem, J. M., & Merritt, C. R. (2020). GeoMxTM RNA Assay: High Multiplex, Digital, Spatial Analysis of RNA in FFPE Tissue. Methods in Molecular Biology , 2148, 331–345. https://doi.org/10.1007/978-1-0716-0623-0_21

**Second round of review**

**Reviewer #1**

First of all, I appreciate the substantial additional work which the authors have made for the revision. Thanks to the extensive analyses and the related discussion, I consider the manuscript has been very much improved. Even though some technical issues still remain to be further scrutinized, I understand that part should take time and would delay the publication of this manuscript in a timely manner, which I think is more important.

Even now, there are a large number of patients who are newly infected or suffering from long-term symptoms. New viral strains are still emerging. I sincerely hope further in-depth analysis of the collected rich data should lead to better understandings of the mechanism of the viral infection.

**Reviewer #2**

The revised manuscript is definitely improved and the authors' replies are on target. I certainly agree with changing the manuscript into a "Method" submission as well as the changes performed in the scope and focus of the write-up.
The following comments remain or stem from the newly added information in the revised manuscript:

Major
1) The areas of RNAscope and ST evaluated for concordance (300um diameter disks) are rather large for the specificity calculation as presented. Since both the RNAscope and ST signals are quite widely dispersed, there will be a high probability of having at least one hit for both by chance alone. I believe adding the confusion matrix could clarify whether this is the case for readers having the same question as well as adding a perturbation p-value for the observation, along with sensitivity and specificity (I believe only specificity is now provided)
2) Could you provide colocalization metrics for the SARS-CoV-2 genes within the same spots (not the disk area)? Even if some are lowly abundant, they should colocalize in the same spot if it's a real signal. Could you provide a empirical or analytical pvalues and metrics for the colocalization of the different SARS-CoV-2 targeted genes within the same spots?


Minor
1) Abstract: Please change "COVID-19 affected lung samples" into lung samples from COVID-19 patients

2) Page 3, line 65: CosMx does not require the selection of areas of interest. It can be run across a whole area of ~1cm2

3) Page 9, line 175: (r= 0.98-1) The r metric doesn't look correctly provided

**Authors Response**

**Point-by-point responses to the reviewers’ comments:**

Reviewer #1

First of all, I appreciate the substantial additional work which the authors have made for the revision. Thanks to the extensive analyses and the related discussion, I consider the manuscript has been very much improved. Even though some technical issues still remain to be further scrutinized, I understand that part should take time and would delay the publication of this manuscript in a timely manner, which I think is more important.

We thank the reviewer for acknowledging that it is important to ensure a timely publication of our work given the still large number of patients newly infected or suffering from long-term symptoms.

Even now, there are a large number of patients who are newly infected or suffering from long-term symptoms. New viral strains are still emerging. I sincerely hope further in-depth analysis of the collected rich data should lead to better understandings of the mechanism of the viral infection.

We agree with the reviewer and believe that the data collected in this study, as well as future applications of our approach, will enrich the community’s understanding of viral infection mechanisms.

Reviewer #2

The revised manuscript is definitely improved and the authors' replies are on target. I certainly agree with changing the manuscript into a "Method" submission as well as the changes performed in the scope and focus of the write-up.

We are happy to hear the reviewer finds that our proposed changes have improved the manuscript.

The following comments remain or stem from the newly added information in the revised manuscript:

Major

1) The areas of RNAscope and ST evaluated for concordance (300um diameter disks) are rather large for the specificity calculation as presented. Since both the RNAscope and ST signals are quite widely dispersed, there will be a high probability of having at least one hit for both by chance alone. I believe adding the confusion matrix could clarify whether this is the case for readers having the same question as well as adding a perturbation p-value for the observation, along with sensitivity and specificity (I believe only specificity is now provided).

We decided to compare the SARS-CoV-2 S gene signals from RNAScope and ST in 300 µm disk areas, rather than per ST spot, to account for slight shifts in signal between consecutive sections and slight shifts in the alignment of the consecutive sections. In addition, RNAScope signals can appear between the gaps of ST spots, thus comparing the signals from an area with several ST spots can provide a more reliable account of the presence of viral transcripts in a particular tissue area. Therefore, we decided to compare the signals between the two methods in more macro-areas. The confusion matrix for this quantified validation comparison can be found in Supplementary Table 3 in the Supplementary Information, Additional File 1. The confusion matrix resulted in a sensitivity of 28.93% and a specificity of 76.37%. The sensitivity (Ståhl et al. 2016) or detection efficiency (Asp, Bergenstråhle, and Lundeberg 2020) of the originally developed Spatial Transcriptomics (ST) method was ~7% of smFISH. Given that RNAScope (Wang et al. 2012) also claims single molecule detection, we would expect RNAScope to be more sensitive than ST/Visium of FFPE tissues. Thus, our calculated sensitivity of ~25% is within an expected range and could be higher than the original ST method due to using a probe-based rather than polyA-based capture approach.

A recent study compared virtual and immunohistochemistry (IHC)-based HER2 staining in a confusion matrix and used a Chi-square test to compare the degree of agreement between the virtual staining and IHC staining methods (Bai et al. 2022). Our confusion matrix compares RNAScope (considered the gold standard) and ST SARS-CoV-2 S gene signal, and, therefore, we followed a similar approach and performed a Chi-square test on the confusion matrix to compare the agreement between the two methods. We found a Chi-square test perturbation p-value for the observation of 0.323 (p-value > 0.05), indicating there is not a statistically significant difference between the viral signal detected by RNAScope and ST and thus there is no evidence to suggest a perturbation in the signal results obtained from RNAScope and ST. Such a result further supports the validation of our ST-based SARS-CoV-2 gene detection with RNAScope.

We added the confusion matrix to the Supplementary Information, Additional File 1 as Supplementary Table 3 and the sensitivity, specificity, and p-value are discussed in the manuscript Results section in lines 150-156 and the Methods section in lines 763-768.

2) Could you provide colocalization metrics for the SARS-CoV-2 genes within the same spots (not the disk area)? Even if some are lowly abundant, they should colocalize in the same spot if it's a real signal. Could you provide empirical or analytical pvalues and metrics for the colocalization of the different SARS-CoV-2 targeted genes within the same spots?

We apologize for the confusion, the provided colocalization metrics for the SARS-CoV-2 genes targeted with our approach in the previously supplied revision and now include the figure in the Supplementary Information, Additional File 1 as Supplementary Figure 11 were metrics for within the same ST/Visium spot (not the disk area). We had also previously observed that the different SARS-CoV-2 transcripts (as shown in Figure 3 of the manuscript) vary in levels (as UMI counts) with trends that largely corresponded to that expected from subgenomic RNA (sgRNA), where higher levels come from transcripts closer to the 3’ end (Kim et al. 2020; Long 2021). In Supplementary Figure 11, we can see that the portion of singleton spots (meaning spots with only one SARS-CoV-2 gene detected) also followed this trend of abundance, where more abundantly detected genes have more singleton spots.

In regards to providing the p-values for the colocalization of different SARS-CoV-2 genes within the same spot, we followed an approach similar to a recent publication that calculated the probability of the co-occurrence of different cell type pairs in RNAseq data (Muto et al. 2022). For each cell type pair, the authors constructed a matrix (present/absent × present/absent) based on the number of samples that had the presence or absence of each cell type and estimated the p-value from this matrix using a Chi-square test of independence (Muto et al. 2022). In our case, rather than cell type pairs, we constructed matrices for SARS-CoV-2 gene pairs (present/absent × present/absent) across the SARS-CoV-2+ spots (matrices contained the number of spots with both genes detected, number of spots with only gene 1 detected, number of spots with only gene 2 detected, and number of SARS-CoV-2+ spots without either gene detected) and calculated the p-value for each gene pair using both the Chi-Square test for independence and the Fisher’s exact test (to account for some frequencies being higher and some lower). The resulting p-values for all SARS-CoV-2 gene pairs are plotted in Supplementary Figure 12 in the Supplementary Information, Additional File 1.

The gene pairs with significant analytical colocalization p-values (p-value < 0.05, dotted vertical line in Supplementary Figure 12) are S-N, ORF8-N, ORF7a-ORF8, ORF7a-N, ORF1ab-S, ORF1ab-ORF8, ORF1ab-ORF3a, ORF1ab-N, ORF1ab-M. We observed that many of the significantly colocalizing genes were also the genes with higher total UMI counts and were found in a greater number of spots. Two of these genes, N and ORF8, are also closer to the 3’ end of the SARS-CoV-2 genomic RNA (gRNA) and thus are expected to have higher sgRNA abundances (Kim et al. 2020; Long 2021). ORF1ab is the only SARS-CoV-2 gene found only in gRNA and not in sgRNAs, and also significantly colocalized with five other genes (Supplementary Figure 12). Three of these genes (S, ORF3a, and M) are close in proximity to ORF1ab on the gRNA and two (N and ORF8) are closer to the 3’ end (Long 2021). Thus, the relative abundance of gRNA, sgRNAs, and the physical proximity of genes on the gRNA could be impacting our within ST spot viral RNA detection and the resulting colocalization metrics. Detection of viral RNA can also be negatively affected by degradation, which could affect both gRNA and sgRNAs, impacting which genes are detected in each spot. Having our method’s probe panels that target many SARS-CoV-2 genes, some with multiple probes, can increase the likelihood of detecting viral RNA that is impacted by degradation. Therefore, we would still consider singleton spots as real viral signals given the above-discussed biological and technical factors that can impact viral presence and detection with our approach, as well as the support of our signal detection by RNAScope and ISS validation.

The colocalization metrics and p-values are plotted in Supplementary Figures 11-12, included in the Supplementary Information in Additional File 1, and discussed in the manuscript Result section in lines 222-232, line 236, and Method section in lines 695-708.


Minor

1) Abstract: Please change "COVID-19 affected lung samples" into lung samples from COVID-19 patients

Thank you for the comment, we implemented the proposed change into the Abstract in lines 28-29.

2) Page 3, line 65: CosMx does not require the selection of areas of interest. It can be run across a whole area of ~1cm2

Thank you for pointing this out, CosMx can indeed assay larger areas. We removed CosMx from the indicated line 65 in the manuscript and instead retained our discussion of its limitation in terms of current lower gene RNA multiplexing in lines 58-64.

3) Page 9, line 175: (r= 0.98-1) The r metric doesn't look correctly provided

We apologize for any confusion, however, the “r=0.98-1” (now stated in line 181) indicates the range of Pearson correlation r-values observed across all consecutive section pairs, which ranged from r=0.98 to r=1 (as can be seen in Figure 2b and Supplementary Figure 8).

--------------------

References
Asp, Michaela, Joseph Bergenstråhle, and Joakim Lundeberg. 2020. “Spatially Resolved Transcriptomes-next Generation Tools for Tissue Exploration.” BioEssays: News and Reviews in Molecular, Cellular and Developmental Biology 42 (10): e1900221.
Bai, Bijie, Hongda Wang, Yuzhu Li, Kevin de Haan, Francesco Colonnese, Yujie Wan, Jingyi Zuo, et al. 2022. “Label-Free Virtual HER2 Immunohistochemical Staining of Breast Tissue Using Deep Learning.” BME Frontiers 2022 (January): 1–15.
Davidson, Andrew D., Maia Kavanagh Williamson, Sebastian Lewis, Deborah Shoemark, Miles W. Carroll, Kate J. Heesom, Maria Zambon, et al. 2020. “Characterisation of the Transcriptome and Proteome of SARS-CoV-2 Reveals a Cell Passage Induced in-Frame Deletion of the Furin-like Cleavage Site from the Spike Glycoprotein.” Genome Medicine 12 (1): 68.
Kim, Dongwan, Joo-Yeon Lee, Jeong-Sun Yang, Jun Won Kim, V. Narry Kim, and Hyeshik Chang. 2020. “The Architecture of SARS-CoV-2 Transcriptome.” Cell 181 (4): 914–21.e10.
Long, Samuel. 2021. “SARS-CoV-2 Subgenomic RNAs: Characterization, Utility, and Perspectives.” Viruses 13 (10). https://doi.org/10.3390/v13101923.
Muto, Kyoko, Issei Tsuchiya, Soo Hyeon Kim, Satoi Nagasawa, Mariko Takishita, Koichiro Tsugawa, Hiroaki Saito, et al. 2022. “Semibulk RNA-Seq Analysis as a Convenient Method for Measuring Gene Expression Statuses in a Local Cellular Environment.” Scientific Reports 12 (1): 15309.
Nomburg, Jason, Matthew Meyerson, and James A. DeCaprio. 2020. “Pervasive Generation of Non-Canonical Subgenomic RNAs by SARS-CoV-2.” Genome Medicine 12 (1): 108.
Ståhl, Patrik L., Fredrik Salmén, Sanja Vickovic, Anna Lundmark, José Fernández Navarro, Jens Magnusson, Stefania Giacomello, et al. 2016. “Visualization and Analysis of Gene Expression in Tissue Sections by Spatial Transcriptomics.” Science 353 (6294): 78–82.
Wang, Fay, John Flanagan, Nan Su, Li-Chong Wang, Son Bui, Allissa Nielson, Xingyong Wu, Hong-Thuy Vo, Xiao-Jun Ma, and Yuling Luo. 2012. “RNAscope: A Novel in Situ RNA Analysis Platform for Formalin-Fixed, Paraffin-Embedded Tissues.” The Journal of Molecular Diagnostics: JMD 14 (1): 22–29.

**Third round of review**

**Reviewer #2**

The revised version tackled effectively many of the major issues that became apparent from the additions of the previous cycle. There are minor issues that remain and clarifications that would benefit the manuscript but I agree with Reviewer #1 that its timely publication is of benefit of the community.

Minor

1) I would like to invite the authors to comment further on the lack of statistically significant gene co-localization. Providing critique and mentioning clearly the limitations of a technique does not reduce its utility. It points the readers to what has been achieved and stresses where further improvements can be performed.

2) If the more highly expressed genes tend also to colocalize with other genes, could that be a sign of non-specific gene expression? Could the authors perform a permutation test there as well?

**Authors Response**

**Point-by-point responses to the reviewers’ comments:**

Reviewer #2: The revised version tackled effectively many of the major issues that became apparent from the additions of the previous cycle. There are minor issues that remain and clarifications that would benefit the manuscript but I agree with Reviewer #1 that its timely publication is of benefit of the community.

We are happy to hear that our revisions effectively tackled the concerns raised by the reviewer in the previous revision cycle and that the reviewer finds our work of benefit to the community.

Minor

1) I would like to invite the authors to comment further on the lack of statistically significant gene co-localization. Providing critique and mentioning clearly the limitations of a technique does not reduce its utility. It points the readers to what has been achieved and stresses where further improvements can be performed.

We agree with the reviewer that discussing the limitations of a method does not reduce its utility. In terms of the limitations of the method in the lack of statistically significant gene colocalization for several SARS-CoV-2 gene pairs, first, we did find several SARS-CoV-2 genes significantly colocalized in our data, with many of these significantly colocalized gene pairs being located closer in proximity on the viral RNA. Other significantly colocalized gene pairs contained genes less proximal on viral RNA, however, the individual genes in the pair were more abundant in our data. It remains unclear how viral dynamics and mechanisms, FFPE induced degradation, and the detection limit of our probes (76% specificity and ~29% sensitivity of RNAScope) contribute to the lack of statistically significant gene colocalization among some SARS-CoV-2 gene pairs. Future work could improve upon the viral detection of our approach to further explore viral RNA dynamics. For example, additional probes targeting specific sequences to differentiate sgRNAs and gRNA, negative and positive sense strands, or more probes per gene could be incorporated into our method. As the method is probe-based, aspects of viral biology (mutations, modifications, etc) could affect the ability of probes to bind and detect viral RNA. Further developments of the method could attempt to combine probe-based capture of viral RNA with direct sequencing of the viral RNA, which would aid in differentiating viral strains and mutational patterns. Such enhancements to our method could improve our SARS-CoV-2 gene detection and thus elucidate additional insights into the spatial organization of SARS-CoV-2 RNA dynamics. We added these points to the Discussion section of the manuscript.

2) If the more highly expressed genes tend also to colocalize with other genes, could that be a sign of non-specific gene expression? Could the authors perform a permutation test there as well?

To address the reviewer’s suggestion on performing a permutation test, we performed a permutation test on the same SARS-CoV-2 gene pairs (present/absent × present/absent) matrices (matrices contained the number of spots with both genes detected, number of spots with only gene 1 detected, number of spots with only gene 2 detected, and number of SARS-CoV-2+ spots without either gene detected) used in our previous revision and in our revised manuscript. On the SARS-CoV-2 gene pairs matrices, we performed a permutation test of independence for a two-way contingency table (each SARS-CoV-2 gene pair matrix) based on Monte Carlo resampling (10,000 resamples) with an Approximative (Monte Carlo) Pearson chi-squared test using the coin R package (Hothorn et al. 2008). We added the p-values obtained from this permutation test based method to Figure S12 (Additional file 1: Fig. S12) and a description of our approach to the Methods section of the manuscript.

The permutation test gave similar results to the previous statistical tests, providing additional confidence to the SARS-CoV-2 genes we find significantly colocalized. To the reviewer’s point that more highly detected genes are more significantly colocalized in our data, and how this relates to non-specific gene expression, we would like to point out that in a sense SARS-CoV-2 is non-specific in the expression of its genes at the RNA level since replication produces new genomic RNAs (gRNAs) that contain all the genes and discontinuous transcription produces nested subgenomic RNAS (sgRNAs) that contain one to several genes (Long 2021). Our colocalization metrics are in line with these dynamics in that we see a more significant pairwise colocalization among genes located closer in proximity to one another on viral RNA (ORF8-N, ORF7a-ORF8, ORF7a-N, ORF1ab-S, ORF1ab-ORF3a, ORF1ab-M) (Additional file 1: Fig. S12). Other significantly colocalized gene pairs (S-N, ORF1ab-ORF8, ORF1ab-N) are less proximal, however, the individual genes in the pair are more abundant in our data (Figure 3b, Additional file 1: Fig. S12).Various factors can impact SARS-CoV-2 gene colocalization due to the highly complex nature of SARS-CoV-2 RNA replication and transcription over the course of infection that is yet to be fully understood, as discussed in several sources (Long 2021; Mori et al. 2023; Chang et al. 2021; Kim et al. 2020; Alexandersen, Chamings, and Bhatta 2020). These regulatory factors and dynamics, as well as the detection limit of our probes, could be impacting which SARS-CoV-2 genes we detect and see significantly colocalized. The mechanisms behind our observation remain to be further investigated, and we discuss improvements that can be made to our approach to further explore these questions in response to comment #1 and the Discussion of the manuscript.

References
Alexandersen, Soren, Anthony Chamings, and Tarka Raj Bhatta. 2020. “SARS-CoV-2 Genomic and Subgenomic RNAs in Diagnostic Samples Are Not an Indicator of Active Replication.” Nature Communications 11 (1): 6059.
Chang, Jessie J-Y, Daniel Rawlinson, Miranda E. Pitt, George Taiaroa, Josie Gleeson, Chenxi Zhou, Francesca L. Mordant, et al. 2021. “Transcriptional and Epi-Transcriptional Dynamics of SARS-CoV-2 during Cellular Infection.” Cell Reports 35 (6): 109108.
Hothorn, Torsten, Kurt Hornik, Mark A. van de Wiel, and Achim Zeileis. 2008. “Implementing a Class of Permutation Tests: The Coin Package.” Journal of Statistical Software 28 (November): 1–23.
Kim, Dongwan, Joo-Yeon Lee, Jeong-Sun Yang, Jun Won Kim, V. Narry Kim, and Hyeshik Chang. 2020. “The Architecture of SARS-CoV-2 Transcriptome.” Cell 181 (4): 914–21.e10.
Long, Samuel. 2021. “SARS-CoV-2 Subgenomic RNAs: Characterization, Utility, and Perspectives.” Viruses 13 (10). https://doi.org/10.3390/v13101923.
Mori, Antonio, Denise Lavezzari, Elena Pomari, Michela Deiana, Chiara Piubelli, Maria Rosaria Capobianchi, and Concetta Castilletti. 2023. “sgRNAs: A SARS-CoV-2 Emerging Issue.” Aspects of Molecular Medicine 1 (April): 100008.
